# The *Corylus mandshurica* genome provides insights into the evolution of Betulaceae genomes and hazelnut breeding

**DOI:** 10.1038/s41438-021-00495-1

**Published:** 2021-03-01

**Authors:** Ying Li, Pengchuan Sun, Zhiqiang Lu, Jinyuan Chen, Zhenyue Wang, Xin Du, Zeyu Zheng, Ying Wu, Hongyin Hu, Jiao Yang, Jianxiang Ma, Jianquan Liu, Yongzhi Yang

**Affiliations:** 1 State Key Laboratory of Grassland Agro-Ecosystem, Institute of Innovation Ecology & School of Life Sciences, Lanzhou University, Lanzhou, China; 2 Key Laboratory of Bio-Resource and Eco-Environment of Ministry of Education & State Key Laboratory of Hydraulics & Mountain River Engineering, College of Life Sciences, Sichuan University, Chengdu, China; 3 CAS Key Laboratory of Tropical Forest Ecology, Xishuangbanna Tropical Botanical Garden, Chinese Academy of Sciences, 666303 Mengla, Yunnan China; 4 Center of Plant Ecology, Core Botanical Gardens, Chinese Academy of Sciences, 666303 Mengla, Yunnan China

**Keywords:** Comparative genomics, Agricultural genetics

## Abstract

Hazelnut is popular for its flavor, and it has also been suggested that hazelnut is beneficial to cardiovascular health because it is rich in oleic acid. Here, we report the first high-quality chromosome-scale genome for the hazelnut species *Corylus mandshurica* (2*n* = 22), which has a high concentration of oleic acid in its nuts. The assembled genome is 367.67 Mb in length, and the contig N50 is 14.85 Mb. All contigs were assembled into 11 chromosomes, and 28,409 protein-coding genes were annotated. We reconstructed the evolutionary trajectories of the genomes of Betulaceae species and revealed that the 11 chromosomes of the hazelnut genus were derived from the most ancestral karyotype in *Betula pendula*, which has 14 protochromosomes, by inferring homology among five Betulaceae genomes. We identified 96 candidate genes involved in oleic acid biosynthesis, and 10 showed rapid evolution or positive selection. These findings will help us to understand the mechanisms of lipid synthesis and storage in hazelnuts. Several gene families related to salicylic acid metabolism and stress responses experienced rapid expansion in this hazelnut species, which may have increased its stress tolerance. The reference genome presented here constitutes a valuable resource for molecular breeding and genetic improvement of the important agronomic properties of hazelnut.

## Introduction

The hazelnut genus *Corylus* L., which belongs to the family Betulaceae, contains ~20 species, all bearing edible nuts^1,2^. Hazelnuts, which are a favorite among consumers, are widely used in food processing and in the manufacture of confectionery products, including chocolate, biscuits, and hazelnut oil. Hazelnuts have a high overall content of fatty acids (~60% of the hazelnut kernel), mostly oleic acid (~80% of the fatty acids), which are important dietary components for humans^3–5^. Oleic acid is usually regarded as a healthy fatty acid, as it can reduce the risk of cardiovascular disease by inhibiting cholesterogenesis in vivo as well as in vitro, reducing blood pressure, and inhibiting the atherosclerotic process^6–8^. Currently, the major hazelnut-producing countries are Turkey, Italy, Spain, and the USA, and the total annual global hazelnut production is in excess of 550,000 tons (http://www.fao.org/3/x4484e/x4484e03.htm).

Hazelnut has a long history of utilization and production, likely predating the Roman era^9^, and the most widely cultivated species have been European hazelnut (*Corylus avellana*), which has been bred mainly for high fruit yield. European hazelnut is usually susceptible to diseases such as eastern filbert blight (EFB), which can cause serious damage to the commercial production of European hazelnut^10^. During the long history of hazelnut usage, people have focused on increasing kernel size, oil content, and disease resistance^11,12^. With the development of molecular plant breeding, an increasing number of technologies are being utilized, especially CRISPR–Cas9 editing, which can greatly reduce the length of the breeding cycle and improve quality and efficiency^13^. Genes related to fatty acid biosynthesis and oleic acid accumulation are important targets for future breeding to improve the quality of the oil content. One recent study generated cotton with a high oleic acid content using the CRISPR/Cas9 system to knock out a microsomal ω-6 fatty acid desaturase gene, the product of which can catalyze the desaturation of oleic acid to form linoleic acid^14^. In the case of disease resistance, the focus is usually on disease-resistance (R) proteins, which help plants defend against a range of pathogenic organisms, including parasites, fungi, bacteria, oomycetes, insects, and viruses^15^. The largest class of disease-resistance R genes, those encoding nucleotide-binding site (NBS) proteins, have a critical role in defending plants from a multitude of pathogens and pests^16^. However, identifying such important gene resources in hazelnut has been difficult because of the lack of a high-quality genome assembly.

In China, large-scale cultivation of hazelnut is rare, but wild varieties, mainly *C. heterophylla* Fisch. and *C. mandshurica* Maxim., are distributed in mountainous forest belts and deep valleys at high altitudes in northern and northeastern China^1^. *C. mandshurica* is a deciduous shrubby Asian hazel, the bracts of which form a tubular husk with pubescence and setae; it grows naturally or is artificially cultivated across much of eastern and northern Asia. The nuts of *C. mandshurica* are characterized by a thin husk, a high kernel weight to hazelnut weight ratio, and a high concentration of oleic acid^3^. *C. mandshurica* is also highly resistant to EFB^17^, and interspecific cross-breeding trials have been carried out between *C. mandshurica* and European hazelnut to utilize the former’s valuable agronomic properties^18^. Thus, a *C. mandshurica* genome is urgently needed for use in hazel breeding.

In addition to the need to identify key gene resources in hazel, elucidating the evolutionary history of the genome across the Betulaceae is also important to help us understand the evolution of specific traits. Two subfamilies are recognized in the Betulaceae: *Alnus* (2*n* = 28) and *Betula* (2*n* = 28) form the Betuloideae, and the other four genera comprise the Coryloideae. Within the Coryloideae, *Corylus* (2*n* = 22) and *Ostryopsis* (2*n* = 16) are successively sister to *Carpinus* (2*n* = 16) and *Ostrya* (2*n* = 16)^1,19,20^. Although the Betuloideae diverged early as a monophyletic lineage, the hazelnut genus *Corylus* seems to possess many traits similar to those of the Betuloideae^21^. All of these traits indicate that the evolutionary origins of the hazelnut genus, especially chromosomal evolution, in the Betulaceae need further examination.

Although a rough genome for European hazelnut (*C. avellana*) based on Illumina sequencing has been released^22^ (https://hazelnut.data.mocklerlab.org/), the biosynthesis of oleic acid and the types of NBS in hazelnuts remain unclear due to the low quality of the assembled genome and poor annotation of the relevant genes. In this study, we constructed a high-quality chromosome-level reference genome for *C. mandshurica* by combining Illumina short reads, Nanopore long reads and chromosomal conformational capture (Hi-C) sequencing reads. The high-quality genome for a hazelnut species presented here helps us to clarify the biosynthesis of oleic acid in hazelnuts, investigate disease resistance in this species and infer the evolutionary origin of the hazelnut genus. This genomic resource also provides a valuable foundation for improving our agronomic understanding of hazelnut and carrying out molecular breeding in the future, including cloning key genes that control hazelnut traits and identifying disease-resistance alleles.

## Results

### Genome sequence and assembly

**Fig. 1:**
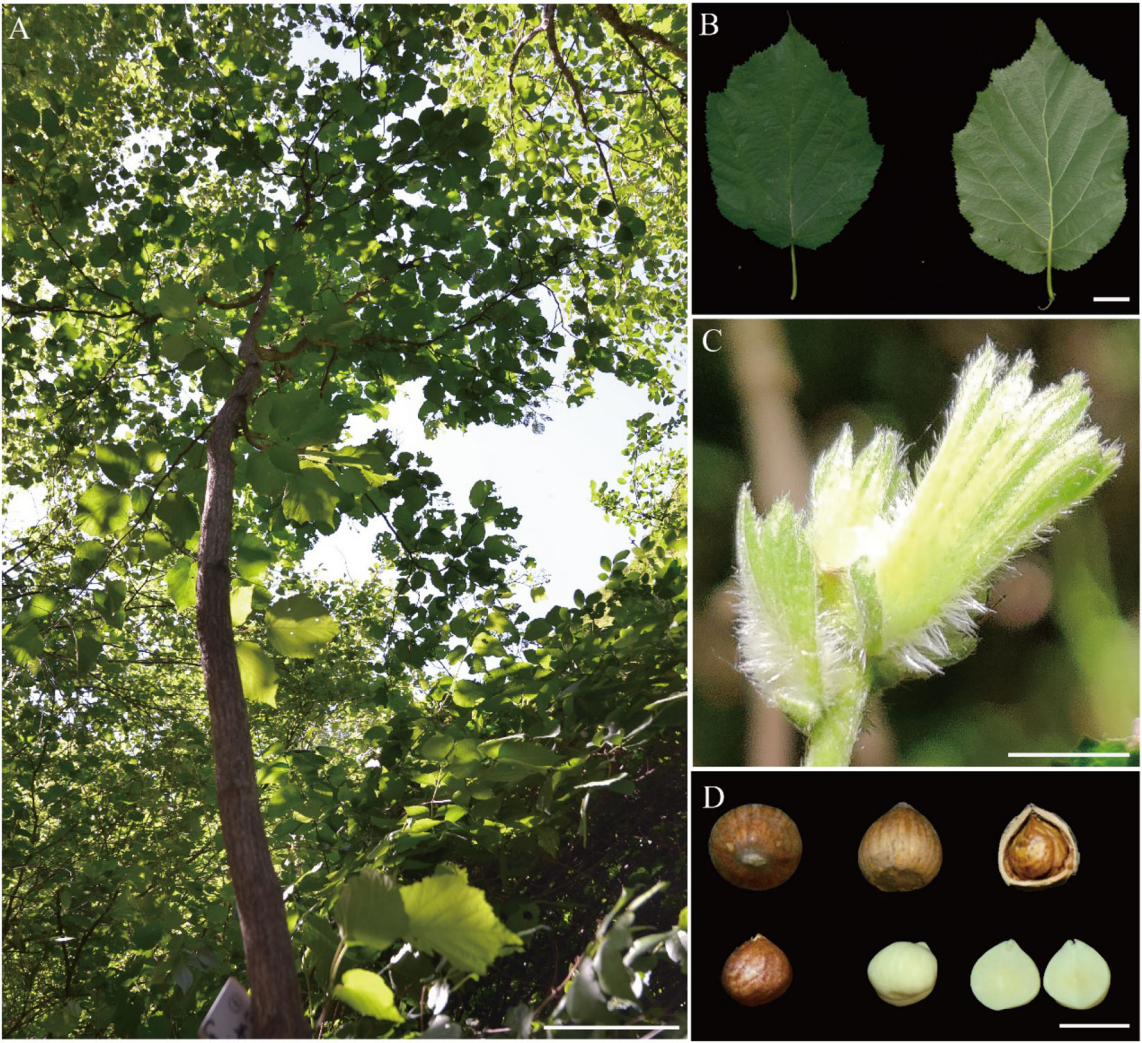
Corylus *mandshurica*, leaves, tender fruiting bracts, and hazelnuts. **A** Photograph of a mature *C. mandshurica* tree (~4.8 m) growing in Xinglong Mountain National Nature Reserve that was selected for de novo whole-genome sequencing. Scale bars (0.5 m) is shown at the bottom right. **B** Mature leaves of *C. mandshurica*, the front surface on the left, back on the right. **C** Cluster of tender fruiting bracts of *C. mandshurica* with obvious pubescence and setae. **D** Nuts of *C. mandshurica*. Top panel from left to right: top view, lateral view, and lateral view of nut in the shell. Bottom panel from left to right: lateral view of the nut with spermoderm, lateral view of the kernel, and cross-section of nut. Scale bars (1 cm) are shown at the bottom right in **B**–**D**

A mature *C. mandshurica* tree growing in Xinglong Mountain National Nature Reserve was selected for de novo whole-genome sequencing (Fig. 1). A total of 38.78 Gb (~98.90×, Supplementary Table 1) of Illumina clean short reads was retrieved and used to perform a genome survey, which indicated that the size of the *C. mandshurica* genome was 392.16 Mb and that it has relatively high heterozygosity (~0.92%) (Supplementary Fig. 1). The estimated genome size is very close to that previously reported for European hazelnut^22^ (378 Mb). A total of 38.08 Gb (~97.12×, Supplementary Table 1) of raw Nanopore long reads were self-corrected, assembled, and polished with NextDenovo and Nextpolish^23^. After deletion of candidate allelic haplotigs, the final contig-level assembly of 367.67 Mb was obtained with a contig N50 of 14.85 Mb (accounting for 93.7% of the estimated genome size; Table 1 and Supplementary Table 2). To evaluate our assembly, first, Benchmarking Universal Single-Copy Orthologs^24^ (BUSCO) was selected, and the results showed that 97.1% of conserved genes could be completely annotated within our genome, which is more than has been achieved for other published Betulaceae species^22,25–29^ (Supplementary Table 3). The base accuracy of the assembly was also estimated based on the mapping of Illumina reads. We aligned genome sequencing reads and transcriptome sequencing reads to the *C. mandshurica* genome, with mapping rates of 98.86% and 99.68%, respectively (Supplementary Table 4). The RNA transcripts were further aligned, and 97.00%–99.37% of transcripts could be mapped with over more than half their length to the same contig (Supplementary Table 5). These results collectively suggest that the *C. mandshurica* genome is well assembled and of high quality.

**Table 1:** Statistical details of genome assembly and annotation

Assembly	Size (bp)
Genome assembly	
Contig N50	14,849,403
Contig N90	3,939,978
Longest contig	22,635,284
Total contig length	367,672,720
BUSCO (complete)	97.1%
Genome annotation	
No. of predicted protein-coding genes	28,409
Average gene length	3561.82
Mean length of exons per gene	5.0
Masked repeat sequence length	252,743,315
Percentage of repeat sequence	68.74%
Hi-C assembly	
Scaffold N50	36,270,330
Scaffold N90	22,830,127
Longest scaffold	52,982,101
Total scaffold length	367,672,720

**Fig. 2:**
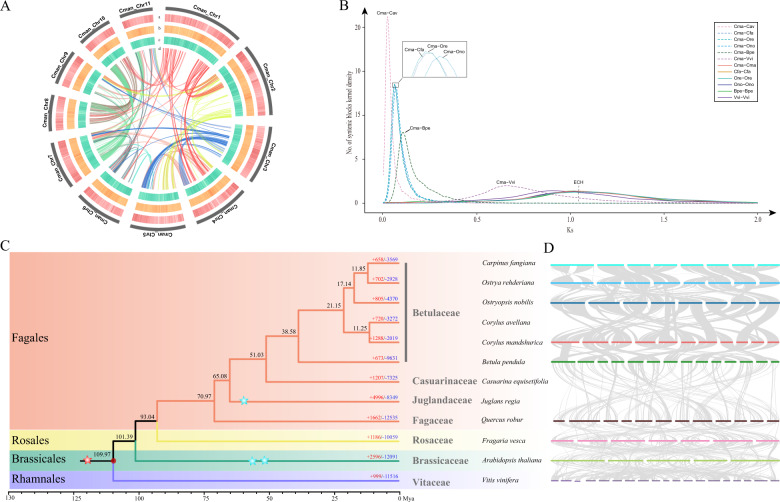
Genome features and evolutionary and comparative genomic analyses. **A** Mapped features of the *C. mandshurica* genome, including a GC (guanine-cytosine) content, b repeat density, c gene density, d synteny information, Cma *C. mandshurica*. **B** Ks distribution of syntenic blocks between and within Betulaceae species. Bpe: *B. pendula*; Cav: *C. avellana*; Cfa: *Ca. fangiana*; Cma: *C. mandshurica*; Ono: *Ostryopsis nobilis*; Ore: *Ostrya rehderiana*; Vvi: *Vitis vinifera*; ECH: Eudicot-common hexaploidy. The enlarged display of the peak of the curve for the three Ks plots is shown in the black box. **C** Phylogenomic tree and expansion and contraction of gene families among *C. mandshurica* and 11 other species. The calibration time is marked by a red point. The blue stars and red stars represent the known whole-genome duplication and whole-genome triplication events identified previously, respectively. **D** MCscanX identified synteny blocks (involving ≥ 5 collinear genes) between nine species containing chromosome-scale genomes. The species names correspond to those in **C**

We also generated 42.04 Gb of clean reads from Hi-C sequencing, giving ~114.43× of the *C. mandshurica* genome (Supplementary Table 6). The quality of Hi-C data was assessed by mapping the reads to the assembled genome, with 92.3% of reads being mapped to the assembled contigs, and the unique mapped read pair percentage was 50.5% (Supplementary Table 6). Valid interaction pairs were integrated from the unique paired alignments, and all the contigs were categorized and ordered to construct chromosome-scale scaffolds, resulting in 11 *C. mandshurica* pseudomolecules totaling 367.67 Mb with a scaffold N50 of 36.3 Mb (Table 1, Fig. 2A, and Supplementary Fig. 2). The longest and shortest pseudomolecules were chromosomes 1 and 11, with lengths of 52.98 and 21.94 Mb, respectively (Supplementary Table 7).

### Genome annotation

The *C. mandshurica* genome was found to contain 252.7 Mb (68.74%) transposable elements (TEs) (Fig. 2A and Table 1), specifically, 63.48% retrotransposons and 4.39% DNA transposons (Supplementary Table 8). The dominant repetitive sequence type is long terminal retrotransposons (LTRs), forming 57.92% of the repetitive sequences, whereas 19.95 Mb (5.46%) of the genome consists of non-LTR elements (Supplementary Table 8). A combination of transcriptomics, homology information, and a de novo approach were used to accurately predict gene models. We predicted 28,409 genes in the *C. mandshurica* genome (Fig. 2A and Supplementary Table 9). The average protein-coding gene size was 3562 bp, and the mean exon number was 5.0 (Table 1). The predicted protein-coding genes spanned a total length of 101.19 Mb and were anchored to the 11 chromosomes (Fig. 2 and Supplementary Table 9). In addition, 1327 (92.2%) BUSCO^24^ genes could be completely matched to our predicted *C. mandshurica* gene set (Supplementary Table 10). Among the 28,409 genes predicted, 26,309 (92.6%) were functionally annotated from five databases of known proteins: TrEMBL^30^ (83.3%), SWISS-PROT^30^ (66.4%), Gene Ontology (GO, 56.0%), Kyoto Encyclopedia of Genes and Genomes^31^ (KEGG, 28.4%) and InterPro (92.6%) (Supplementary Table 11). Additionally, 1366 transcription factors were detected in the *C. mandshurica* genome (Supplementary Table 12). We used transcription factors from *C. avellana* for further comparison. We found that four transcription factor families were significantly expanded (*p* < 0.01) in *C. mandshurica* compared to *C. avellana*. Three of these families were related to stress responses: APETALA 2/ethylene response factor (AP2/ERF-ERF)^32^, calmodulin-binding transcription activator (CAMTA)^33^, and v-myb avian myeloblastosis viral oncogene homolog (MYB)^34^. The basic/helix–loop–helix (bHLH)^35^ family, which is related to cell proliferation, was also significantly expanded in *C. mandshurica* (Supplementary Table 12). Noncoding RNAs were annotated, giving predictions of 195 transfer RNA (tRNA) genes, 336 ribosomal RNA (rRNA) genes, 329 small nuclear RNA genes, and 83 microRNA (miRNA) genes (Supplementary Table 13).

### Genome evolution

To explore genome evolution in *C. mandshurica*, genes from 12 species in total, including five species of Betulaceae (*Betula pendula*^25^, *C. avellana*^22^, *Carpinus fangiana*^26^, *Ostrya rehderiana*^27^, and *Ostryopsis nobilis*^28^), three species of other Fagales (*Casuarina equisetifolia*^36^, *Juglans regia*^37^, and *Quercus robur*^38^), *Arabidopsis thaliana*^39^, *Fragaria vesca*^40^, and *Vitis vinifera*^41^, were clustered into 31,991 gene families (Supplementary Fig. 3). Out of these, we identified 1628 single-copy genes and reconstructed a phylogenetic tree with this gene set (Fig. 2C). This result revealed that *C. mandshurica* and *C. avellana* diverged from one another ~11.25 million years ago (Mya), placing them in the basal position in the Coryloideae, and diverged from the Coryloideae ~21.15 Mya. Betulaceae were estimated to have diverged from the other three Fagales species, casuarina, walnut, and oak, ~51.03, ~65.08, and ~70.97 Mya, respectively. The Ks distribution was determined using syntenic paralogs from each genome. The Ks value for collinear gene pairs indicated that there had been no recent whole-genome duplication in any of the six Betulaceae species, but the eudicot-common hexaploidy event was observed (Fig. 2B).

### Inference of karyotype evolution in Betulaceae

**Fig. 3:**
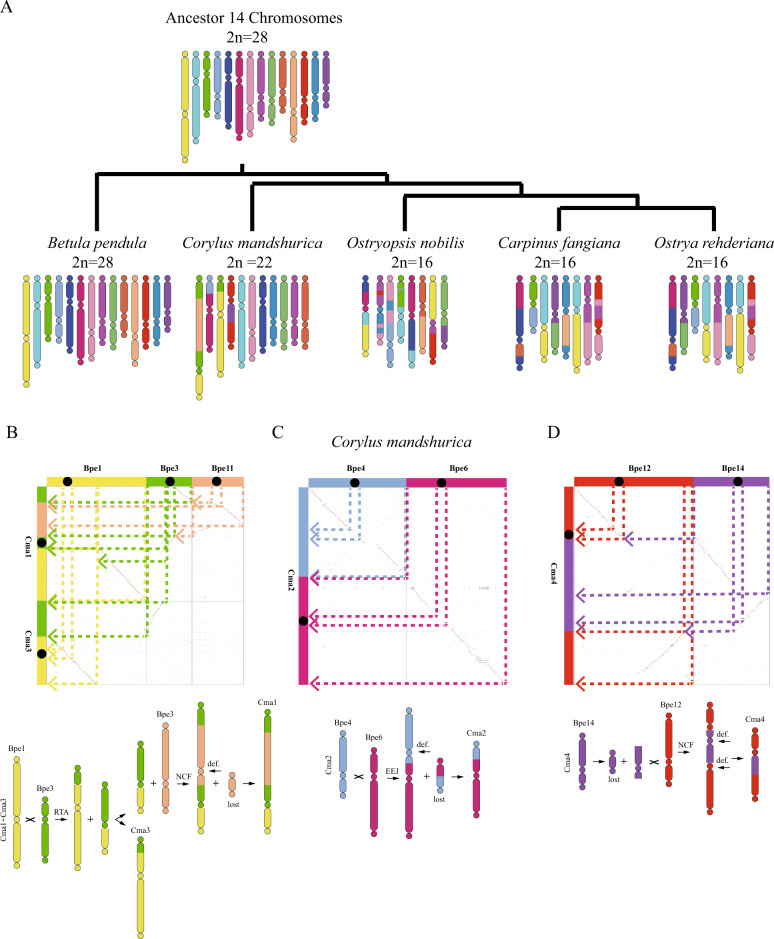
Schematic representation of karyotype evolution in the five Betulaceae genomes from the ancestral Betulaceae karyotype and chromosome fusions/fissions during the karyotype evolution of *C. mandshurica*. The 14 ancestral chromosomes from *B. pendula* (Bpe1–Bpe14), shown as rectangular blocks in different colors, were used as references, and their relationships to the chromosomes in *C. mandshurica* are indicated. Bpe: *B. pendula*; Cma: *C. mandshurica*. RTA: reciprocally translocated chromosome arms; NCF: nested chromosome fusion; EEJ: end-end joining. **A** Scenario for the evolution of the Betulaceae genomes from the ancestral karyotype. **B** Process of construction of chromosomes Cma1 and Cma3. **C** Process of construction of chromosome Cma2. **D** Process of construction of chromosome Cma4

Genomic changes can be detected and relative chronometry established by parsimony-based phylogenomic analysis, and evolutionary trajectories of karyotypes can also be elucidated in this way. Here, to reveal the karyotype evolutionary trajectories of Betulaceae, we identified syntenic conservation and chromosome rearrangements among the five genomes of *B. pendula* (Bpe)*, C. mandshurica* (Cma)*, Ca. fangiana* (Cfa)*, Ostrya rehderiana* (Ore), and *Ostryopsis nobilis* (Ono) to represent the genera *Betula*, *Corylus*, *Carpinus*, *Ostrya*, and *Ostryopsis*, respectively (Fig. 3 and Supplementary Figs. 4–7). Putative homologous genes and collinear genes were identified within each genome and between each pair of genomes (Fig. 2D), and dot plots for species within Betulaceae were employed to depict orthology information (Supplementary Figs. 4 and 5).

*B. pendula* was identified as the most primitive species among these five Betulaceae based on previous studies, so the karyotype of *B. pendula* should be the closest to the ancestral karyotype^21,42,43^. We, therefore, selected *B. pendula* as the reference to identify syntenic blocks across these genomes. Analysis of syntenic relationships showed that the integrity of all chromosomes except Bpe1 and Bpe3 of *B. pendula* was essentially preserved in *C. mandshurica*, and seven chromosomes showed one-to-one correspondence between the two species (Supplementary Fig. 4). There were no corresponding chromosome pairs between *B. pendula* and *Ca. fangiana*, *Ostrya rehderiana* or *Ostryopsis nobilis*, but all of them showed clear evidence for retention of complete *B. pendula* chromosomes (Supplementary Figs. 5–7). Large regions of chromosome segments shared by extant genomes can be used to infer the identities of other protochromosomes. However, there were no shared chromosome segments even though several *B. pendula* chromosomes (chromosomes 1, 3, 4, 6, 11, 12, and 14) appeared as rearrangements in all four other species (Fig. 3 and Supplementary Figs. 4–7), indicating that *B. pendula* chromosomes correspond to the ancestral karyotype of Betulaceae and have not undergone large chromosome segment rearrangements.

As explained above, we used *B. pendula* chromosomes as the ancestral karyotype to infer evolutionary trajectories for each Coryloideae species. The formation of *C. mandshurica* chromosome 1 (Cma1) could be clearly inferred as having occurred by a fusion of Bpe1 and Bpe3 and a nested chromosome fusion (NCF) of Bpe11 (Fig. 3B). The NCF process could have occurred as follows: Bpe1 and Bpe3 reciprocally translocated chromosome arms (RTA) and formed Cma3 and the precursor of Cma1; Cma3 consisted of part of the short arm of Bpe3 and most of Bpe1, while the precursor of Cma1 consisted of part of the short arm of Bpe1 and the long arm of Bpe3; then Bpe11 underwent crossing over to form a major chromosome and a satellite chromosome; the satellite chromosome may have been lost, while the major chromosome was inserted into the Bpe3 long arm region of the precursor of Cma1. Cma2 was formed from Bpe4 and Bpe6 by end-end joining (EEJ) (Fig. 3C), and Cma4 was formed by an NCF of Bpe14 into Bpe12 (Fig. 3D).

During the process of construction of *C. mandshurica* chromosomes, two intrachromosome telomere-proximal crossings occurred to generate two free-end intermediate chromosomes and two satellite chromosomes (Fig. 3B–D); the former fused into the pericentromeric regions of other chromosomes, while the satellite chromosomes may have been lost. Moreover, two interchromosome telomere-proximal crossings occurred to generate an end-end merged chromosome, two fusion chromosomes, and a satellite chromosome. The final chromosome number was reduced from 14 to 11 in *C. mandshurica*. Except for the genus *Corylus*, all the genera of Coryloideae (*Carpinus*, *Ostryopsis*, and *Ostrya*) had the same chromosome number (*n* = 8), and *Ca. fangiana* and *Ostrya rehderiana* also showed the same karyotype evolution trajectory (Supplementary Fig. 6), consistent with the predicted phylogenetic relationships. Both of them experienced two NCF, four EEJ, and four RTA events and lost six satellite chromosomes to produce the eight extant *Ca. fangiana* (*Ostrya rehderiana*) chromosomes (Supplementary Fig. 6). *Ostryopsis nobilis* exhibited the most complex evolutionary process, including seven EEJ and seven RTA events and the loss of five satellite chromosomes to form the extant karyotype (Supplementary Fig. 7).

To gain a further understanding of karyotype evolution in these Betulaceae species, we identified genes located within 10 kb around chromosome fission, fusion, or rearrangement events. A total of 19, 25, 33, and 39 genes were extracted in *C. mandshurica*, *Ca. fangiana* and *Ostrya rehderiana* and *Ostryopsis nobilis*, respectively. Among them, 2/1/1 genes related to chromosome structure were found in *C. mandshurica*, *Ostrya rehderiana*, *Ostryopsis nobilis*, respectively (Supplementary Table 14). We speculate that chromosome structure variations may cause genome instability, so related genes such as telomere protection and DNA repair are crucial. We did not detect genes that might be related to the traits that differ among Betulaceae species, such as kernel size, leaf properties, and adaptability. This may be because literature data on their functions are rare and further studies are needed, or it may be that the evolution of such traits is decided mainly by other mechanisms.

### Gene family evolution

In *C. mandshurica*, 80 gene families comprising 290 genes exhibited significant rapid expansion relative to the ancestor of the hazels. Some of these families were annotated as phenylpropanoid metabolic process, aromatic compound catabolic process, regulation of salicylic acid-mediated signaling pathway, regulation of response to stress, and regulation of defense response (Supplementary Table 15). These genes may be a resource for investigating features specific to *C. mandshurica*.

We further identified R genes, which have an essential role in plant disease defense signaling^15,16^, in 12 species (Supplementary Table 16). A total of 80 R genes containing the NBS domain were identified in the *C. mandshurica* genome; this is an intermediate number of NBS-containing genes (Supplementary Table 16 and Supplementary Fig. 8). Moreover, a cross-species comparison of numbers of R genes indicated that all Betulaceae species (49–116) except *C. avellana* (301) had far fewer than *A. thaliana* (178), *Cas. equisetifolia* (140), and *J. regia* (88). The higher number of R genes within *C. avellana* maybe because the genome was obtained from an EFB-resistant breed (Jefferson)^44,45^. We also found larger gene numbers of two types of NBSs (CC-NBS-LRR [CNL] and NBS-only) in both *C. mandshurica* and “Jefferson”. CNL is one of the two major plant NBS proteins involved in pathogen recognition, and NBS-only genes may act as adaptors or regulators of CNL and TIR-NBS-LRR proteins^16^. These genes may be important genetic resources for increasing disease resistance.

### Evolution of genes involved in oil biosynthesis

**Fig. 4:**
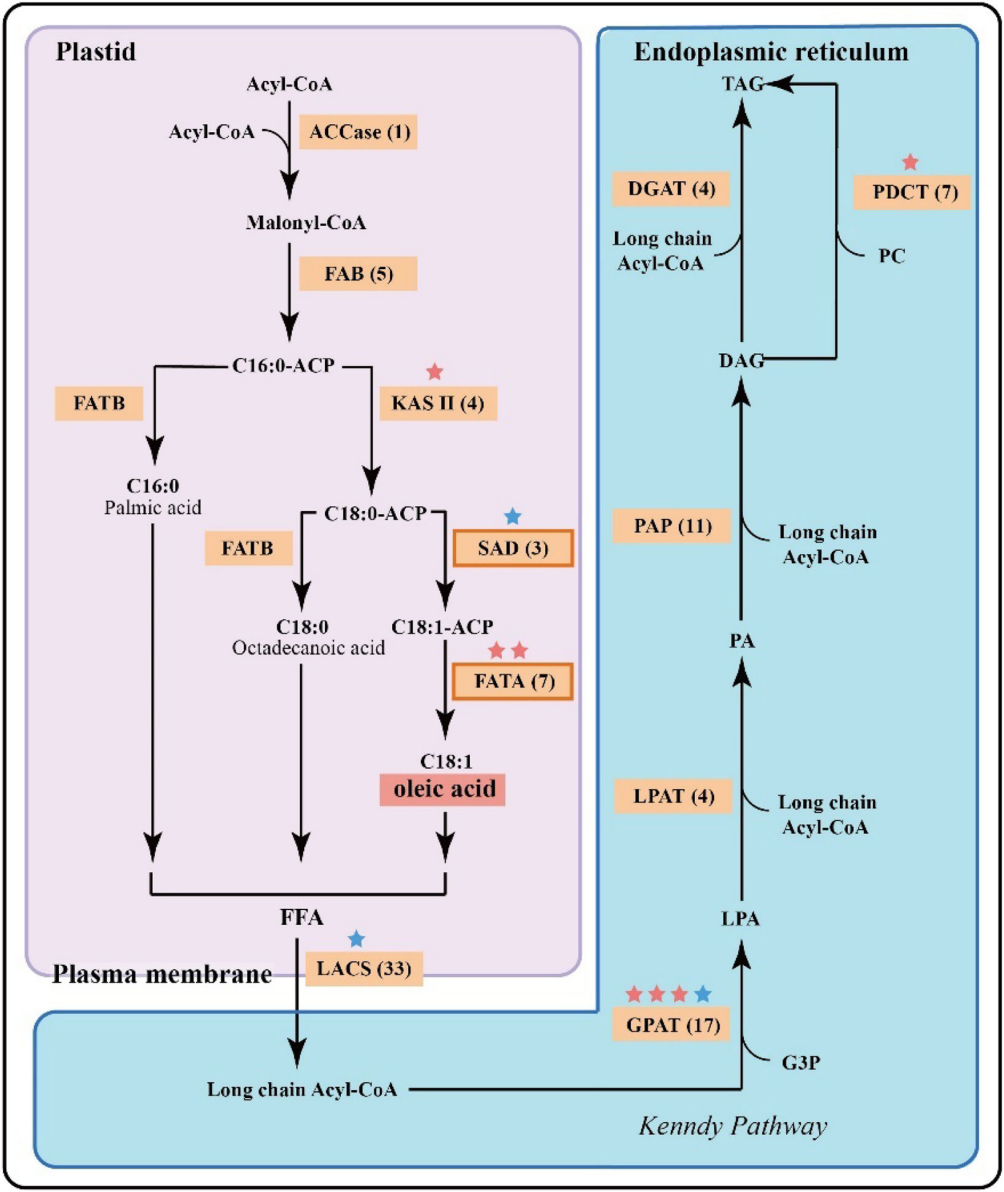
Metabolic pathways and numbers of genes associated with biosynthesis and accumulation of oleic acid and TAG in *C. mandshurica*. Enzymes that participate in the de novo synthesis of free fatty acids and triacylglycerols (TAGs) are shown in rectangles, and key enzymes in oleic acid biosynthesis are indicated by boxes outlined in orange. The number of genes encoding each type of enzyme is shown in brackets. The red and blue stars indicate the enzymes under rapid evolution and positive selection, respectively. Acyl-CoA acyl-coenzyme A, DAG diacylglycerol, DGAT diacylglycerol acyl-transferase, FAB fatty acid biosynthesis, FATA fatty acyl-ACP thioesterase A, FATB fatty acyl-ACP thioesterase B, G3P glycerol-3 phosphate, GPAT glycerol-3-phosphate acyl-transferase, KAS II β-ketoacyl-[acyl carrier protein] synthase II, LACS long-chain acyl-CoA synthase, LPA lysophosphatidic acid, LPAT lysophosphatidyl acyl-transferase, PAP phosphatidic acid phosphatase, PA phosphatidic acid, PC phosphatidylcholine, PDCT phosphatidylcholine:diacylglycerol cholinephosphotransferase, SAD stearoyl ACP desaturase

Biosynthesis of oleic acid starts from acetyl-CoA and is catalyzed by multiple isozymes involved in 5 enzymatic steps, generating free oleic acid (Fig. 4). Then, long-chain acyl-CoA synthase (LACS) enzymes catalyze the synthesis of long-chain acyl-CoAs that feed into the Kennedy pathway, leading to the production of triacylglycerols^46–49^.

A total of 764 oil biosynthesis-related genes were identified in the *C. mandshurica* genome (Supplementary Table 17). Of these, 96 were identified as participating directly in oil biosynthesis (Fig. 4, Table 2, and Supplementary Fig. 9): including one acetyl-CoA carboxylase (ACCase) gene, five fatty acid biosynthase (FAB) genes, four β-ketoacyl-[acyl carrier protein] synthase II (KAS II) genes, three stearoyl-CoA desaturase (SAD) genes, seven fatty acyl-acyl carrier protein thioesterase A (FATA) genes, 33 long-chain acyl-CoA synthase (LACS) genes, 17 glycerol-3-phosphate acyltransferases (GPAT) genes, four lysophosphatidic acid acyl-transferase (LPAT) genes, 11 phosphatidate phosphatase (PAP) genes, four acyl-CoA: diacylglycerol acyl-transferase (DGAT) genes and seven phospholipid: diacylglycerol acyl-transferase (PDCT) genes. The number of fatty acid biosynthesis-related genes was within the range found in other plant species; there were 86 such genes in *A. thaliana*, 62 in *B. pendula*, 76 in *C. avellana*, 102 in *Ca. fangiana*, 96 in *Cas. equisetifolia*, 130 in *J. regia*, 81 in *Ostrya rehderiana* and 87 in *Ostryopsis davidiana* (Table 2). Of the proteins encoded by these genes, FATA and SAD are key enzymes in oleic acid biosynthesis (Fig. 4). There are 7 FATA and 3 SAD genes in the *C. mandshurica* genome, 4 FATA and 3 SAD genes in the *A. thaliana* genome, and 10 FATA and 3 SAD genes in the walnut genome. Thus, the number of SAD genes in *C. mandshurica* is higher than that in *A. thaliana* but lower than that in walnut. The number of fatty acid biosynthesis-related genes in European hazelnut apparently exceeds only the number in *B. pendula*. The quality of the genome assembly may have affected this result, but FATA and SAD genes are still more numerous in European hazelnut than in *A. thaliana*.

**Table 2:** Cross-species comparison of the number of fatty acid biosynthesis-related gene families

	Ath	Bpe	Cav	Ceq	Cfa	Cma	Jre	Ono	Ore
*ACCase*	2	1	1	3	2	1	2	1	1
*FAB*	6	3	4	9	12	5	8	9	3
*KAS II*	3	1	4	6	9	4	7	4	4
*SAD*	2	2	2	2	4	3	3	2	2
*FATA*	4	4	6	7	5	7	10	7	4
*LACS*	23	10	25	21	25	33	37	23	26
*GPAT*	20	18	15	20	18	17	30	18	18
*LPAT*	4	3	3	6	5	4	6	4	4
*PAP*	9	8	8	13	11	11	14	8	9
*DGAT*	7	3	2	1	4	4	4	3	3
*PDCT*	6	9	6	8	7	7	9	8	7
Total	86	62	76	96	102	96	130	87	81

*Ath* *A. thaliana*, *Bpe* *B. pendula*, *Cav* *C. avellana*, *Ceq* *Cas. equisetifolia*, *Cfa* *Ca. fangiana*, *Cma* *C. mandshurica*, *Fve* *F. vesca*, *Jre* *J. regia*, *Ono* *Ostryopsis nobilis*, *Ore* *Ostrya rehderiana*, *Acyl-CoA* acyl coenzyme A, *DAG* diacylglycerol, *DGAT* diacylglycerol acyl-transferase, *FAB* fatty acid biosynthesis, *FATA* fatty acyl-ACP thioesterase A, *FATB* fatty acyl-ACP thioesterase B, *G3P* glycerol-3 phosphate, *GPAT* glycerol-3-phosphate acyl-transferase, *KAS II* β-ketoacyl-[acyl carrier protein] synthase II, *LACS* long-chain acyl-CoA synthase, *LPA* lysophosphatidic acid, *LPAT* lysophosphatidyl acyl-transferase, *PAP* phosphatidic acid phosphatase, *PA* phosphatidic acid, *PC* phosphatidylcholine, *PDCT* phosphatidylcholine:diacylglycerol cholinephosphotransferase, *SAD* stearoyl ACP desaturase

### Rapidly evolving and positively selected genes

To better reveal the evolutionary dynamics of *C. mandshurica* and identify more functional gene resources, we further identified the rapidly evolving and positively selected genes within *C. mandshurica* by comparison with the other four Betulaceae species (*B. pendula*, *Ca. fangiana*, *Ostryopsis nobilis*, and *Ostrya rehderiana*) and *Cas. equisetifolia*. A total of 8624 high-confidence single-copy orthologs were obtained with a trimmed alignment length from 102 to 14,455 bp. The free-ratio model in PAML^50^ was first employed to estimate the independent Ka/Ks ratio of each species based on all genes. We compared the overall whole-genome average Ka/Ks between six species and found that *C. mandshurica* showed a moderate evolutionary rate (Supplementary Fig. 10). In contrast, *B. pendula* has the highest Ka/Ks ratio, possibly due to its short life history as a pioneer boreal tree^25^. We also identified 1327 rapidly evolving genes (REGs) and 1066 positively selected genes (PSGs) in *C. mandshurica*. Both types of genes were functionally enriched in organic compound metabolic activities, with 165 PSGs and 78 REGs involved in the heterocycle, carbohydrate, organic cyclic compound, and cellular aromatic compound metabolic processes (Supplementary Fig. 11). REGs further exhibit enrichment in glycerolipid biosynthetic and tetrapyrrole binding, which are associated with hazelnut flavor. Glycerolipids are the major nutrient component of hazelnut^3–5^, and organic cyclic compounds, especially pyrroles, are highly related to the volatile compounds of hazelnut^51^. Within the oil biosynthesis pathway, three GPAT, SAD, and LACS genes were detected to be under positive selection, and three GPAT genes, two FATA genes, one KAS II gene, and one PDAT gene were found to be under rapid evolution (Supplementary Table 18). Both SAD and FATA, as the key enzymes in oleic acid biosynthesis^52^, may underlie the high oleic acid concentration in hazelnuts. Moreover, within three significantly expanded transcription factor families that are involved in stress responses, i.e., the AP2/ERF-ERF, MYB, and CAMTA gene families, 11, 3, and 1 genes, respectively, were found to be positively selected; In the former two families, 15 and 6 genes, respectively, showed rapid evolution (Supplementary Table 18). Additionally, two R genes (RCNL type and NBS) were identified to be rapidly evolving (Supplementary Table 18). These rapidly evolving and positively selected genes may have contributed to the high environmental adaptability of *C. mandshurica*.

## Discussion

The chromosome-level *C. mandshurica* genome assembly reported here has the highest quality among the recently published Betulaceae species genomes (Supplementary Table 3), with the longest contig N50 (14.8 Mb) and the highest genome completeness (97.2%) in terms of BUSCO results. It could also be compared with the recently published genomes and at the top level (Supplementary Table 3). This high-quality genome may help us to identify biosynthetic pathways for oleic acid in hazelnuts, investigate the abiotic tolerance of this species and infer the evolutionary pathway of chromosomes of the hazelnut genus.

Hazelnut is one of the four major tree nuts consumed globally. Recent studies found that hazelnut oil is a good supplier of fatty acids, particularly oleic acid, which carries health benefits^3,4^. Although fatty acid synthesis mechanisms have been extensively studied in plants^46–48^, we identified candidate genes for the biosynthesis of oleic acid in hazel for the first time. These resources will be valuable in functional genomics studies and the improvement of traits that are economically important in hazelnut, such as nutrition and flavor. They included 96 genes whose products were identified as participating directly in the biosynthesis of oleic acid. Enzymes encoded by the latter group included ACCase, which is most likely the key enzyme determining the metabolic pathways that lead to oil or protein biosynthesis in the seed^49,53^. FATA and SAD are probably key components in oleic acid accumulation^54^, and two FATA genes and one SAD gene showed rapid evolution and positive selection, respectively. Candidate genes related to fatty acid biosynthesis and undergoing rapid evolution or positive selection are highly valuable for genetic improvement of hazelnut in the future.

Stress resistance is among the most important traits in hazel breeding programs. *C. mandshurica* has a greater tolerance of fungal infection than cultivated forms of European hazel^17^. Overall, 80 genes with an NBS-coding region were identified in the *C. mandshurica* genome, whereas 301 were identified in European hazel, which has been bred for increased fruit output and disease resistance. We speculate that this is because the European hazel genome sequence was obtained from the EFB-resistant diploid hazelnut accession “Jefferson”, which may have retained a high number of NBS genes. Salicylic acid is a phytohormone that regulates signal transduction pathways involved in defense against biotic and abiotic stresses^54^, and our evolutionary analysis found that several gene families related to salicylic acid metabolism and stress response had undergone rapid expansion in *C. mandshurica*. Moreover, three out of the four transcription factors in families that had significantly expanded in *C. mandshurica* were related to stress responses. Of these, MYB proteins are key factors in regulatory networks controlling development^34^, metabolism, and responses to biotic and abiotic stresses, AP2/ERF-ERFs have important functions in the transcriptional regulation of various responses to environmental stimuli^32^, and CAMTA transcription factors are master regulators of salicylic acid-mediated immunity^54^. In addition, we identified several genes under rapid evolution or positive selection within these three TF families: MYB, AP2/ERF-ERF, and CAMTA. Taken together, these results indicate that NBS may not be the only key factor underlying the high-stress resistance of *C. mandshurica*; several other transcription factors may also contribute to this feature.

The rearrangement of centromeres and telomeres can result in changes in chromosome number^55^. The evolutionary trajectories of the Betulaceae chromosomes were reconstructed in this study. Previous studies have consistently agreed that the Betuloideae are more primitive than the Coryloideae in Betulaceae^21,42,43^, and indeed, *B. pendula* was located at the basal position in the phylogenetic relationship of these species. Thus, we considered that the *B. pendula* genome is the most primitive karyotype among the set of genomes currently available for Betulaceae species and used this genome as a reference. We used collinearity and phylogenetic analysis of the five Betulaceae species to provide evidence in support of an evolutionary scenario in which the most ancestral karyotype of the Betulaceae species investigated is that of *B. pendula*. Moreover, we found that the chromosomes of *B. pendula* are essentially preserved in the other four Betulaceae karyotypes, and in particular, a one-to-one correspondence between *B. pendula* and *C. mandshurica* further confirmed that *B. pendula* represents the ancestral karyotype. *C. mandshurica* occupies the second place because of the occurrence of only 2 NCFs, 1 chromosome end-end merge, 1 RTA, and the loss of 3 satellite chromosomes. Integration of phylogenomic and collinearity analyses of Betulaceae genomes further revealed that chromosomes evolved along three completely different trajectories within different genera of Coryloideae. Compared to *Corylus, Ostryopsis–Carpinus–Ostrya* showed a more sophisticated trajectory, and *Carpinus* and *Ostrya* had a consistent trajectory; all of these findings were in accordance with their phylogenetic relationships. With the availability of *Alnus* genome sequences, it will be possible to characterize the karyotype evolutionary trajectory for Betulaceae genomes in greater depth.

In summary, we obtained a high-quality chromosome-level reference genome for the hazelnut species *C. mandshurica* and identified candidate genes related to oleic acid biosynthesis and stress tolerance in this species. Our genomic resources will guide hazelnut breeders in utilizing the excellent genetic resources of *C. mandshurica* and accelerate our understanding of genome evolution within the Betulaceae.

## Materials and methods

### DNA extraction and sequencing

Fresh young leaves were collected from a wild *C. mandshurica* tree growing in Xinglong Mountain National Nature Reserve in Lanzhou, Gansu Province, China (35°47′20.83″ N, 104°6′16.23″ E, 2290 m). Genomic DNA was extracted using a QIAGEN Genomic DNA extraction kit according to the standard operating procedure provided by the manufacturer. Quality control of the extracted DNA was carried out using a NanoDrop One UV-Vis spectrophotometer (ThermoFisher Scientific, USA) to check the DNA purity (OD260/280 ranging from 1.8 to 2.0 and OD260/230 between 2.0 and 2.2), and then a Qubit 3.0 A fluorometer (Invitrogen, USA) was used to accurately quantify the DNA. Since the sample was of adequate quality, a paired-end library with an insert size of 400 bp was constructed using the standard Illumina protocol with the HiSeq X Ten platform. Following the Nanopore library construction protocol, a Nanopore library was constructed, and long-read data were generated using the PromethION sequencer platform (Oxford Nanopore Technologies, UK). Sequencing adapters were removed, and reads of low quality and short length were filtered out.

For Hi-C library construction, fresh young leaves from the same *C. mandshurica* tree were ground in liquid nitrogen. Chromatin was fixed using formaldehyde. Then, leaf cells were lysed, and the fixed chromatin was digested by the endonuclease DpnII. The 5′ overhangs of the DNA were recovered with biotin-labeled nucleotides, and the resulting blunt ends were ligated to each other using DNA ligase. Proteins were removed with protease to release the DNA molecules from the crosslinks. The purified DNA was sheared into 350-bp fragments and ligated to adaptors. The fragments labeled with biotin were extracted using streptavidin beads, and after PCR enrichment, the libraries were sequenced on the Illumina HiSeq X Ten platform.

### Genome assembly and pseudochromosome construction

Before genome size estimation, we first filtered the short Illumina reads using fastp^56^ (v.0.20.0) with default parameters. Then, the k-mer^57^-based approach was selected to estimate the genome size. A total of 37.79 Gb clean reads were analyzed by Jellyfish^58^ (v.2.2.10) to generate the k-mer depth distribution with a k-mer size of 21 bp, and GenomeScope^59^ (v1.0.0) was used to estimate the genome size. Correction of Oxford Nanopore Technologies long reads and de novo assembly were performed by NextDenovo (v.2.1) with a seed cutoff of 19 kb and a read length cutoff of 1 kb. After finishing the preassembly, iterative polishing was conducted using NextPolish^23^ (v.1.1). For genome polishing, Oxford Nanopore Technologies reads and Illumina sequencing reads were subjected to three rounds of genome correction. A subprogram of Purge Haplotigs^60^ was used to generate the final contig-level assembly to retain only one copy of each of the contigs from heterozygous regions. The completeness of the genome assembly was further assessed by BUSCO^24^ (v.3) with the Embryophyta_odb9 database.

For the Hi-C sequence data, we also initially filtered out low-quality reads by fastp^56^ (v.0.20.0) with default parameters. Then, HiCUP^61^ was applied to screen valid read pairs mapped uniquely to the primary assembly for further analysis. ALLHiC^62^ (v0.8.12) was used in simple diploid mode to scaffold the genome and optimize the ordering and orientation of each clustered group, producing a chromosomal level assembly, and finally, a heatmap was plotted.

### Repeat element identification

RepeatMasker^63^ and RepeatProteinMasker^63^ were used to identify repetitive elements based on homology alignments between *C. mandshurica* genome sequences and Repbase (v.16.10). We then applied the de novo approach to improve the sensitivity of our repeat identification. Briefly, RepeatModeler^64^ and LTR_Finder^65^ (v1.06) were selected to construct the repeat library, and then RepeatMasker^63^ was employed to generate the de novo predictions.

### Gene prediction

A combination of transcriptome-based, homology-based, and de novo approaches was used to accurately predict high-quality protein-coding genes. To predict genes ab initio, Augustus^66^ (v.3.2.3), GenScan, and GlimmerHMM^67^ (v.3.0.4) were employed using a model trained based on coding sequence (CDS) data from *A. thaliana*. For homology-based prediction, protein sequences from *A. thaliana*, *C. avellana*, *Ca. fangiana*, *Cas. equisetifolia*, *Carica papaya*, *F. vesca*, *Ostryopsis nobilis*, *P. persica*, and *Q. robur* were used. For transcriptome-based prediction, the nonredundant full-length transcripts from the de novo assembly were aligned to the genome to resolve gene structures using PASA. EVidenceModeler^68^ (EVM, v.1.1.1) was used to generate the final consensus set of gene models obtained using the three approaches. Functional annotation of protein-coding genes was performed by BLASTP^69^ (v.2.7.1+) (*E*-value < 1 × 10^−5^) using SwissProt^30^ and TrEMBL^30^. InterProScan^70^ (v.5.28) and Hmmer^71^ (v3.1b2) were used to annotate protein domains by searching the InterPro and Pfam databases, respectively. Gene Ontology (GO) terms for each gene were obtained from the corresponding InterPro or Pfam entry. The pathways in which each gene might be involved were assigned by BLAST against the Kyoto Encyclopedia of Genes and Genomes^30^ (KEGG) database. Moreover, the transcription factors in *C. mandshurica* and *C. avellana* were detected using iTAK^72^ and PlantTFDB^73^. To test the significance of gene number variation among different species, the 2*2 contingency table *χ*^2^ test was executed for each transcription factor gene family based on the family gene number and the species total gene number.

### Genome evolution and expansion/contraction of gene families

To investigate the evolutionary trajectory of the *C. mandshurica* genome, a total of 11 other species were selected for phylogenetic analysis: *A. thaliana*, *B. pendula*, *C. avellana*, *Ca. fangiana*, *Cas. equisetifolia*, *F. vesca*, *J. regia*, *Ostrya rehderiana*, *Ostryopsis nobilis* (unpublished genomic data), *Q. robur* and *V. vinifera*. An all-vs-all BLASTP^69^ (v2.2.26) (*E*-value cutoff: 1 × 10^−5^) was first employed to generate similarity information for all genes. Then, we identified high-quality single-copy genes by applying OrthoMCL^74^ and constructed a phylogenetic tree with this gene set using RAxML^75^ (v8.0.0). We further estimated the times of divergence between species with MCMCtree^76^ in the PAML^49^ package (v4). The divergence time between *A. thaliana* and *V. vinifera* (107–135 Mya) acquired from TimeTree (http://www.timetree.org/) was used as the calibration point. Gene family expansion and contraction were further estimated by CAFE^77^ (v4.2) using the gene cluster information and the estimated time tree. The parameter λ was estimated along each branch with the random model, and then all the gene families were classified into three types: expanded, contracted, or unchanged.

### Evolution genes identified

We selected six genomes, i.e., those of *B. pendula*, *Ca. fangiana*, *C. mandshurica*, *Ostryopsis nobilis*, and *Ostrya rehderiana* to identify orthologs for analyzing positive selection and used *Cas. equisetifolia* as outgroup. First, Sonicparanoid^78^ was used to detect orthologs among the six genomes. Next, we used the PAML^49^ v4.8 pipeline for genome-wide detection of the genes with positive selection or rapid evolution specified in the *C. mandshurica* clade as the foreground branch. Finally, PSGs and REGs were identified based on an adjusted *P*-value < 0.05.

### Synteny and dot plot generation for the analysis of chromosome evolution

For genomes with pseudochromosomes, syntenic blocks between pairs of genomes, defined as a region containing more than five collinear genes, were searched for by the MCScanX^79^ package. The results were represented visually in combination with the phylogenetic tree. Ks of the collinear orthologous gene pairs was determined using the Perl script “add_ka_and_ks_to_collinearity.pl” implemented in MCScanX^79^. CDS anchors between every possible pair of chromosomes in multiple genomes were searched for using BLASTP^69^ (*E*-value < 1 × 10^−5^). The best matches were displayed in red, and other matches were displayed in blue to help distinguish orthology from paralogy. All dot plots were drawn by WGDI (https://pypi.org/project/WGDI/).

## Supplementary information

Supplements

Supplement Table 17

## Data Availability

All the raw sequence reads (including the Nanopore long reads, NGS short reads and Hi-C reads) used in this study have been deposited at NCBI under the BioProject accession number PRJNA638027. The genome assembly file and genome annotation files (repeat annotation and gene structure annotation) are available at figshare (doi.org/10.6084/m9.figshare.12523124.v1).

## References

[1] Wu, Z. *Vegetation in China* (1995).

[2] Wang G (2018). Progress in cultivation and utilization of *Corylus* L. Resources in China (I)—*Corylus* germplasm resources. For. Res..

[3] Ji JM, Ge ZF, Feng YS, Wang XD (2019). Lipid characterization of Chinese Wild Hazelnuts (*Corylus mandshurica* Maxim.). J. Oleo Sci..

[4] Alasalvar C, Amaral JS, Shahidi F (2006). Functional lipid characteristics of Turkish Tombul hazelnut (*Corylus avellana* L.). J. Agr. Food Chem..

[5] Tufekci F, Karatas S (2018). Determination of geographical origin Turkish hazelnuts according to fatty acid composition. Food Sci. Nutr..

[6] Teres S (2008). Oleic acid content is responsible for the reduction in blood pressure induced by olive oil. Proc. Natl Acad. Sci. USA.

[7] Cerain, L. D. & Adela. in *Mutagenic Act. Meat Samples Deep-Fry Olive Oil* Vol. 106, 989–996 (2010).

[8] Perdomo L (2015). Protective role of oleic acid against cardiovascular insulin resistance and in the early and late cellular atherosclerotic process. Cardiovasc. Diabetol..

[9] Boccacci P, Botta R (2009). Investigating the origin of hazelnut (*Corylus avellana* L.) cultivars using chloroplast microsatellites. Genet. Resour. Crop Evol..

[10] Johnson KB (1996). Eastern filbert blight of European hazelnut: It’s becoming a manageable disease. Plant Dis..

[11] Bhattarai G, Mehlenbacher S, Smith DC (2015). Novel sources of resistance to Eastern Filbert blight in Hazelnut. Hortscience.

[12] Kask K (2001). Nut quality of wild European hazelnut in Estonia and attempts at hazelnut breeding. Acta Hortic..

[13] Li TD (2018). Domestication of wild tomato is accelerated by genome editing. Nat. Biotechnol..

[14] Chen, Y. Z. et al. High oleic acid content, nontransgenic allotetraploid cotton (*Gossypium hirsutum* L.) generated by knockout of GhFAD2 genes with CRISPR/Cas9 system. *Plant Biotechnol. J.* (2020). 10.1111/pbi.13507 PMC7955888 33131175

[15] Dangl JL, Jones JDG (2001). Plant pathogens and integrated defence responses to infection. Nature.

[16] Meyers BC (2003). Genome-wide analysis of NBS-LRR-encoding genes in *Arabidopsis*. Plant Cell.

[17] Clarice JC, Shawn AM, David CS (1998). Sources of resistance to Eastern Filbert Blight in Hazelnut. J. Am. Soc. Hortic. Sci..

[18] Erdogan V, Mehlenbacher SA (2000). Interspecific hybridization in hazelnut (*Corylus*). J. Am. Soc. Hortic. Sci..

[19] Robert HW (1929). Cytological studies on the Betulaceae. II. Coryolus Alnus.. Chic. J..

[20] Robert HW (1930). Cytological studies on the Betulaceae. IV. Betula, Carpinus, Ostrya, Ostryopsis. Chic. J..

[21] Chen Z (1994). Phylogeny ang phytogeography of the Betulaceae. Acta Phyytotax. Sin..

[22] Rowley, E. R. et al. A draft genome and high-density genetic map of European Hazelnut (*Corylus avellana* L.). Preprint at https://www.biorxiv.org/content/10.1101/469015v1 (2018).

[23] Hu J, Fan JP, Sun ZY, Liu SL (2020). NextPolish: a fast and efficient genome polishing tool for long-read assembly. Bioinformatics.

[24] Simao FA (2015). BUSCO: assessing genome assembly and annotation completeness with single-copy orthologs. Bioinformatics.

[25] Salojarvi J (2017). Genome sequencing and population genomic analyses provide insights into the adaptive landscape of silver birch. Nat. Genet..

[26] Yang XY (2020). A chromosome-level reference genome of the hornbeam, *Carpinus fangiana*. Sci. Data.

[27] Yang YZ (2018). Genomic effects of population collapse in a critically endangered ironwood tree *Ostrya rehderiana*. Nat. Commun..

[28] Wang ZF (2020). Hybrid speciation via inheritance of alternate alleles of parental isolating genes. Mol. Plant.

[29] Chen F (2018). The sequenced angiosperm genomes and genome databases. Front. Plant Sci..

[30] Bairoch A, Apweiler R (2000). The SWISS-PROT protein sequence database and its supplement TrEMBL in 2000. Nucleic Acids Res..

[31] Kanehisa M, Goto S (2000). KEGG: Kyoto encyclopedia of genes and genomes. Nucleic Acids Res.

[32] Xu ZS, Chen M, Li LC, Ma YZ (2011). Functions and application of the AP2/ERF transcription factor family in crop improvement. J. Integr. Plant Biol..

[33] Doherty CJ, Van Buskirk HA, Myers SJ, Thomashow MF (2009). Roles for *Arabidopsis* CAMTA transcription factors in cold-regulated gene expression and freezing tolerance. Plant Cell.

[34] Roy S (2016). Function of MYB domain transcription factors in abiotic stress and epigenetic control of stress response in plant genome. Plant Signal Behav..

[35] Toledo-Ortiz G, Huq E, Quail PH (2003). The *Arabidopsis* basic/helix-loop-helix transcription factor family. Plant Cell.

[36] Ye GF (2019). De novo genome assembly of the stress tolerant forest species *Casuarina equisetifolia* provides insight into secondary growth. Plant J..

[37] Martinez-Garcia PJ (2016). The walnut (*Juglans regia*) genome sequence reveals diversity in genes coding for the biosynthesis of non-structural polyphenols. Plant J..

[38] Plomion C (2018). Oak genome reveals facets of long lifespan. Nat. Plants.

[39] Zapata L (2016). Chromosome-level assembly of *Arabidopsis thaliana* Ler reveals the extent of translocation and inversion polymorphisms. Proc. Natl Acad. Sci. USA.

[40] Buti M (2018). The genome sequence and transcriptome of Potentilla micrantha and their comparison to *Fragaria vesca* (the woodland strawberry). Gigascience.

[41] Jaillon O (2007). The grapevine genome sequence suggests ancestral hexaploidization in major angiosperm phyla. Nature.

[42] Chen ZD, Manchester SR, Sun HY (1999). Phylogeny and evolution of the Betulaceae as inferred from DNA sequences, morphology, and paleobotany. Am. J. Bot..

[43] Chen ZD, Lu AM (2001). Phylogeny and evolution od Betulaceae. China Acad. J..

[44] Kosztarab M, Roane MK, Drake CR (1980). Reduction of Eastern Filbert Blight on *Corylus Avellana*. Phytopathology.

[45] Sathuvalli V, Mehlenbacher SA, Smith DC (2017). High-resolution genetic and physical mapping of the eastern filbert blight resistance region in ‘Jefferson’ Hazelnut (*Corylus avellana* L.). Plant Genome.

[46] Bates PD, Stymne S, Ohlrogge J (2013). Biochemical pathways in seed oil synthesis. Curr. Opin. Plant Biol..

[47] Thelen JJ, Ohlrogge JB (2002). Metabolic engineering of fatty acid biosynthesis in plants. Metab. Eng..

[48] Ohlrogge JB (1994). Design of new plant-products - engineering of fatty-acid metabolism. Plant Physiol..

[49] Nikolau BJ, Ohlrogge JB, Wurtele ES (2003). Plant biotin-containing carboxylases. Arch. Biochem. Biophys..

[50] Yang ZH (1997). PAML: a program package for phylogenetic analysis by maximum likelihood. Comput. Appl. Biosci..

[51] Alasalvar C, Shahidi F, Cadwallader KR (2003). Comparison of natural and roasted turkish tombul hazelnut (*corylus avellana* l.) volatiles and flavor by dha/gc/ms and descriptive sensory analysis. J. Agric. Food Chem..

[52] Dormann P, Voelker TA, Ohlrogge JB (1995). Cloning and expression in *Escherichia coli* of a novel Thioesterase from *Arabidopsis thaliana* specific for long-chain acyl-acyl carrier proteins. Arch. Biochem. Biophys..

[53] Roesler K (1997). Targeting of the *Arabidopsis* homomeric acetyl-coenzyme A carboxylase to plastids of rapeseeds. Plant Physiol..

[54] Kim YS (2017). CAMTA-mediated regulation of salicylic acid immunity pathway genes in *Arabidopsis* exposed to low temperature and pathogen infection. Plant Cell.

[55] Wang ZY, Wang XY (2020). Evolutionary genomics model chromosome number reduction B chromosome production. Sci. Sin. Vitae.

[56] Chen SF, Zhou YQ, Chen YR, Gu J (2018). fastp: an ultra-fast all-in-one FASTQ preprocessor. Bioinformatics.

[57] Li RQ (2010). The sequence and de novo assembly of the giant panda genome. Nature.

[58] Marcais G, Kingsford C (2011). A fast, lock-free approach for efficient parallel counting of occurrences of k-mers. Bioinformatics.

[59] Vurture GW (2017). GenomeScope: fast reference-free genome profiling from short reads. Bioinformatics.

[60] Roach MJ, Schmidt SA, Borneman AR (2018). Purge Haplotigs: allelic contig reassignment for third-gen diploid genome assemblies. BMC Bioinformatics.

[61] Connell LW, Sexton FW, Prinja AK (1995). Further development of the heavy ion cross section for single event UPset: model (HICUP). IEEE Trans. Nucl. Sci..

[62] Zhang XT (2019). Assembly of allele-aware, chromosomal-scale autopolyploid genomes based on Hi-C data. Nat. Plants.

[63] Chen, N. in *Using RepeatMasker to Identify Repetitive Elements in Genomic Sequences* Ch. 4 (ed. Andreas, D. B.) (2004). 10.1002/0471250953.bi0410s05 18428725

[64] Bao WD, Kojima KK, Kohany O (2015). Repbase Update, a database of repetitive elements in eukaryotic genomes. Mob. DNA-Uk.

[65] Xu Z, Wang H (2007). LTR_FINDER: an efficient tool for the prediction of full-length LTR retrotransposons. Nucleic Acids Res..

[66] tanke M, Morgenstern B (2005). AUGUSTUS: a web server for gene prediction in eukaryotes that allows user-defined constraints. Nucleic Acids Res..

[67] ajoros WH, Pertea M, Salzberg SL (2004). TigrScan and GlimmerHMM: two open source ab initio eukaryotic gene-finders. Bioinformatics.

[68] Haas BJ (2008). Automated eukaryotic gene structure annotation using EVidenceModeler and the program to assemble spliced alignments. Genome Biol..

[69] Altschul SF (1997). Gapped BLAST and PSI-BLAST: a new generation of protein database search programs. Nucleic Acids Res..

[70] Zdobnov EM, Apweiler R (2001). InterProScan—an integration platform for the signature-recognition methods in InterPro. Bioinformatics.

[71] Reagan RL, Bernstein RL (2000). Data mining of signaling proteins using the HMMER method: high selectivity for protein sequence homology searches. Proc. Int. Conf. Math. Eng. Tech. Med. Biol. Sci..

[72] Zheng Y (2016). iTAK: a program for genome-wide prediction and classification of plant transcription factors, transcriptional regulators, and protein kinases. Mol. Plant.

[73] Tian F (2020). PlantRegMap: charting functional regulatory maps in plants. Nucleic Acids Res..

[74] Li L, Stoeckert CJ, Roos DS (2003). OrthoMCL: Identification of ortholog groups for eukaryotic genomes. Genome Res..

[75] Stamatakis A (2014). RAxML version 8: a tool for phylogenetic analysis and post-analysis of large phylogenies. Bioinformatics.

[76] Puttick MN (2019). MCMCtreeR: functions to prepare MCMCtree analyses and visualize posterior ages on trees. Bioinformatics.

[77] De Bie T, Cristianini N, Demuth JP, Hahn MW (2006). CAFE: a computational tool for the study of gene family evolution. Bioinformatics.

[78] Salvatore C, Wataru I (2018). Sonicparanoid: fast, accurate, and easy orthology inference. Bioinformatics.

[79] Wang YP (2012). MCScanX: a toolkit for detection and evolutionary analysis of gene synteny and collinearity. Nucleic Acids Res..

