# Supplementary material for: The Corylus mandshurica genome provides insights into the evolution of Betulaceae genomes and hazelnut breeding
Source: Hortic Res. 2021 Mar 1;8:54. doi: 10.1038/s41438-021-00495-1 (PMC7917096; doi:10.1038/s41438-021-00495-1)
Supplement: Supplementary file 1 — Supplements [file 41438_2021_495_MOESM1_ESM.doc]

# Supplementary Figures

## Supplementary
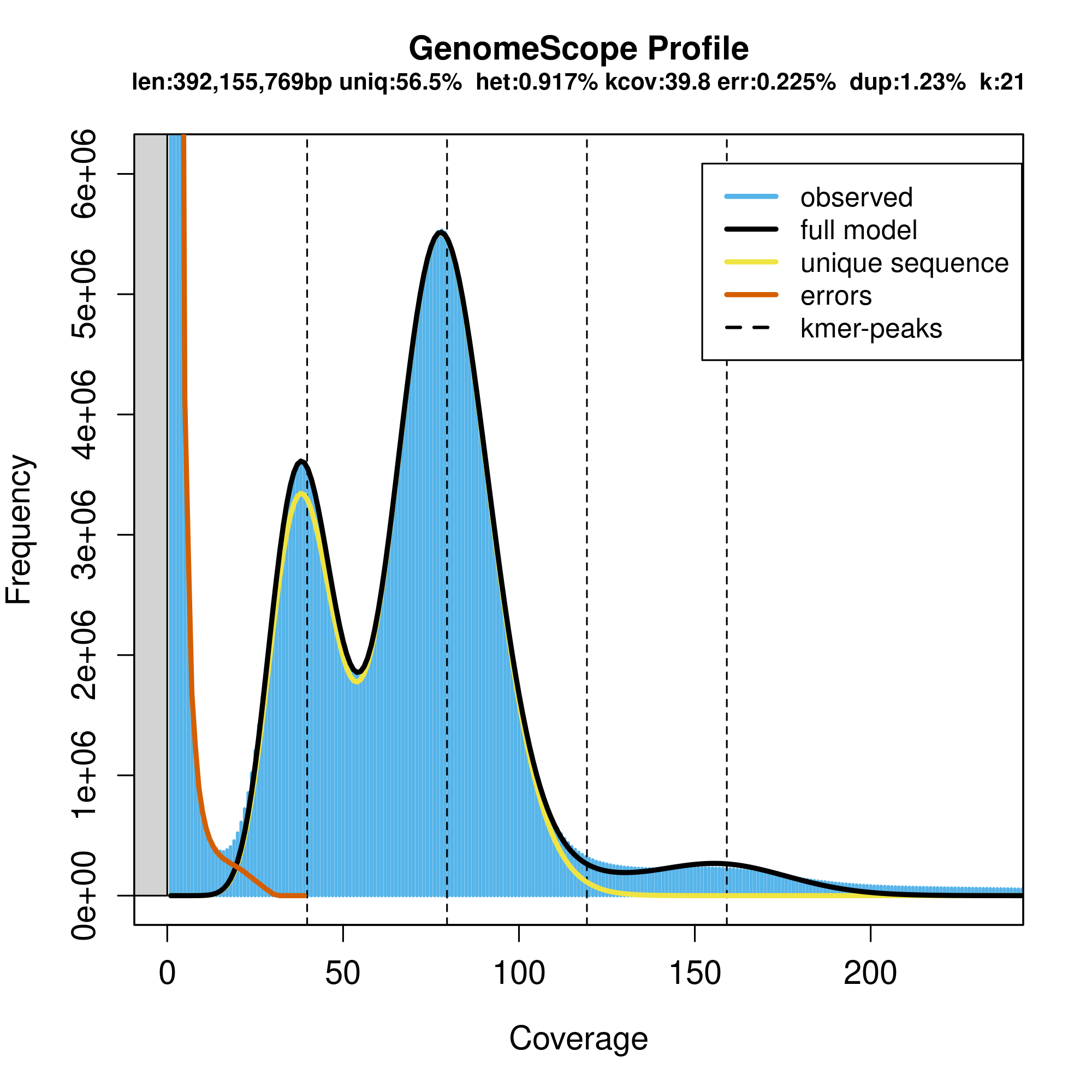
 Fig. 1. Genome size and heterozygosity estimation of *C. mandshurica* using 21 K-mer distribution.

##
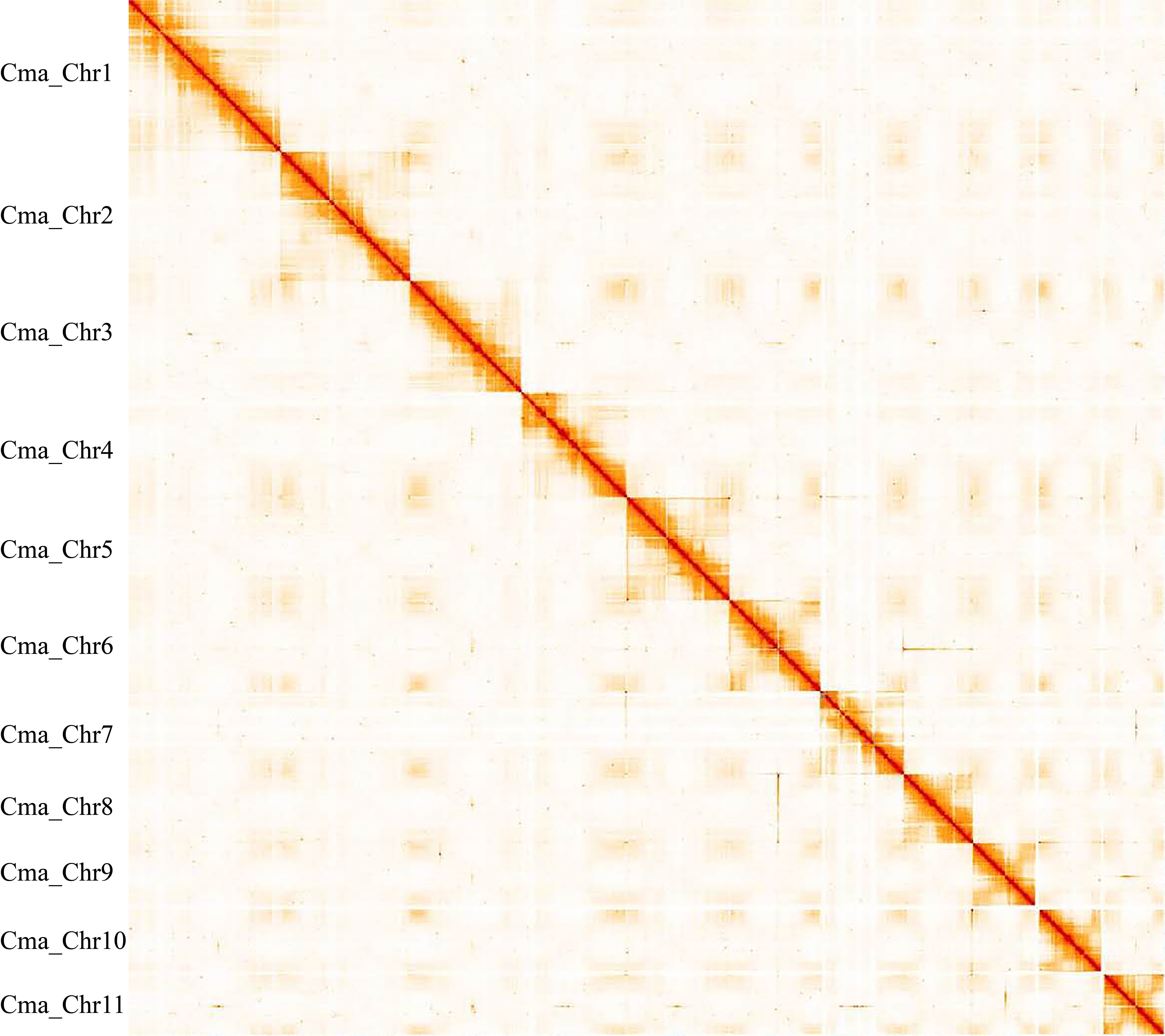


## Supplementary Fig. 2. Genome-wide analysis of chromatin interactions in *C. mandshurica* genome. Cma, *C. mandshurica.*


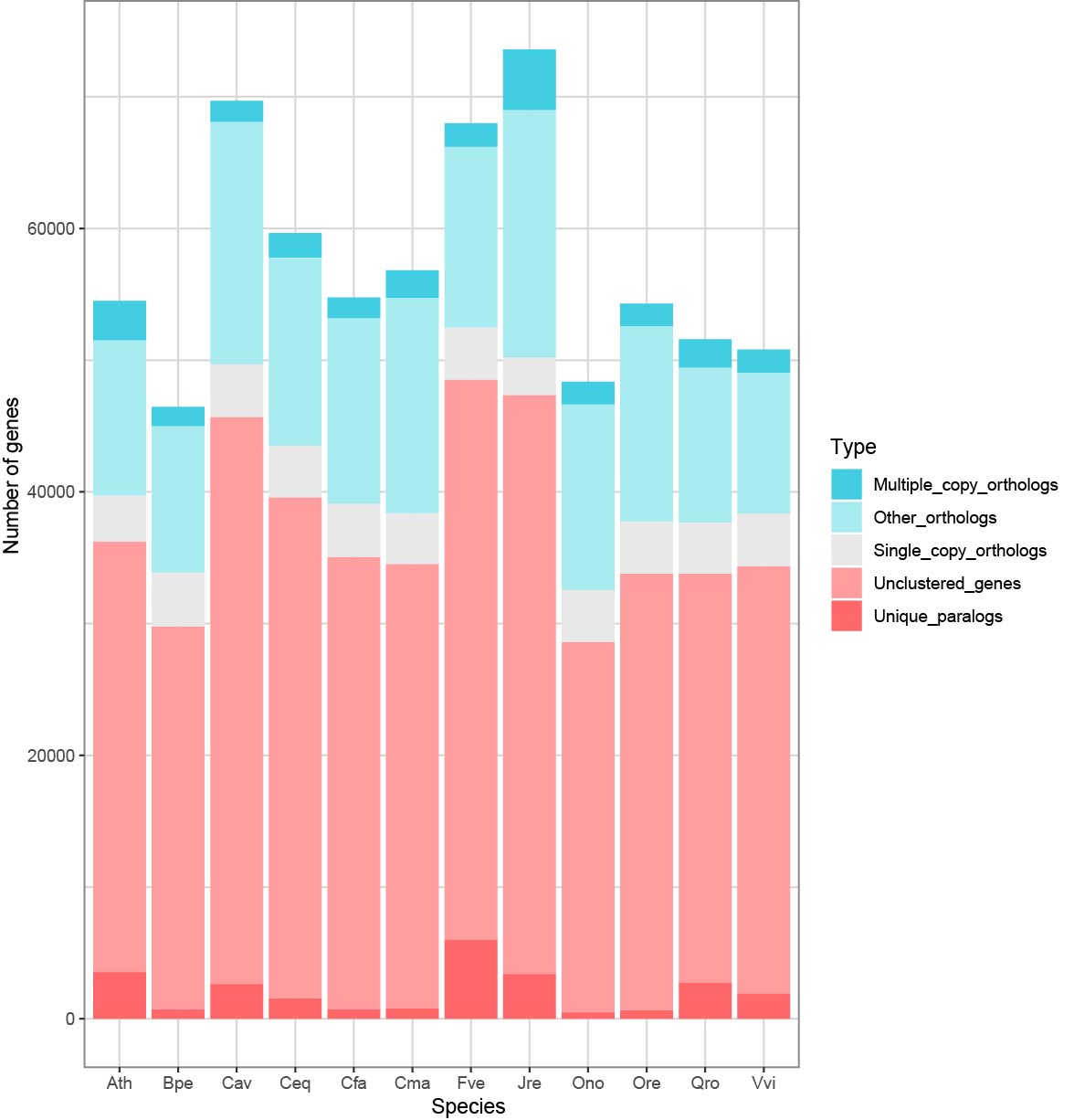


## Supplementary Fig. 3. Clusters of orthologous and paralogous gene families in *C. mandshurica* and 11 more fully sequenced plant genomes. Only the longest isoform for each gene was used. Gene families were identified using the OrthoMCL package with the default parameters. The corresponding relationship between abbreviations and full names are listing as follow: Ath: *A. thaliana*; Bpe: *B. pendula*; Cav: *C. avellana*; Ceq: *Cas. equisetifolia*; Cfa: *Ca. fangianan*; Cma: *C. mandshurica*; Fve: *F. vesca*; Jre: *J. regia*; Ono: *Ostryopsis nobilis*; Ore: *Ostrya rehderiana*; Qro: *Quercus robur*; Vvi: *Vitis vinifera*.


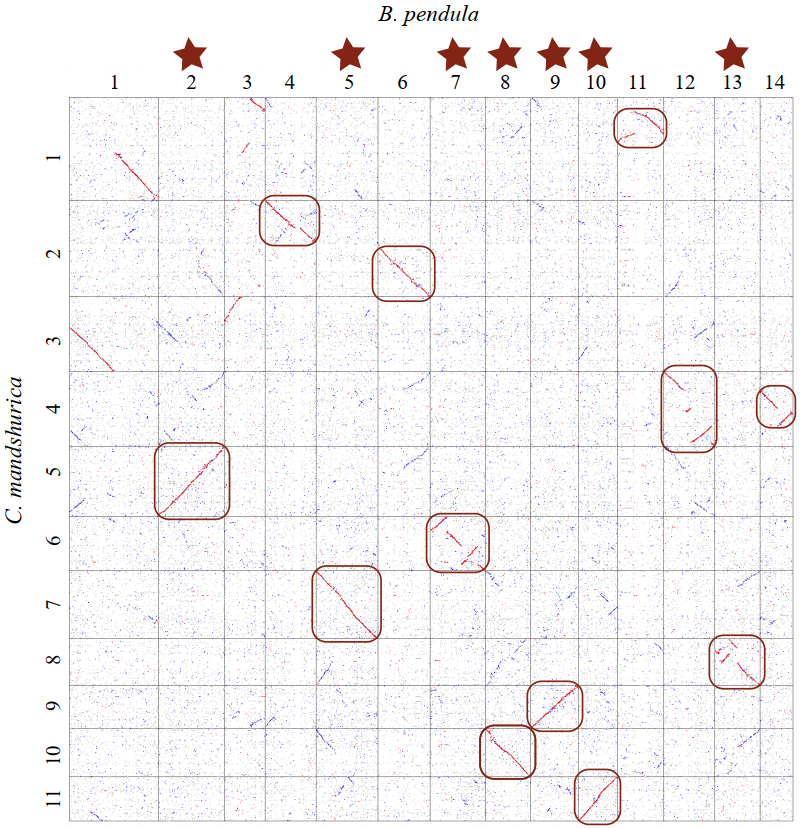


## Supplementary Fig. 4. The syntenic dotplots of *B. pendula* versus *C. mandshurica* genome. The chromosomes marked by star mean one-to-one correspondence between *B. pendula* versus *C. mandshurica.* And the framed mean the whole chromosome have been well preserved in ***C. mandshurica*** genome.

­
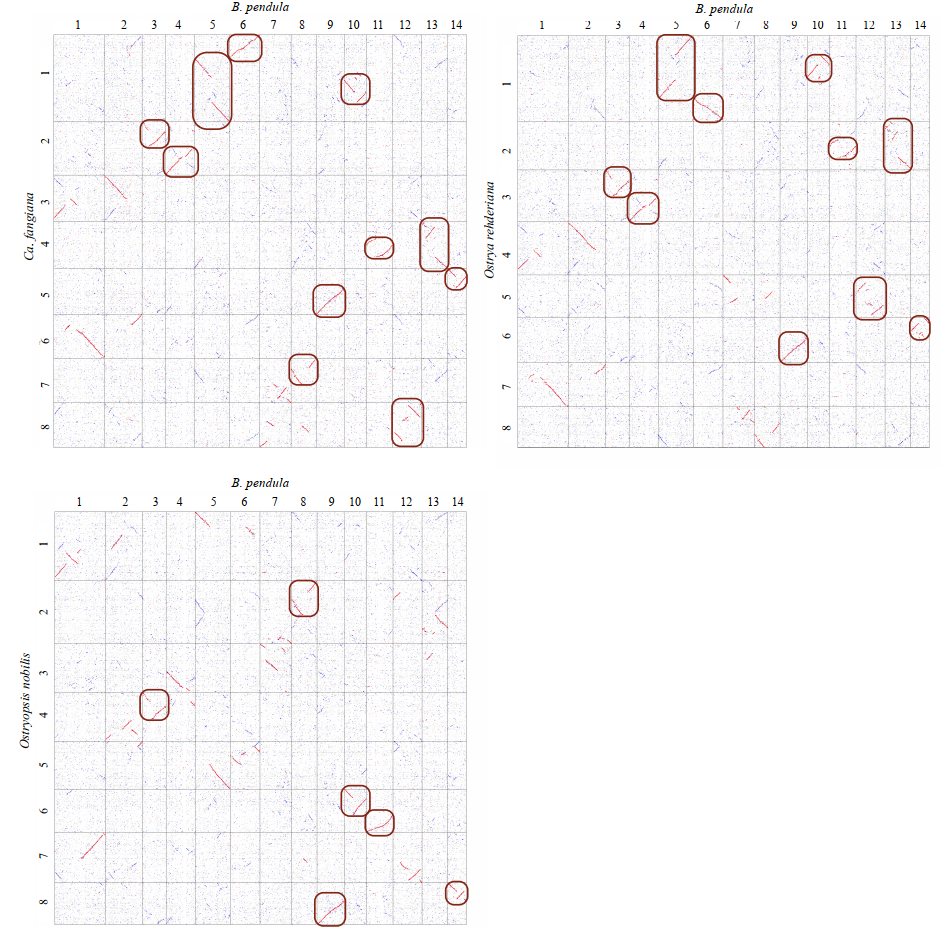


## Supplementary Fig. 5. The syntenic dotplots of *B. pendula* versus *Car. fangiana* (A)*, Ostrya* *rehderiana* (B) and *Ostryopsis nobilis*(C). The framed mean the whole chromosome have been well preserved in genome.

##
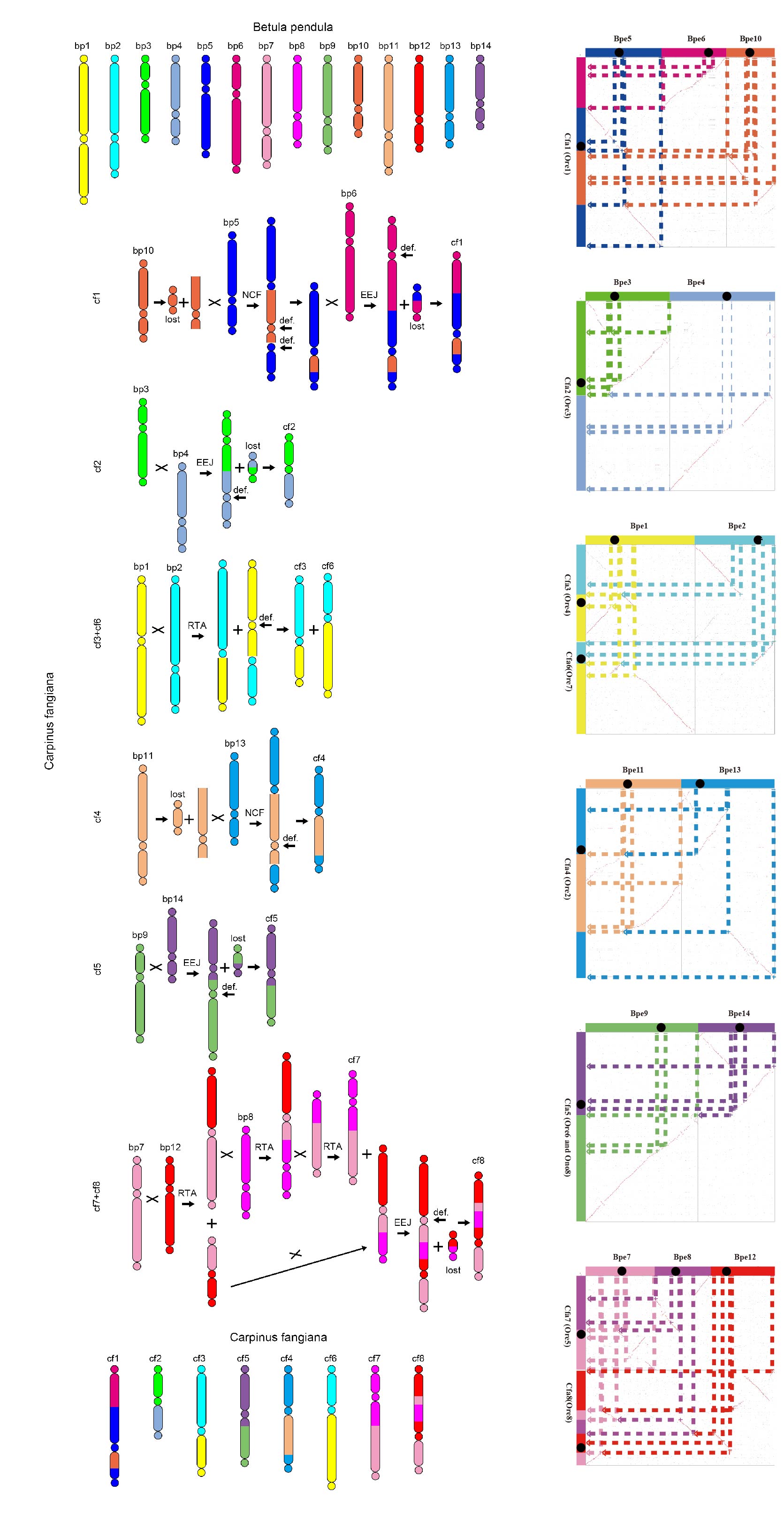
Supplementary Fig. 6. Chromosome fusions during the evolution of *Carpinus fangiana* and *Ostrya rehderiana*. Bpe: *B. pendula*; Cfa: *Ca. fangiana*; Ore: *Ostrya rehderiana*; **RTA:** reciprocally translocated chromosome arms**;** **NCF:** nested fusion of chromosome**; EEJ:** end-end joining**.**

##
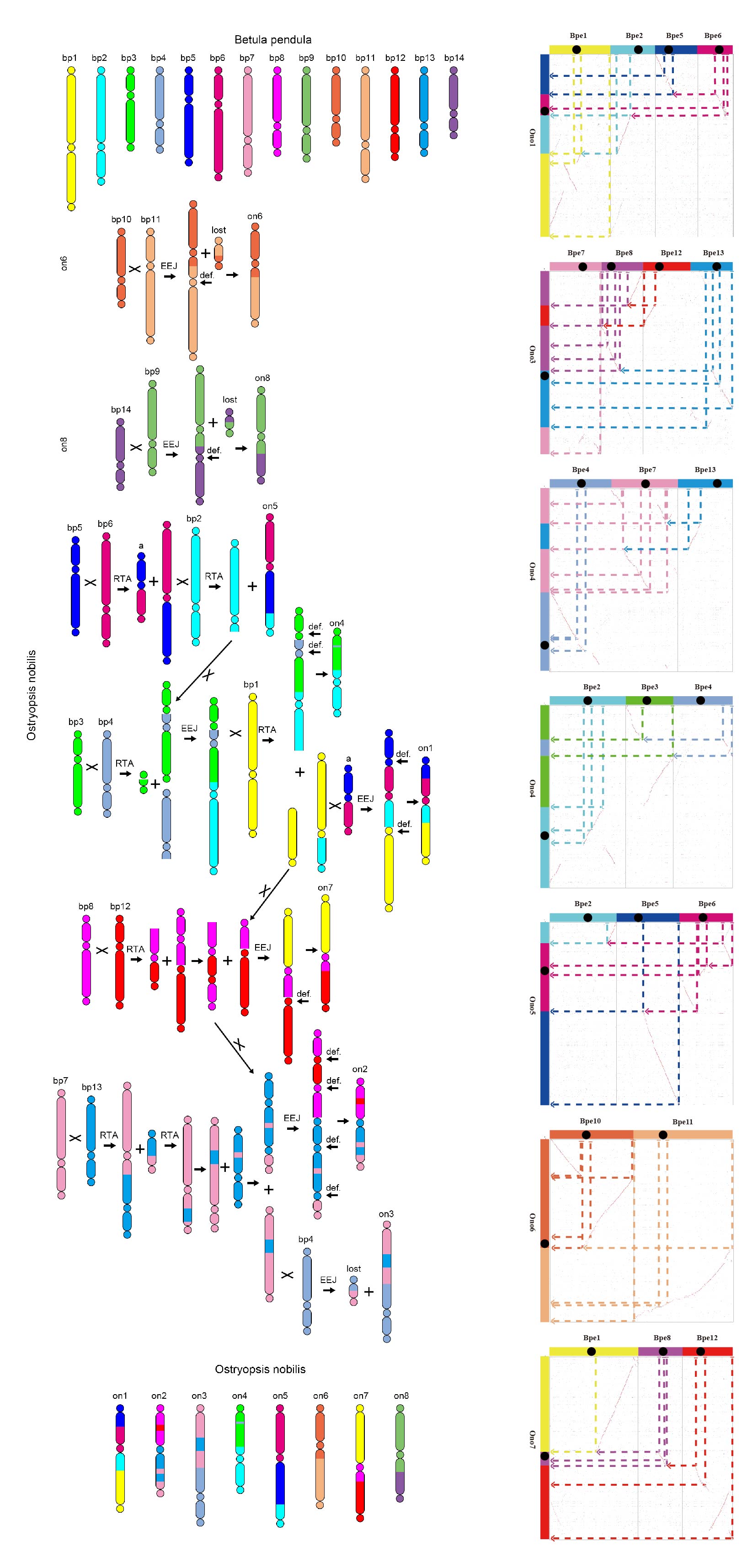
Supplementary Fig. 7. Chromosome fusions during the evolution of *Ostryopsis nobilis*. Bpe: *B. pendula*; **Ono: *Ostryopsis nobilis*; RTA:** reciprocally translocated chromosome arms**;** **NCF:** nested fusion of chromosome**; EEJ:** end-end joining**.**


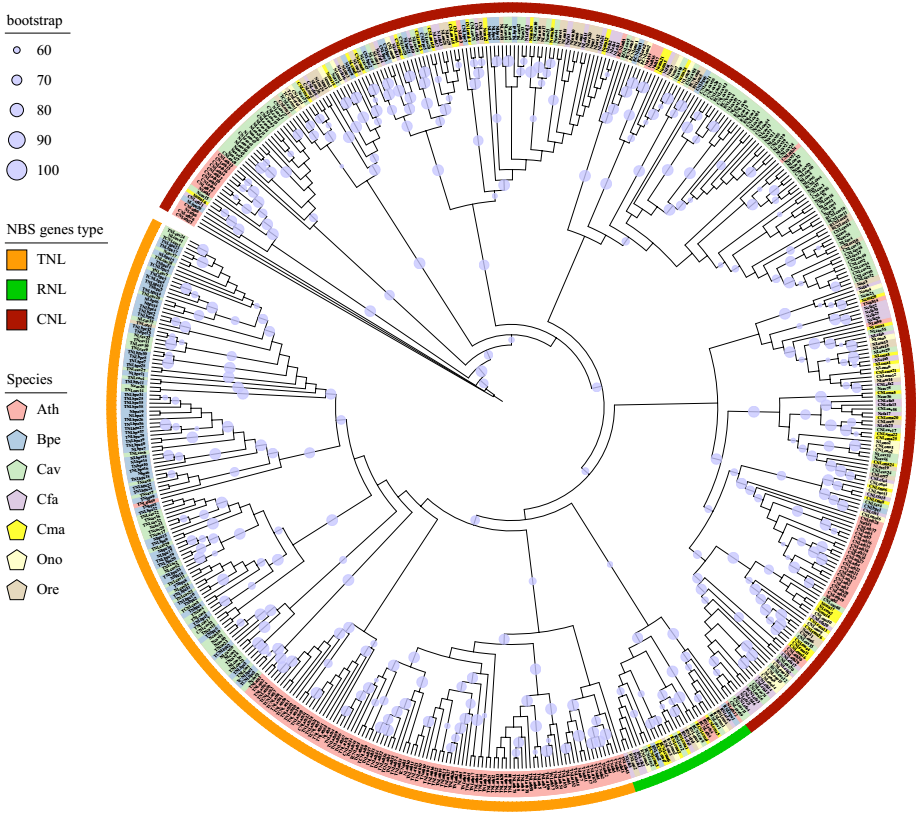


## Supplementary Fig. 8. The phylogenetic tree was constructed using putative or characterized NBS genes from 7 sequenced Betulaceae genomes and *A. thaliana*. The corresponding relationship between abbreviations and full names are listing as follow: Ath: *A. thaliana*; Bpe: *B. pendula*; Cav: *C. avellana*; Ceq: *Cas. equisetifolia*; Cfa: *Ca. fangianan*; Cma: *C. mandshurica*; Fve: *F. vesca*; Jre: *J. regia*; Ono: *Ostryopsis nobilis*; Ore: *Ostrya rehderiana*; Qro: *Quercus robur*; Vvi: *Vitis vinifera*.


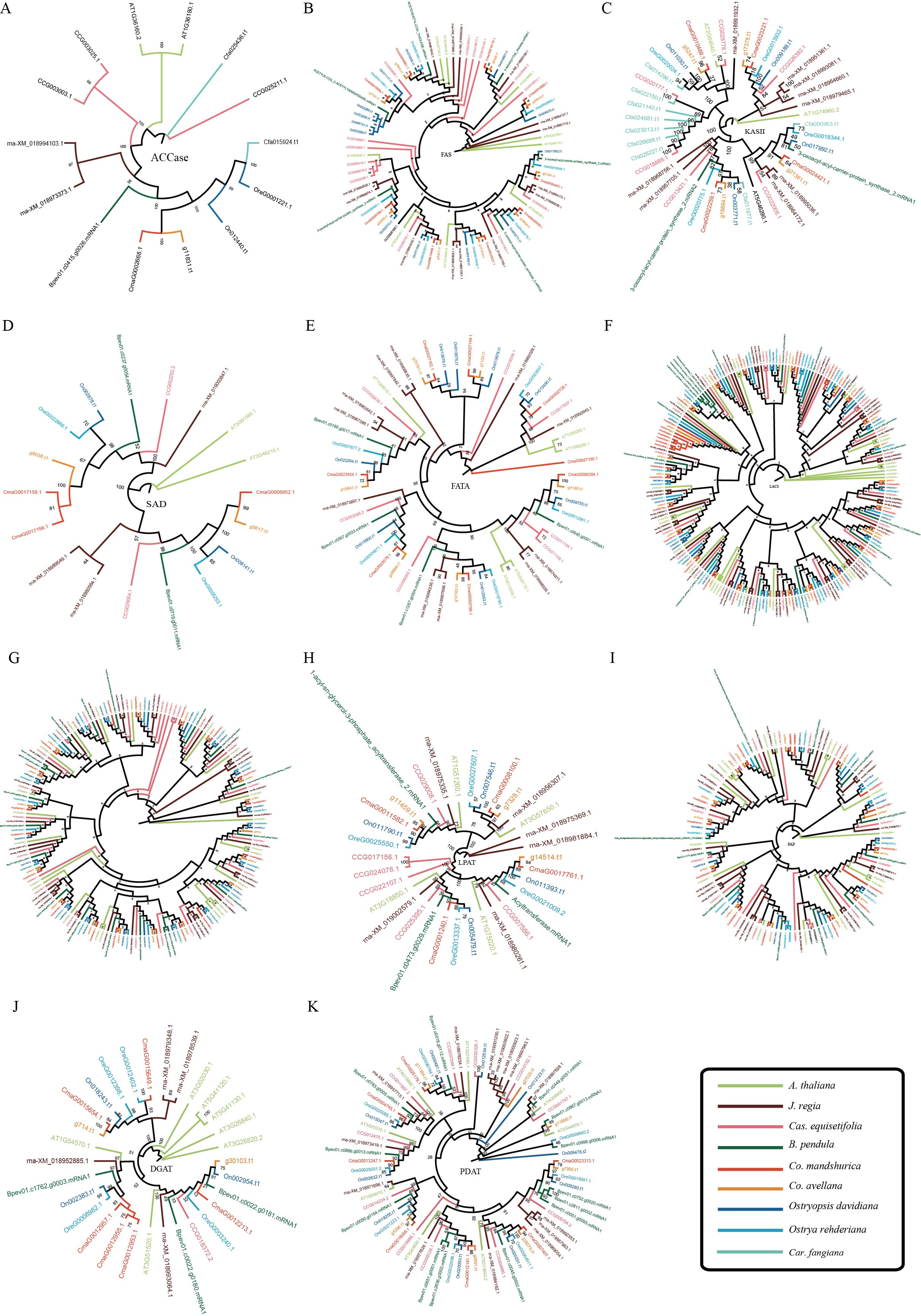


## Supplementary Fig. 9. Phylogenetic analysis of oil biosynthesis-related gene families. Different colors correspond to different species and the order of gene family name as follow: ACCase (A), FAB (B), KASII (C), SAD (D), FATA (E), LACS (F), GPAT(G), LPAT(H), PAP(I), DGAT (J) and PDCT (K).


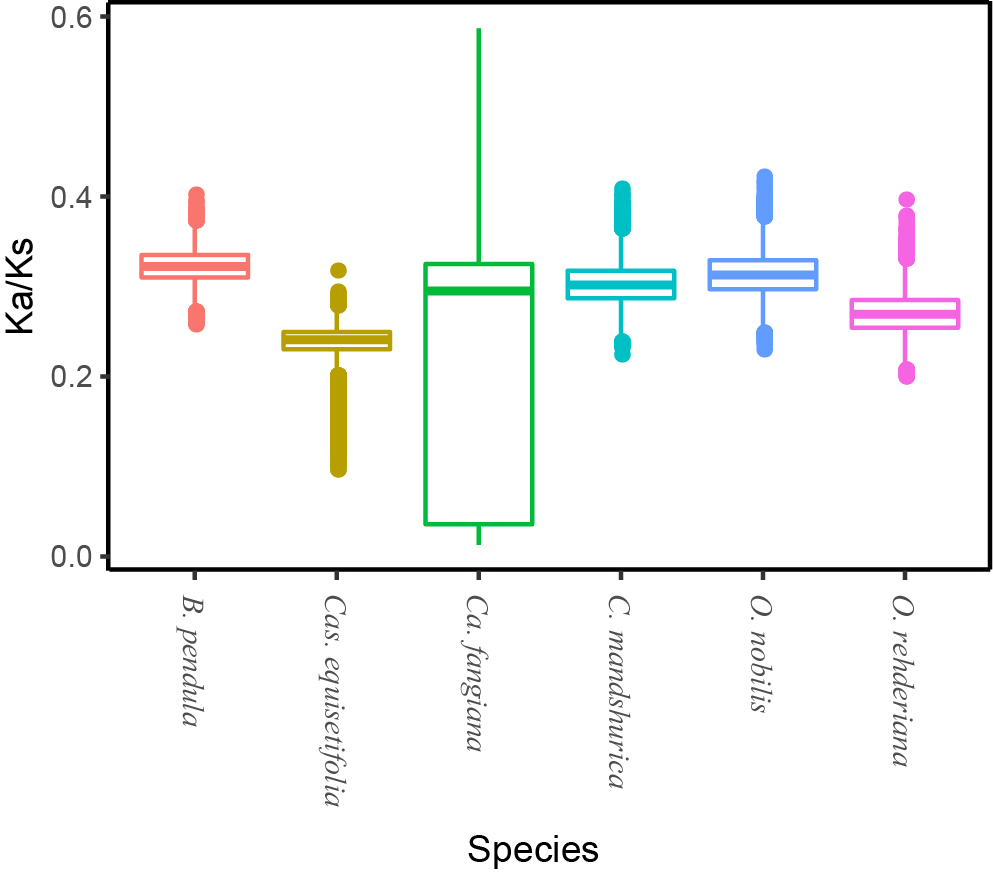


## Supplementary Fig. 10. Ka/Ks values in different Betulaceae species.


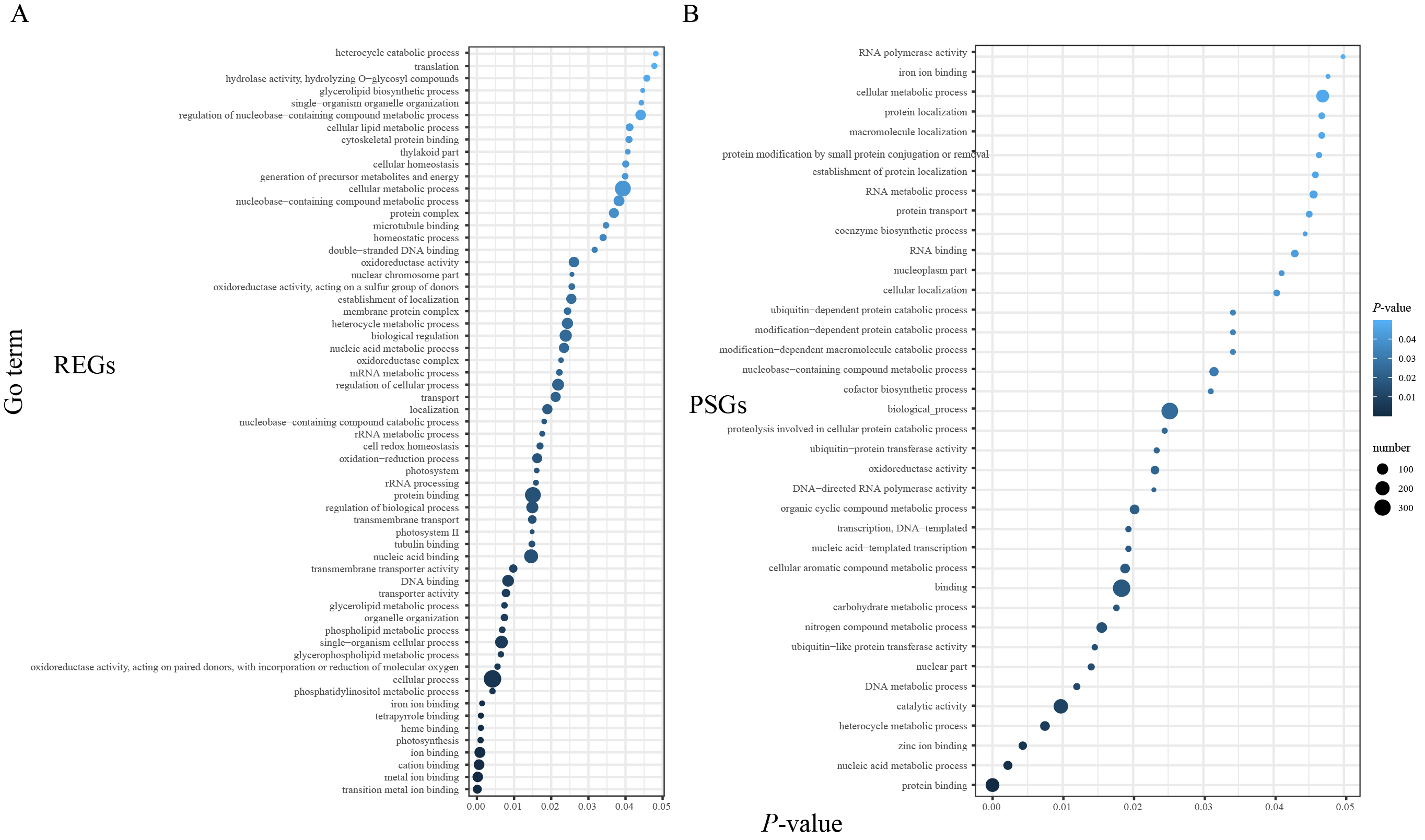


## Supplementary Fig. 11. Functional enrichment of the positive selective genes (PSGs) (A) and the rapid evolution genes (REGs) (B) *in C. mandshurica.* The gene number and the signification of each Go term was indicated by size and color of dot.

# Supplementary Tables

## Supplementary Table 1. Statistics of *C. mandshurica* genome sequencing data.

| **Items** | **ONT reads** | **NGS reads** |
| --- | --- | --- |
| Total number of reads | 2,023,675 | 258,562,360 |
| Total number of sequenced bases (bp) | 38,084,881,698 | 38,784,345,000 |
| Mean reads length (bp) | 17,819.7 | 150 |
| Max reads length (bp) | 167,817 | 150 |
| N50 (bp) | 26,715 | - |
| Coverage (X)* | 97.1 | 98.9 |

* Coverage (X) = read length × read count / estimated genome size

## Supplementary Table 2. Statistics of pre-assembly of *C. mandshurica* genome.

| **Contig number** | **Contig length (bp)** | **Contig N50 (bp)** | **Contig max (bp)** | **GC content (%)** |
| --- | --- | --- | --- | --- |
| 81 | 367,672,720 | 14,849,403 | 22,635,284 | 36.03 |

## Supplementary Table 3. Quality assessment of recent published genome.

| **Species name** | **Contig N50 (Mb)** | **Genome Size (Mb)** | **Complete BUSCO (%)** | **Published time** | **DOI** |
| --- | --- | --- | --- | --- | --- |
| *Corylus mandshurica^*^* | 14.85 | 367.7 | 97.1 | This work | This work |
| *Corylus avellana^*^* | 0.02 | 345.5 | 86.5 | 2018 | 10.1101/469015 |
| *Carpinus fangiana^*^* | 1.91 | 386.2 | 95.3 | 2020 | 10.1038/s41597-020-0370-5 |
| *Ostrya rehderiana^*^* | 2.31 | 385.9 | 97.0 | 2018 | 10.1038/s41467-018-07913-4 |
| *Betula pendula^*^* | 0.24 | 435.2 |  | 2017 | 10.1038/ng.3862 |
| *Salix suchowensis* | 0.26 | 356.5 | 94.8 | 2020 | 10.1038/s41438-020-0268-6 |
| *Cannabis sativa* | 0.51 | 807.7 | 93.0 | 2020 | 10.1038/s41438-020-0295-3 |
| *Prunus armeniaca* | 1.02 | 221.9 | 98.0 | 2019 | 10.1038/s41438-019-0215-6 |
| *Diospyros oleifera* | 0.89 | 849 | 89.9 | 2019 | 10.1038/s41438-019-0227-2 |
| *Luffa cylindrica* | 5.00 | 669 | 88.5 | 2019 | 10.1111/1755-0998.13129 |
| *Kingdonia uniflora* | 2.10 | 1,004 | 90.6 | 2020 | 10.1016/j.isci.2020.101124 |
| *Chenopodium pallidicaule* | 0.52 | 363 | 97.5 | 2020 | 10.1002/aps3.11300 |
| *Paeonia suffruticosa* | 0.07 | 11,800 | 61.2 | 2020 | 10.1002/ece3.5965 |
| *Kandelia obovata* | 5.74 | 178 | 97.3 | 2020 | 10.1038/s41438-020-0300-x |
| *Gossypium hirsutum* | 0.78 | 2,305 | 97.0 | 2020 | 10.1038/s41588-020-0614-5 |
| *Gossypium barbadense* | 1.80 | 2,196 | 97.0 | 2020 | 10.1038/s41588-020-0614-5 |
| *Gossypium mustelinum* | 2.30 | 2,315 | 97.0 | 2020 | 10.1038/s41588-020-0614-5 |
| *Gossypium tomentosum* | 10.00 | 2,194 | 97.0 | 2020 | 10.1038/s41588-020-0614-5 |
| *Gossypium darwinii* | 9.10 | 2,183 | 97.0 | 2020 | 10.1038/s41588-020-0614-5 |
| *Lonicera japonica* | 2.10 | 843 | 94.7 | 2020 | 10.1111/nph.16552 |
| *Populus ilicifolia* | 0.06 | 396 | 94.0 | 2020 | 10.1111/tpj.14744 |
| *Simmondsia chinensis* | 5.20 | 887 | 93.5 | 2020 | 10.1126/sciadv.aay3240 |
| *Eriobotrya japonica* | 5.02 | 760 | 96.8 | 2020 | 10.1093/gigascience/giaa015 |
| *Mangifera indica* | 3.50 | 393 | 95.9 | 2020 | 10.1186/s13059-020-01959-8 |
| *Mikania micrantha* | 1.35 | 1,790 | 91.0 | 2020 | 10.1038/s41467-019-13926-4 |

* indicates the species belong to the Betulaceae.

## Supplementary Table 4. Numbers of Illumina short reads mapped to the assembled *C. mandshurica* genome.

| **File** | **Total_pairs** | **Pair_end_mapped_reads** | **Pair_end_mapped_ratio** | **Single_end_mapped_reads** | **Single_end_mapped_ratio** | **Total_mapped_reads** | **Total_mapped_ratio** |
| --- | --- | --- | --- | --- | --- | --- | --- |
| genome | 257208116 | 249534634 | 97.02 | 608515 | 0.24 | 254264643 | 98.86 |
| Leaf | 53509773 | 42051368 | 78.59 | 40988 | 0.08 | 53338185 | 99.68 |

## Supplementary Table 5. Evaluation of the *C. mandshurica* genome completeness using data set of RNA transcripts.

| **Dataset** | **Length type Number** | **Number** | **Total length Number** | | **Covered by assembly** | | **With > 90% sequence in one scaffold** | **With > 50% sequence in one scaffold** |
| --- | --- | --- | --- | --- | --- | --- | --- | --- |
|  |  |  | **Number** | **Percentage** | **Number** | **Percentage** |  |  |
| Leaf | >200bp | 65993 | 41603353 | 96.83 | 58442 | 0.885578774 | 63418 | 97.00 |
|  | >500bp | 23548 | 29315771 | 99.71 | 21660 | 0.91982334 | 23349 | 99.16 |
|  | >1000bp | 12179 | 21237572 | 99.91 | 11204 | 0.919944166 | 12102 | 99.37 |

## Supplementary Table 6. Statistics of Hi-C data and assessment.

| **Statistics of Hi-C data** | | | |
| --- | --- | --- | --- |
| Number of clean reads | Number of bases (bp) | Clean reads rate (%) | % ≥ Q30 |
| 290,671,288 | 42,037,446,200 | 96.98 | 94.98 |
| **Statistics of mapping** | | | |
| Mapping type | | Number of reads | Ratio (%) |
| Total read pairs | | 290,671,288 | 100 |
| Mapped reads | | 536,897,669 | 92.3 |
| Unique mapped read pairs | | 146,648,209 | 50.5 |
| **Statistics of valid Hi-C data** | | | |
| Type | | Number of reads | Ratio (%) |
| Unique paired alignments | | 146,648,209 | 100 |
| Valid interaction pairs | | 127,703,957 | 87.1 |
| Dangling end pairs | | 3,373,084 | 2.3 |
| Re-ligation pairs | | 4,008,919 | 2.7 |
| Self-cycle pairs | | 613,035 | 0.4 |
| Dumped pairs | | 10,949,214 | 7.5 |

## Supplementary Table 7. Summary of chromosome level assembly based on Hi-C data.

| **chromosome** | **Number of anchored**  **and oriented contigs** | **Length of anchored**  **and oriented contigs (bp)** | **Number of anchored**  **and oriented gene** | **Gap base (N)** | **N (%)** |
| --- | --- | --- | --- | --- | --- |
| Cma_Chr1 | 13 | 52,982,101 | 4,052 | 1,202 | 0.002 |
| Cma_Chr2 | 9 | 48,184,857 | 3,782 | 807 | 0.002 |
| Cma_Chr3 | 5 | 38,176,155 | 2,932 | 401 | 0.001 |
| Cma_Chr4 | 8 | 36,781,874 | 2,955 | 703 | 0.002 |
| Cma_Chr5 | 13 | 36,270,330 | 2,746 | 1202 | 0.003 |
| Cma_Chr6 | 9 | 31,747,834 | 2,107 | 803 | 0.003 |
| Cma_Chr7 | 4 | 31,697,389 | 2,688 | 302 | 0.001 |
| Cma_Chr8 | 6 | 23,849,844 | 1,833 | 504 | 0.002 |
| Cma_Chr9 | 7 | 23,215,594 | 1,704 | 601 | 0.003 |
| Cma_Chr10 | 4 | 22,830,127 | 1,882 | 301 | 0.001 |
| Cma_Chr11 | 3 | 21,943,615 | 1,728 | 202 | 0.001 |
| Total | 81 | 367,672,720 | 28,409 | 7,028 | 0.002 |

## Supplementary Table 8. Summary statistics of the annotated transposable elements in the *C. mandshurica* genome.

| **Type** | **Length(bp)** | **%_of_repeat** | **%_of_genome** |
| --- | --- | --- | --- |
| **SINE** | **126516** | **0.05** | **0.03** |
| **LINE** | **19947416** | **7.89** | **5.43** |
| L1 | 19546768 | 7.73 | 5.32 |
| L2 | 161836 | 0.06 | 0.04 |
| **LTR** | **212954355** | **84.26** | **57.92** |
| Copia | 62421172 | 24.70 | 16.98 |
| Gypsy | 64205574 | 25.40 | 17.46 |
| **DNA** | **16142453** | **6.39** | **4.39** |
| CMC-EnSpm | 5274077.00 | 2.09 | 1.43 |
| MuDR | 3602498.00 | 1.43 | 0.98 |
| PIF-Harbinger | 1083648.00 | 0.43 | 0.29 |
| hAT-Ac | 3764082.00 | 1.49 | 1.02 |
| hAT-Tip100 | 1892942.00 | 0.75 | 0.51 |
| **Unclassified** | **28429483** | **11.24** | **7.73** |
| Unclassified_Other/Composite | 426 | >0.01 | >0.01 |
| Unclassified_RC | 19870 | >0.01 | >0.01 |
| Unclassified_RC/Helitron | 3310549 | 1.31 | 0.90 |
| Unclassified_RC/Helitron-2 | 783 | >0.01 | >0.01 |
| Unclassified_Retroposon/SVA | 73 | 0.00 | 0.00 |
| Unknown | 25097782 | 9.93 | 6.83 |
| **Satellite** | **155769** | **0.06** | **0.04** |
| **Simple repeat** | **19227345** | **7.61** | **5.23** |
| **Small RNA** | **41243** | **0.02** | **0.01** |
| **Total** | **252743315** | **100** | **68.74** |
| **Low complexity** | **801354** | **-** | **0.22** |

## Supplementary Table 9. Prediction of protein-coding genes in the *C. mandshurica* genomes.

| **Gene set** | | **Total Genes Predicted** | **Average Gene Length (bp)** | **Average CDS Length (bp)** | **Average Exons per Gene** | **Average Exon Length (bp)** | **Average Intron Length (bp)** |
| --- | --- | --- | --- | --- | --- | --- | --- |
| *De novo* | augustus | 54206 | 3865.71 | 1364.84 | 5.17 | 263.77 | 348.47 |
|  | Glimmerhmm | 23574 | 9976.86 | 1325.09 | 6.40 | 207.02 | 1601.94 |
|  | Genemark | 30814 | 1976.20 | 818.68 | 3.29 | 248.66 | 504.95 |
| Homolog | *Arabidopsis thaliana* | 21611 | 4292.59 | 1323.86 | 5.55 | 238.43 | 652.13 |
|  | *Corylus avellana* | 42277 | 2251.21 | 941.55 | 3.57 | 263.62 | 509.28 |
|  | *Carpinus fangiana* | 20905 | 3564.23 | 1180.02 | 4.62 | 255.19 | 657.87 |
|  | *Casuarina equisetifolia* | 23503 | 4071.05 | 1292.39 | 5.20 | 248.75 | 662.31 |
|  | *Carica papaya* | 20734 | 3938.76 | 1256.80 | 5.21 | 241.03 | 636.39 |
|  | *Fragaria vesca* | 26331 | 4030.81 | 1338.73 | 5.06 | 264.34 | 662.35 |
|  | *Ostryopsis nobilis* | 25756 | 3825.72 | 1153.32 | 4.99 | 231.27 | 670.29 |
|  | *Prunus persica* | 24384 | 4170.22 | 1332.08 | 5.33 | 249.86 | 655.27 |
|  | *Quercus robur* | 22555 | 3462.90 | 1214.34 | 4.59 | 264.67 | 626.65 |
| RNA-seq | | 31571 | 5564.35 | 989.38 | 4.77 | 207.44 | 934.53 |
| EVM | | 28409 | 3561.82 | 1154.44 | 5.00 | 230.89 | 601.85 |

## Supplementary Table 10 Quality assessment of the gene prediction genome of *C. mandshurica* using BUSCOs.

| **Type** | **Number** | **Percent (%)** |
| --- | --- | --- |
| Complete BUSCOs (C) | 1327 | 92.2 |
| Complete and single-copy BUSCOs (S) | 1273 | 88.4 |
| Complete and duplicated BUSCOs (D) | 54 | 3.8 |
| Fragmented BUSCOs (F) | 55 | 3.8 |
| Missing BUSCOs (M) | 58 | 4.0 |
| Total BUSCO groups searched | 1440 | 100 |

## Supplementary Table 11. Functional annotation of predicted protein-coding genes in the *C. mandshurica* genome

| **Database** | **Number of genes annotated** | **Percentage (%)** |
| --- | --- | --- |
| GO | 15894 | 55.95 |
| KEGG | 5287 | 28.41 |
| SWISS-PROT | 18864 | 66.4 |
| InterPro | 26,309 | 92.6 |
| TrEMBL | 23,665 | 83.3 |
| Total | 25,923 | 92.61 |

## Supplementary Table 12. Comparation of transcription factors between the *C. mandshurica* and *C. avellana* genome.

| **Transcription Factor** | ***C. mandshurica*** | ***C. avellana*** | ***p*-values** | **Transcription Factor** | ***C. mandshurica*** | ***C. avellana*** | ***p*-values** |
| --- | --- | --- | --- | --- | --- | --- | --- |
| AP2/ERF-ERF | 139 | 98 | 0.00003 | HB-other | 18 | 15 | 0.34867 |
| MYB | 120 | 94 | 0.00134 | CPP | 8 | 5 | 0.35434 |
| bHLH | 122 | 96 | 0.00135 | HD-ZIP | 31 | 29 | 0.35681 |
| CAMTA | 13 | 3 | 0.00758 | ARR-B | 11 | 8 | 0.36449 |
| HB-KNOX | 9 | 1 | 0.01083 | B3 | 47 | 47 | 0.37501 |
| Others | 68 | 54 | 0.02087 | HB-PHD | 2 | 0 | 0.39233 |
| M-type_MADS | 16 | 39 | 0.02626 | Rcd1-like | 2 | 0 | 0.39233 |
| GeBP | 16 | 7 | 0.03025 | OFP | 19 | 17 | 0.43487 |
| GNAT | 28 | 18 | 0.04269 | TALE | 17 | 15 | 0.44965 |
| C2H2 | 101 | 92 | 0.04589 | HB-HD-ZIP | 27 | 26 | 0.45677 |
| MADS-M-type | 18 | 39 | 0.05879 | Trihelix | 27 | 26 | 0.45677 |
| GRAS | 68 | 59 | 0.06231 | mTERF | 27 | 41 | 0.45883 |
| SET | 36 | 27 | 0.06817 | SNF2 | 32 | 32 | 0.48888 |
| AP2/ERF-AP2 | 14 | 7 | 0.07435 | ARID | 11 | 9 | 0.49524 |
| CO-like | 10 | 4 | 0.08440 | Nin-like | 11 | 9 | 0.49524 |
| MYB-related | 71 | 64 | 0.08812 | RWP-RK | 11 | 9 | 0.49524 |
| GARP-G2-like | 33 | 25 | 0.08877 | ZF-HD | 10 | 8 | 0.50241 |
| C2C2-GATA | 21 | 14 | 0.10438 | zf-HD | 10 | 8 | 0.50241 |
| GATA | 21 | 14 | 0.10438 | BES1 | 8 | 6 | 0.51491 |
| bZIP | 52 | 45 | 0.10560 | C2C2-YABBY | 5 | 3 | 0.51919 |
| C2C2-CO-like | 9 | 4 | 0.13788 | YABBY | 5 | 3 | 0.51919 |
| AP2 | 19 | 13 | 0.14249 | MIKC_MADS | 7 | 5 | 0.51934 |
| Jumonji | 19 | 13 | 0.14249 | SRS | 6 | 4 | 0.52139 |
| WRKY | 59 | 54 | 0.14320 | G2-like | 36 | 37 | 0.52343 |
| C3H | 55 | 50 | 0.15014 | TRAF | 19 | 18 | 0.53404 |
| AUX/IAA | 25 | 19 | 0.15114 | FAR1 | 26 | 38 | 0.57307 |
| E2F/DP | 6 | 2 | 0.17531 | ARF | 9 | 15 | 0.59979 |
| E2F-DP | 6 | 2 | 0.17531 | WOX | 12 | 11 | 0.62389 |
| LIM | 6 | 2 | 0.17531 | HB-WOX | 11 | 10 | 0.63939 |
| MYB_related | 67 | 64 | 0.17873 | SBP | 19 | 19 | 0.64037 |
| HSF | 9 | 19 | 0.24274 | NF-YC | 10 | 9 | 0.65582 |
| GARP-ARR-B | 10 | 6 | 0.24491 | GRF | 9 | 8 | 0.67333 |
| PLATZ | 10 | 6 | 0.24491 | TUB | 9 | 8 | 0.67333 |
| C2C2-Dof | 26 | 22 | 0.25290 | NAC | 84 | 96 | 0.69106 |
| Dof | 26 | 22 | 0.25290 | IWS1 | 8 | 7 | 0.69209 |
| NF-YB | 15 | 11 | 0.26587 | Tify | 8 | 7 | 0.69209 |
| SWI/SNF-BAF60b | 15 | 11 | 0.26587 | NF-YA | 7 | 6 | 0.71237 |
| TCP | 10 | 20 | 0.27519 | LBD | 26 | 36 | 0.73135 |
| Pseudo | 4 | 3 | 0.78674 | EIL | 4 | 4 | 1.00000 |
| HMG | 9 | 9 | 0.84390 | HB-BELL | 8 | 9 | 1.00000 |
| C2C2-LSD | 2 | 1 | 0.85941 | HRT | 1 | 1 | 1.00000 |
| LSD | 2 | 1 | 0.85941 | HRT-like | 1 | 1 | 1.00000 |
| BSD | 1 | 0 | 0.91855 | LFY | 1 | 1 | 1.00000 |
| S1Fa-like | 1 | 0 | 0.91855 | MBF1 | 2 | 2 | 1.00000 |
| STAT | 1 | 0 | 0.91855 | MED6 | 1 | 1 | 1.00000 |
| ULT | 1 | 0 | 0.91855 | MED7 | 1 | 1 | 1.00000 |
| B3-ARF | 11 | 15 | 0.94435 | NF-X1 | 2 | 2 | 1.00000 |
| DDT | 6 | 6 | 0.94892 | NOZZLE | 1 | 1 | 1.00000 |
| Alfin-like | 5 | 5 | 0.99561 | NZZ/SPL | 1 | 1 | 1.00000 |
| DBB | 5 | 5 | 0.99561 | RAV | 4 | 4 | 1.00000 |
| MADS-MIKC | 5 | 5 | 0.99561 | RB | 1 | 1 | 1.00000 |
| AP2/ERF-RAV | 4 | 4 | 1.00000 | SAP | 1 | 1 | 1.00000 |
| BBR-BPC | 4 | 4 | 1.00000 | SOH1 | 1 | 1 | 1.00000 |
| Coactivator | 3 | 3 | 1.00000 | SWI/SNF-SWI3 | 4 | 4 | 1.00000 |
| CSD | 2 | 3 | 1.00000 | TAZ | 5 | 6 | 1.00000 |
| DBP | 2 | 2 | 1.00000 | VOZ | 2 | 2 | 1.00000 |
| LUG | 5 | 4 | 0.75902 | Whirly | 2 | 2 | 1.00000 |
| PHD | 31 | 28 | 0.29522 | LOB | 26 | 36 | 0.73135 |

## Supplementary Table 13. Prediction of noncoding RNAs in the *C. mandshurica* genomes.

|  | **Copy number** | **Average length(bp)** | **Total length(bp)** | **ratio of genome** |
| --- | --- | --- | --- | --- |
| miRNA | 83 | 120.28 | 9983 | 0.003% |
| rRNA | 336 | 128.10 | 43043 | 0.012% |
| snRNA | 329 | 115.34 | 37947 | 0.010% |
| tRNA | 195 | 75.11 | 14647 | 0.004% |

## Supplementary Table 14. Function annotation of the genes were extracted within 10 kb of up and downstream of the chromosome rearrangement.

| **Species** | **Gene ID** | **UniProtKB** | **Function annotation** |
| --- | --- | --- | --- |
| *C. mandshurica* | CmaG0001181 | sp|Q8GX93|CLCE_ARATH | Chloride channel protein CLC-e OS=Arabidopsis thaliana OX=3702 GN=CLC-E PE=2 SV=2 |
|  | CmaG0001729 | sp\|Q9ZW09\|LRK31_ARATH | Probable inactive L-type lectin-domain containing receptor kinase III.1 OS=Arabidopsis thaliana OX=3702 GN=LECRK31 PE=3 SV=1 |
|  | CmaG0001730 | sp\|Q9FG33\|LRKS5_ARATH | Probable L-type lectin-domain containing receptor kinase S.5 OS=Arabidopsis thaliana OX=3702 GN=LECRKS5 PE=2 SV=1 |
|  | CmaG0001731 | sp\|Q9SZL6\|MTEF6_ARATH | Transcription termination factor MTERF6, chloroplastic/mitochondrial OS=Arabidopsis thaliana OX=3702 GN=MTERF6 PE=2 SV=1 |
|  | CmaG0001732 | sp\|Q9SZL5\|PP356_ARATH | Pentatricopeptide repeat-containing protein At4g38150 OS=Arabidopsis thaliana OX=3702 GN=At4g38150 PE=2 SV=1 |
|  | CmaG0008568 | sp\|Q588V7\|TEB_ARATH | Helicase and polymerase-containing protein TEBICHI OS=Arabidopsis thaliana OX=3702 GN=TEB PE=2 SV=1 |
|  | CmaG0008569 | sp\|Q9FFN2\|GLYT3_ARATH | Probable glycosyltransferase At5g03795 OS=Arabidopsis thaliana OX=3702 GN=At5g03795 PE=3 SV=2 |
|  | CmaG0021573 | sp\|B8AVF0\|BGL12_ORYSI | Beta-glucosidase 12 OS=Oryza sativa subsp. indica OX=39946 GN=BGLU12 PE=3 SV=1 |
|  | CmaG0021575 | sp\|P0C042\|Y4597_ARATH | Uncharacterized protein At4g15970 OS=Arabidopsis thaliana OX=3702 GN=At4g15970 PE=2 SV=1 |
|  | CmaG0022395 | sp\|P0DKI6\|Y1332_ARATH | Probable receptor-like protein kinase At1g33260 OS=Arabidopsis thaliana OX=3702 GN=At1g33260 PE=2 SV=1 |
|  | CmaG0022396 | sp\|Q56Y52\|POT1A_ARATH | Protection of telomeres protein 1a OS=Arabidopsis thaliana OX=3702 GN=POT1A PE=1 SV=1 |
|  | CmaG0001179 | - | - |
|  | CmaG0001180 | - | - |
|  | CmaG0008567 | - | - |
|  | CmaG0008570 | - | - |
|  | CmaG0008571 | - | - |
|  | CmaG0013895 | - | - |
|  | CmaG0021572 | - | - |
|  | CmaG0021574 | - | - |
|  |  |  |  |
| *Ca. fangianan* | Cfa001174 | sp|Q93YR3|F10AL_ARATH | FAM10 family protein At4g22670 OS=Arabidopsis thaliana OX=3702 GN=At4g22670 PE=1 SV=1 |
|  | Cfa002165 | sp\|Q9LF97\|Y3295_ARATH | CBS domain-containing protein CBSCBSPB3 OS=Arabidopsis thaliana OX=3702 GN=CBSCBSPB3 PE=1 SV=1 |
|  | Cfa003436 | sp\|Q9LJX5\|BRTL1_ARATH | Probable mitochondrial adenine nucleotide transporter BTL1 OS=Arabidopsis thaliana OX=3702 GN=At3g20240 PE=2 SV=1 |
|  | Cfa005756 | sp\|Q9LZQ9\|DEAH2_ARATH | Probable pre-mRNA-splicing factor ATP-dependent RNA helicase DEAH2 OS=Arabidopsis thaliana OX=3702 GN=At3g62310 PE=2 SV=1 |
|  | Cfa005757 | sp\|O22898\|LACS1_ARATH | Long chain acyl-CoA synthetase 1 OS=Arabidopsis thaliana OX=3702 GN=LACS1 PE=2 SV=1 |
|  | Cfa005758 | sp\|Q9SX45\|PPR75_ARATH | Pentatricopeptide repeat-containing protein At1g50270 OS=Arabidopsis thaliana OX=3702 GN=PCMP-E42 PE=2 SV=1 |
|  | Cfa008325 | sp\|Q9LSW9\|ATL16_ARATH | RING-H2 finger protein ATL16 OS=Arabidopsis thaliana OX=3702 GN=ATL16 PE=2 SV=1 |
|  | Cfa010314 | sp\|F4IZC4\|BASS4_ARATH | Probable sodium/metabolite cotransporter BASS4, chloroplastic OS=Arabidopsis thaliana OX=3702 GN=BASS4 PE=3 SV=1 |
|  | Cfa010316 | sp\|O23063\|STKLP_ARATH | STOREKEEPER protein OS=Solanum tuberosum OX=4113 GN=STK PE=2 SV=1 |
|  | Cfa011287 | sp\|F4IB81\|LYK3_ARATH | LysM domain receptor-like kinase 3 OS=Arabidopsis thaliana OX=3702 GN=LYK3 PE=2 SV=1 |
|  | Cfa012871 | sp\|O48809\|LRX2_ARATH | Leucine-rich repeat extensin-like protein 2 OS=Arabidopsis thaliana OX=3702 GN=LRX2 PE=2 SV=1 |
|  | Cfa012872 | sp\|Q5Z807\|C3H46_ORYSJ | Zinc finger CCCH domain-containing protein 46 OS=Oryza sativa subsp. japonica OX=39947 GN=LIC PE=1 SV=1 |
|  | Cfa014720 | sp\|Q9LW60\|P2C44_ARATH | Putative protein phosphatase 2C-like protein 44 OS=Arabidopsis thaliana OX=3702 GN=At3g23360 PE=5 SV=1 |
|  | Cfa014721 | sp\|Q9LW60\|P2C44_ARATH | Putative protein phosphatase 2C-like protein 44 OS=Arabidopsis thaliana OX=3702 GN=At3g23360 PE=5 SV=1 |
|  | Cfa017727 | sp\|Q9C9C0\|SPPA1_ARATH | Serine protease SPPA, chloroplastic OS=Arabidopsis thaliana OX=3702 GN=SPPA PE=2 SV=1 |
|  | Cfa017729 | sp\|Q9C9C0\|SPPA1_ARATH | Serine protease SPPA, chloroplastic OS=Arabidopsis thaliana OX=3702 GN=SPPA PE=2 SV=1 |
|  | Cfa019864 | sp\|Q5NBM8\|CSA_ORYSJ | Transcription factor CSA OS=Oryza sativa subsp. japonica OX=39947 GN=CSA PE=2 SV=2 |
|  | Cfa020185 | sp\|Q9LG26\|PARN_ARATH | Poly(A)-specific ribonuclease PARN OS=Arabidopsis thaliana OX=3702 GN=PARN PE=1 SV=2 |
|  | Cfa020186 | sp\|Q9T076\|ENL2_ARATH | Early nodulin-like protein 2 OS=Arabidopsis thaliana OX=3702 GN=At4g27520 PE=1 SV=1 |
|  | Cfa020628 | sp\|P0DO01\|DJA7A_ORYSJ | Chaperone protein dnaJ A7A, chloroplastic OS=Oryza sativa subsp. japonica OX=39947 GN=DJA7A PE=1 SV=1 |
|  | Cfa001173 | - | - |
|  | Cfa010315 | - | - |
|  | Cfa012870 | - | - |
|  | Cfa017728 | - | - |
|  | Cfa019616 | - | - |
|  |  |  |  |
| *Ostryopsis nobilis* | On001399 | sp|O48809|LRX2_ARATH | Leucine-rich repeat extensin-like protein 2 OS=Arabidopsis thaliana OX=3702 GN=LRX2 PE=2 SV=1 |
|  | On001400 | sp\|Q0JAA0\|P2C44_ORYSJ | Probable protein phosphatase 2C 44 OS=Oryza sativa subsp. japonica OX=39947 GN=Os04g0609600 PE=2 SV=1 |
|  | On001723 | sp\|P26585\|HMGL_SOYBN | HMG1/2-like protein OS=Glycine max OX=3847 PE=2 SV=1 |
|  | On005524 | sp\|O22446\|HDA19_ARATH | Histone deacetylase 19 OS=Arabidopsis thaliana OX=3702 GN=HDA19 PE=1 SV=2 |
|  | On005525 | sp\|Q8GX93\|CLCE_ARATH | Chloride channel protein CLC-e OS=Arabidopsis thaliana OX=3702 GN=CLC-E PE=2 SV=2 |
|  | On005782 | sp\|O81283\|TC159_ARATH | Translocase of chloroplast 159, chloroplastic OS=Arabidopsis thaliana OX=3702 GN=TOC159 PE=1 SV=1 |
|  | On005783 | sp\|Q9SW70\|SRP_VITRI | Stress-related protein OS=Vitis riparia OX=96939 GN=SRP PE=2 SV=1 |
|  | On006949 | sp\|Q9SSC7\|LOR5_ARATH | Protein LURP-one-related 5 OS=Arabidopsis thaliana OX=3702 GN=At1g80120 PE=2 SV=1 |
|  | On006950 | sp\|Q9ZVI9\|PECT1_ARATH | Ethanolamine-phosphate cytidylyltransferase OS=Arabidopsis thaliana OX=3702 GN=PECT1 PE=1 SV=1 |
|  | On007376 | sp\|Q8GXT3\|BH123_ARATH | Transcription factor bHLH123 OS=Arabidopsis thaliana OX=3702 GN=BHLH123 PE=1 SV=1 |
|  | On007377 | sp\|Q6NQE2\|FQRL1_ARATH | Probable NAD(P)H dehydrogenase (quinone) FQR1-like 1 OS=Arabidopsis thaliana OX=3702 GN=At4g27270 PE=1 SV=1 |
|  | On010288 | sp\|Q9FYJ6\|BH111_ARATH | Transcription factor bHLH111 OS=Arabidopsis thaliana OX=3702 GN=BHLH111 PE=2 SV=1 |
|  | On010517 | sp\|Q9FLS0\|FB253_ARATH | F-box protein At5g07610 OS=Arabidopsis thaliana OX=3702 GN=At5g07610 PE=2 SV=1 |
|  | On010518 | sp\|Q9C7Z9\|SCP18_ARATH | Serine carboxypeptidase-like 18 OS=Arabidopsis thaliana OX=3702 GN=SCPL18 PE=2 SV=2 |
|  | On010826 | sp\|Q9M0X5\|CRK25_ARATH | Cysteine-rich receptor-like protein kinase 25 OS=Arabidopsis thaliana OX=3702 GN=CRK25 PE=3 SV=1 |
|  | On010827 | sp\|Q9SN26\|PHL11_ARATH | Phytolongin Phyl1.1 OS=Arabidopsis thaliana OX=3702 GN=PHYL1.1 PE=2 SV=1 |
|  | On013995 | sp\|Q8W566\|Y3514_ARATH | Uncharacterized exonuclease domain-containing protein At3g15140 OS=Arabidopsis thaliana OX=3702 GN=At3g15140 PE=2 SV=1 |
|  | On013996 | sp\|Q9FMP3\|DPYS_ARATH | Dihydropyrimidinase OS=Arabidopsis thaliana OX=3702 GN=PYD2 PE=1 SV=1 |
|  | On014669 | sp\|Q9LW86\|SUT34_ARATH | Probable sulfate transporter 3.4 OS=Arabidopsis thaliana OX=3702 GN=SULTR3;4 PE=2 SV=1 |
|  | On015426 | sp\|Q9FMX6\|DJC76_ARATH | Chaperone protein dnaJ C76, chloroplastic OS=Arabidopsis thaliana OX=3702 GN=DJC76 PE=2 SV=1 |
|  | On019093 | sp\|Q8L4N1\|PHO34_ARATH | Universal stress protein PHOS34 OS=Arabidopsis thaliana OX=3702 GN=PHOS34 PE=1 SV=1 |
|  | On021731 | sp\|Q9M0X5\|CRK25_ARATH | Cysteine-rich receptor-like protein kinase 25 OS=Arabidopsis thaliana OX=3702 GN=CRK25 PE=3 SV=1 |
|  | On021732 | sp\|Q94KD3\|VP52A_ARATH | Vacuolar protein sorting-associated protein 52 A OS=Arabidopsis thaliana OX=3702 GN=VPS52 PE=1 SV=1 |
|  | On021733 | sp\|Q9M1S3\|ARP1_ARATH | Probable RNA-binding protein ARP1 OS=Arabidopsis thaliana OX=3702 GN=ARP1 PE=2 SV=1 |
|  | On023496 | sp\|Q8L796\|PI5K2_ARATH | Phosphatidylinositol 4-phosphate 5-kinase 2 OS=Arabidopsis thaliana OX=3702 GN=PIP5K2 PE=1 SV=2 |
|  | On023497 | sp\|Q9ZPW9\|NLTP8_ARATH | Non-specific lipid-transfer protein 8 OS=Arabidopsis thaliana OX=3702 GN=LTP8 PE=3 SV=1 |
|  | On023625 | sp\|Q8GUN2\|HINT1_ARATH | Adenylylsulfatase HINT1 OS=Arabidopsis thaliana OX=3702 GN=HINT1 PE=1 SV=1 |
|  | On001401 | - | - |
|  | On001722 | - | - |
|  | On001724 | - | - |
|  | On005784 | - | - |
|  | On011638 | - | - |
|  | On013690 | - | - |
|  | On013691 | - | - |
|  | On013997 | - | - |
|  | On015425 | - | - |
|  | On019092 | - | - |
|  | On023495 | - | - |
|  | On024053 | - | - |
|  |  |  |  |
| Ostrya rehderiana | OreG0000220 | sp|O81825|DRL28_ARATH | Probable disease resistance protein At4g27220 OS=Arabidopsis thaliana OX=3702 GN=At4g27220 PE=2 SV=1 |
|  | OreG0001881 | sp\|Q9LW60\|P2C44_ARATH | Putative protein phosphatase 2C-like protein 44 OS=Arabidopsis thaliana OX=3702 GN=At3g23360 PE=5 SV=1 |
|  | OreG0001882 | sp\|Q9LW60\|P2C44_ARATH | Putative protein phosphatase 2C-like protein 44 OS=Arabidopsis thaliana OX=3702 GN=At3g23360 PE=5 SV=1 |
|  | OreG0003441 | sp\|Q9FJH0\|RAA1F_ARATH | Ras-related protein RABA1f OS=Arabidopsis thaliana OX=3702 GN=RABA1F PE=2 SV=1 |
|  | OreG0003442 | sp\|Q8RXK2\|SDN3_ARATH | RNA exonuclease 4 OS=Homo sapiens OX=9606 GN=REXO4 PE=1 SV=2 |
|  | OreG0006248 | sp\|Q9SCY2\|FKB13_ARATH | Peptidyl-prolyl cis-trans isomerase FKBP13, chloroplastic OS=Arabidopsis thaliana OX=3702 GN=FKBP13 PE=1 SV=2 |
|  | OreG0006249 | sp\|Q9C6T2\|PPR68_ARATH | Pentatricopeptide repeat-containing protein At1g31920 OS=Arabidopsis thaliana OX=3702 GN=PCMP-H11 PE=2 SV=1 |
|  | OreG0007627 | sp\|Q8RXD3\|AIP2_ARATH | E3 ubiquitin-protein ligase AIP2 OS=Arabidopsis thaliana OX=3702 GN=AIP2 PE=1 SV=1 |
|  | OreG0010187 | sp\|Q8VZL6\|SWC4_ARATH | SWR1-complex protein 4 OS=Arabidopsis thaliana OX=3702 GN=SWC4 PE=1 SV=1 |
|  | OreG0013876 | sp\|O49931\|TIC55_PEA | Protein TIC 55, chloroplastic OS=Pisum sativum OX=3888 GN=TIC55 PE=1 SV=1 |
|  | OreG0016308 | sp\|Q8VY07\|EPN1_ARATH | Clathrin interactor EPSIN 1 OS=Arabidopsis thaliana OX=3702 GN=EPSIN1 PE=1 SV=1 |
|  | OreG0017221 | sp\|Q9SPM5\|APY2_ARATH | Apyrase 2 OS=Arabidopsis thaliana OX=3702 GN=APY2 PE=1 SV=1 |
|  | OreG0017222 | sp\|Q9LFQ4\|PP383_ARATH | Pentatricopeptide repeat-containing protein At5g15010, mitochondrial OS=Arabidopsis thaliana OX=3702 GN=At5g15010 PE=2 SV=2 |
|  | OreG0017720 | sp\|Q84JR9\|TTL4_ARATH | TPR repeat-containing thioredoxin TTL4 OS=Arabidopsis thaliana OX=3702 GN=TTL4 PE=2 SV=1 |
|  | OreG0019687 | sp\|P08926\|RUBA_PEA | RuBisCO large subunit-binding protein subunit alpha, chloroplastic OS=Pisum sativum OX=3888 PE=1 SV=2 |
|  | OreG0019688 | sp\|Q9FTA2\|TCP21_ARATH | Transcription factor TCP21 OS=Arabidopsis thaliana OX=3702 GN=TCP21 PE=1 SV=1 |
|  | OreG0000538 | - | - |
|  | OreG0000539 | - | - |
|  | OreG0000540 | - | - |
|  | OreG0003440 | - | - |
|  | OreG0004367 | - | - |
|  | OreG0004368 | - | - |
|  | OreG0006246 | - | - |
|  | OreG0006247 | - | - |
|  | OreG0007626 | - | - |
|  | OreG0010250 | - | - |
|  | OreG0016307 | - | - |
|  | OreG0017720 | - | - |
|  | OreG0017721 | - | - |
|  | OreG0018511 | - | - |
|  | OreG0019821 | - | - |
|  | OreG0019822 | - | - |
|  | OreG0019823 | - | - |

## Supplementary Table 15. Gene ontology (GO) enrichment analysis for genes of the rapid expand families in *C. mandshurica* genome.

| **GO ID** | **Type** | **Function** | **Number of ecriched genes** | **Number of genes of background** | **Adjusted P-value** |
| --- | --- | --- | --- | --- | --- |
| GO:0000271 | BP | polysaccharide biosynthetic process | 6 | 67 | 0.001765 |
| GO:0001101 | BP | response to acid chemical | 6 | 31 | 4.08E-05 |
| GO:0001871 | MF | pattern binding | 5 | 47 | 0.002132 |
| GO:0003674 | MF | MF | 279 | 13899 | 6.65E-11 |
| GO:0004222 | MF | metalloendopeptidase activity | 7 | 51 | 6.67E-05 |
| GO:0004497 | MF | monooxygenase activity | 9 | 72 | 1.20E-05 |
| GO:0004499 | MF | N,N-dimethylaniline monooxygenase activity | 9 | 38 | 1.06E-07 |
| GO:0004519 | MF | endonuclease activity | 7 | 60 | 0.000169 |
| GO:0004521 | MF | endoribonuclease activity | 7 | 41 | 1.90E-05 |
| GO:0004523 | MF | RNA-DNA hybrid ribonuclease activity | 7 | 11 | 1.29E-08 |
| GO:0004540 | MF | ribonuclease activity | 7 | 58 | 0.000139 |
| GO:0004568 | MF | chitinase activity | 5 | 26 | 0.000188 |
| GO:0004650 | MF | polygalacturonase activity | 8 | 63 | 3.34E-05 |
| GO:0004857 | MF | enzyme inhibitor activity | 12 | 127 | 6.24E-06 |
| GO:0004866 | MF | endopeptidase inhibitor activity | 12 | 27 | 1.74E-12 |
| GO:0004867 | MF | serine-type endopeptidase inhibitor activity | 6 | 10 | 1.92E-07 |
| GO:0005488 | MF | binding | 232 | 9124 | 7.77E-18 |
| GO:0005507 | MF | copper ion binding | 13 | 106 | 1.70E-07 |
| GO:0005576 | CC | extracellular region | 11 | 132 | 4.47E-05 |
| GO:0006022 | BP | aminoglycan metabolic process | 5 | 27 | 0.00022 |
| GO:0006026 | BP | aminoglycan catabolic process | 5 | 26 | 0.000188 |
| GO:0006030 | BP | chitin metabolic process | 5 | 26 | 0.000188 |
| GO:0006032 | BP | chitin catabolic process | 5 | 26 | 0.000188 |
| GO:0006040 | BP | amino sugar metabolic process | 5 | 29 | 0.000295 |
| GO:0006807 | BP | nitrogen compound metabolic process | 5 | 1649 | 2.75E-08 |
| GO:0008061 | MF | chitin binding | 5 | 31 | 0.000388 |
| GO:0008150 | BP | BP | 122 | 9507 | 1.54E-08 |
| GO:0008237 | MF | metallopeptidase activity | 7 | 70 | 0.000402 |
| GO:0008270 | MF | zinc ion binding | 28 | 532 | 6.66E-07 |
| GO:0009250 | BP | glucan biosynthetic process | 6 | 58 | 0.000886 |
| GO:0009611 | BP | response to wounding | 6 | 9 | 1.22E-07 |
| GO:0009698 | BP | phenylpropanoid metabolic process | 11 | 46 | 3.63E-09 |
| GO:0009751 | BP | response to salicylic acid | 6 | 16 | 1.64E-06 |
| GO:0009808 | BP | lignin metabolic process | 11 | 46 | 3.63E-09 |
| GO:0009966 | BP | regulation of signal transduction | 6 | 39 | 0.000127 |
| GO:0009987 | BP | cellular process | 46 | 4471 | 3.63E-06 |
| GO:0010181 | MF | FMN binding | 6 | 36 | 8.56E-05 |
| GO:0010646 | BP | regulation of cell communication | 6 | 39 | 0.000127 |
| GO:0014070 | BP | response to organic cyclic compound | 6 | 16 | 1.64E-06 |
| GO:0016679 | MF | oxidoreductase activity, acting on diphenols and related substances as donors | 11 | 60 | 4.05E-08 |
| GO:0016682 | MF | oxidoreductase activity, acting on diphenols and related substances as donors, oxygen as acceptor | 11 | 56 | 2.16E-08 |
| GO:0016709 | MF | oxidoreductase activity, acting on paired donors, with incorporation or reduction of molecular oxygen, NAD(P)H as one donor, and incorporation of one atom of oxygen | 9 | 44 | 3.14E-07 |
| GO:0016759 | MF | cellulose synthase activity | 6 | 39 | 0.000127 |
| GO:0016760 | MF | cellulose synthase (UDP-forming) activity | 6 | 39 | 0.000127 |
| GO:0016891 | MF | endoribonuclease activity, producing 5'-phosphomonoesters | 7 | 24 | 8.75E-07 |
| GO:0016893 | MF | endonuclease activity, active with either ribo- or deoxyribonucleic acids and producing 5'-phosphomonoesters | 7 | 26 | 1.38E-06 |
| GO:0016998 | BP | cell wall macromolecule catabolic process | 5 | 26 | 0.000188 |
| GO:0019439 | BP | aromatic compound catabolic process | 11 | 89 | 1.43E-06 |
| GO:0019748 | BP | secondary metabolic process | 11 | 48 | 5.33E-09 |
| GO:0023051 | BP | regulation of signaling | 6 | 39 | 0.000127 |
| GO:0030145 | MF | manganese ion binding | 28 | 107 | 2.48E-22 |
| GO:0030234 | MF | enzyme regulator activity | 12 | 209 | 0.000549 |
| GO:0030243 | BP | cellulose metabolic process | 6 | 40 | 0.000144 |
| GO:0030244 | BP | cellulose biosynthetic process | 6 | 39 | 0.000127 |
| GO:0030247 | MF | polysaccharide binding | 5 | 47 | 0.002132 |
| GO:0030414 | MF | peptidase inhibitor activity | 12 | 27 | 1.74E-12 |
| GO:0031012 | CC | extracellular matrix | 7 | 13 | 3.04E-08 |
| GO:0031347 | BP | regulation of defense response | 6 | 24 | 1.16E-05 |
| GO:0033692 | BP | cellular polysaccharide biosynthetic process | 6 | 67 | 0.001765 |
| GO:0035251 | MF | UDP-glucosyltransferase activity | 6 | 67 | 0.001765 |
| GO:0043167 | MF | ion binding | 70 | 1851 | 1.54E-09 |
| GO:0043169 | MF | cation binding | 70 | 1745 | 1.28E-10 |
| GO:0044238 | BP | primary metabolic process | 44 | 4113 | 3.65E-05 |
| GO:0044248 | BP | cellular catabolic process | 16 | 281 | 7.48E-05 |
| GO:0044421 | CC | extracellular region part | 7 | 22 | 5.35E-07 |
| GO:0044712 | BP | single-organism catabolic process | 11 | 157 | 0.00019 |
| GO:0045735 | MF | nutrient reservoir activity | 28 | 118 | 2.39E-21 |
| GO:0046271 | BP | phenylpropanoid catabolic process | 11 | 46 | 3.63E-09 |
| GO:0046274 | BP | lignin catabolic process | 11 | 46 | 3.63E-09 |
| GO:0046348 | BP | amino sugar catabolic process | 5 | 26 | 0.000188 |
| GO:0046527 | MF | glucosyltransferase activity | 6 | 68 | 0.001893 |
| GO:0046872 | MF | metal ion binding | 70 | 1704 | 6.92E-11 |
| GO:0046914 | MF | transition metal ion binding | 70 | 1116 | 5.40E-20 |
| GO:0048046 | CC | apoplast | 11 | 72 | 2.12E-07 |
| GO:0048583 | BP | regulation of response to stimulus | 6 | 57 | 0.000815 |
| GO:0050661 | MF | NADP binding | 9 | 69 | 8.82E-06 |
| GO:0051273 | BP | beta-glucan metabolic process | 6 | 52 | 0.000522 |
| GO:0051274 | BP | beta-glucan biosynthetic process | 6 | 51 | 0.000475 |
| GO:0052716 | MF | hydroquinone:oxygen oxidoreductase activity | 11 | 46 | 3.63E-09 |
| GO:0061134 | MF | peptidase regulator activity | 12 | 29 | 3.41E-12 |
| GO:0061135 | MF | endopeptidase regulator activity | 12 | 27 | 1.74E-12 |
| GO:0070887 | BP | cellular response to chemical stimulus | 6 | 16 | 1.64E-06 |
| GO:0071229 | BP | cellular response to acid chemical | 6 | 16 | 1.64E-06 |
| GO:0071310 | BP | cellular response to organic substance | 6 | 16 | 1.64E-06 |
| GO:0071407 | BP | cellular response to organic cyclic compound | 6 | 16 | 1.64E-06 |
| GO:0071446 | BP | cellular response to salicylic acid stimulus | 6 | 16 | 1.64E-06 |
| GO:0080134 | BP | regulation of response to stress | 6 | 28 | 2.47E-05 |
| GO:1901071 | BP | glucosamine-containing compound metabolic process | 5 | 26 | 0.000188 |
| GO:1901072 | BP | glucosamine-containing compound catabolic process | 5 | 26 | 0.000188 |
| GO:1901136 | BP | carbohydrate derivative catabolic process | 5 | 36 | 0.000719 |
| GO:1901361 | BP | organic cyclic compound catabolic process | 11 | 90 | 1.58E-06 |
| GO:1901575 | BP | organic substance catabolic process | 16 | 334 | 0.000481 |
| GO:1901700 | BP | response to oxygen-containing compound | 6 | 33 | 5.56E-05 |
| GO:1901701 | BP | cellular response to oxygen-containing compound | 6 | 16 | 1.64E-06 |
| GO:2000031 | BP | regulation of salicylic acid mediated signaling pathway | 6 | 16 | 1.64E-06 |

| **Species and Type** | **Abbreviation** | **Ath** | **Bpe** | **Cav** | **Ceq** | **Cfa** | **Cma** | **Fve** | **Jre** | **Ono** | **Ore** | **Ppe** | **Qro** |
| --- | --- | --- | --- | --- | --- | --- | --- | --- | --- | --- | --- | --- | --- |
| **non-TIR NBS subclass** |  | 77 | 61 | 249 | 106 | 86 | 78 | 161 | 86 | 47 | 70 | 267 | 672 |
| **nRNs** |  | 68 | 58 | 233 | 95 | 77 | 67 | 130 | 77 | 40 | 59 | 243 | 607 |
| BED_CC_NBS_LRR | BCNL | 0 | 0 | 0 | 0 | 0 | 0 | 0 | 0 | 0 | 0 | 0 | 5 |
| CC-NBS-LRR | CNL | 39 | 8 | 75 | 16 | 21 | 26 | 53 | 17 | 16 | 12 | 102 | 223 |
| NBS-LRR | NL | 12 | 21 | 62 | 32 | 24 | 6 | 32 | 29 | 9 | 29 | 89 | 265 |
| NBS only | N | 17 | 27 | 83 | 43 | 31 | 35 | 42 | 31 | 15 | 18 | 52 | 114 |
| **RN** |  | 9 | 3 | 26 | 11 | 9 | 11 | 31 | 9 | 7 | 11 | 24 | 65 |
| RPW8-CC-NBS-LRR | RCNL | 4 | 1 | 14 | 6 | 4 | 7 | 14 | 5 | 3 | 6 | 10 | 30 |
| RPW8-NBS-LRR | RNL | 4 | 2 | 5 | 2 | 4 | 2 | 11 | 3 | 4 | 5 | 11 | 20 |
| RPW8-CC-NBS | RCN | 0 | 0 | 5 | 2 | 1 | 2 | 0 | 1 | 0 | 0 | 2 | 6 |
| RPW8-NBS | RN | 1 | 0 | 2 | 1 | 0 | 0 | 6 | 0 | 0 | 0 | 1 | 9 |
| **TIR NBS subclass** |  | 101 | 57 | 55 | 38 | 2 | 2 | 35 | 2 | 2 | 3 | 148 | 211 |
| TIR-CC-NBS-LRR | TCNL | 9 | 5 | 3 | 2 | 1 | 1 | 4 | 1 | 1 | 1 | 16 | 22 |
| TIR-NBS-LRR | TNL | 75 | 40 | 29 | 17 | 0 | 0 | 17 | 0 | 0 | 1 | 115 | 173 |
| TIR-CC-NBS | TCN | 3 | 0 | 0 | 2 | 0 | 0 | 4 | 0 | 0 | 0 | 0 | 0 |
| TIR-NBS | TN | 14 | 12 | 23 | 17 | 1 | 1 | 10 | 1 | 1 | 1 | 17 | 16 |
| Ratio of nTNs to TNs |  | 0.76 | 1.07 | 4.53 | 2.79 | 43.00 | 39 | 4.60 | 43.00 | 23.50 | 23.33 | 1.80 | 3.18 |
| **Total** |  | 178 | 116 | 301 | 140 | 87 | 80 | 193 | 88 | 49 | 73 | 415 | 883 |

## Supplementary Table 16. Cross-species comparison of number of R genes. The corresponding relationship between abbreviations and full names are listing as follow: Ath: *A. thaliana*; Bpe: *B. pendula*; Cav: *C. avellana*; Ceq: *Cas. equisetifolia*; Cfa: *Ca. fangianan*; Cma: *C. mandshurica*; Fve: *F. vesca*; Jre: *J. regia*; Ono: *Ostryopsis nobilis*; Ore: *Ostrya rehderiana*; Ppe: *Prunus persica*; Qro: *Quercus robur*; Vvi: *Vitis vinifera*.

## Supplementary Table 18. Rapid evolving genes (REGs) and positively selected genes (PSGs) identified belong to *C. mandshurica*. Abbreviations are as follow: LACS: Long-chain Acyl-CoA synthase; GPAT: glycerol-3-phosphate acyltransferase; FATA: fatty acyl-ACP thioesterase A; KAS II: β-ketoacyl-[acyl carrier protein] synthase II; PDCT: phosphatidylcholine:diacylglycerol cholinephosphotransferase; MYB: v-myb avian myeloblastosis viral oncogene homolog; APETALA: 2/ethylene response factor; CAMTA: Calmodulin-binding transcription activator; RCNL: Resistance to powdery mildew8 coiled-coil nucleotide binding site leucine-rich repeat; NBS: nucleotide binding site.

| Annotation | | Gene ID | Type | *P*-value |
| --- | --- | --- | --- | --- |
| Oil biosynthesis | LACS | CmaG0017938.1 | PSG | 1.54E-02 |
|  | SAD | CmaG0006952.1 | PSG | 4.80E-03 |
|  | GPAT | CmaG0002649.1 | PSG | 4.28E-02 |
|  |  | CmaG0000316.1 | REG | 1.08E-02 |
|  |  | CmaG0002799.1 | REG | 4.89E-02 |
|  |  | CmaG0006304.1 | REG | 2.42E-02 |
|  | FATA | CmaG0012678.1 | REG | 1.90E-03 |
|  |  | CmaG0017749.1 | REG | 2.30E-02 |
|  | KASII | CmaG0024421.1 | REG | 3.89E-03 |
|  | PDCT | CmaG0025179.1 | REG | 2.80E-02 |
| Stress responses related  transcription factor families | MYB | CmaG0005328.1 | PSG | 5.41E-03 |
|  |  | CmaG0005821.1 | PSG | 2.84E-02 |
|  |  | CmaG0011452.1 | PSG | 2.73E-02 |
|  |  | CmaG0011769.1 | PSG | 0.00E+00 |
|  |  | CmaG0014234.1 | PSG | 9.36E-03 |
|  |  | CmaG0017264.1 | PSG | 4.56E-02 |
|  |  | CmaG0020449.1 | PSG | 1.06E-03 |
|  |  | CmaG0020513.1 | PSG | 1.33E-05 |
|  |  | CmaG0023132.1 | PSG | 1.68E-07 |
|  |  | CmaG0026425.1 | PSG | 1.38E-07 |
|  |  | CmaG0000102.1 | REG | 3.02E-02 |
|  |  | CmaG0005562.1 | REG | 3.97E-02 |
|  |  | CmaG0007001.1 | REG | 4.83E-02 |
|  |  | CmaG0009288.1 | REG | 2.22E-04 |
|  |  | CmaG0009834.1 | REG | 3.69E-02 |
|  |  | CmaG0011191.1 | REG | 1.76E-03 |
|  |  | CmaG0011572.1 | REG | 1.60E-05 |
|  |  | CmaG0012074.1 | REG | 1.27E-02 |
|  |  | CmaG0012609.1 | REG | 1.49E-02 |
|  |  | CmaG0014955.1 | REG | 6.79E-03 |
|  |  | CmaG0015180.1 | REG | 1.98E-02 |
|  |  | CmaG0015402.1 | REG | 4.40E-05 |
|  |  | CmaG0017048.1 | REG | 1.99E-02 |
|  |  | CmaG0017675.1 | REG | 1.20E-05 |
|  |  | CmaG0023299.1 | REG | 2.06E-02 |
|  | AP2/ERF-ERF | CmaG0004045.1 | PSG | 0.00E+00 |
|  |  | CmaG0006984.1 | PSG | 0.00E+00 |
|  |  | CmaG0009375.1 | PSG | 4.07E-02 |
|  |  | CmaG0006147.1 | REG | 1.13E-03 |
|  |  | CmaG0006229.1 | REG | 1.51E-02 |
|  |  | CmaG0013661.1 | REG | 3.37E-02 |
|  |  | CmaG0019674.1 | REG | 1.12E-03 |
|  |  | CmaG0022905.1 | REG | 4.97E-02 |
|  |  | CmaG0023524.1 | REG | 9.00E-03 |
|  | CAMTA | CmaG0003421.1 | PSG | 0.00E+00 |
| R genes | RCNL | CmaG0017824.1 | REG | 1.08E-02 |
|  | NBS only | CmaG0019218.1 | REG | 1.59E-02 |

**Supplementary Table 19. The software version and parameters applications in this study.**

| **Software and Algorithms** | **Parameters** |
| --- | --- |
| fastp (v.0.20.0) | default |
| Jellyfish (v.2.2.10) | jellyfish count /dev/fd/0 -C -o Cma_21mer -m 21 -t 48 -s 50G; jellyfish histo -h 5000000 -o Cma_21mer.histo Cma_21mer |
| GenomeScope (v1.0.0) | genomescope.R Cma_out.histo 21 150 Cma_21mer |
| NextDenovo (v.2.1) | read_cutoff = 8k seed_cutoff = 9917 |
| NextPolish (v.1.1) | lgs_options = -min_read_len 10k -max_read_len 210k -max_depth 30 lgs_minimap2_options = -x map-ont -t 90 |
| Purge_Haplotigs | purge -a=60 |
| BUSCO (v.3) | embryophyta_odb10 -m genome -c 20 -sp arabidopsis |
| HiCUP | default |
| ALLHiC (v0.8.12) | –e=GATC–k=11–RE=GATC |
| RepeatMasker | -nolow -norna -no_is -gff -species Mesangiospermae |
| RepeatProteinMasker | -noLowSimple -pvalue 1e-04 |
| RepeatModeler | default |
| LTR_Finder (v1.06) | default |
| bedtools (v.2.29.2) | bedtools merge -i All.repeat.bed > All.repeat.merge.bed; bedtools maskfasta -fi curated.fasta -bed All.repeat.merge.bed -fo curated.fasta.mask |
| Augustus (v.3.2.3) | --species=arabidopsis |
| GenScan | Arabidopsis.smat |
| GlimmerHMM (v.3.0.4) | -d arabidopsis |
| GoMoMa (v.1.6) | AnnotationFinalizer.r=NO tblastn=false |
| Trinity (v.2.1.1) | --seqType fq --max_memory 120G --CPU 30 --full_cleanup --trimmomatic --normalize_reads |
| PASA (v2.4.1) | Launch_PASA_pipeline.pl -c pasa.config -C -R -g genome.fasta -t Trinity.fasta --ALIGNERS blat,gmap --CPU 30 |
| EVidenceModeler (v.1.1.1) | evidence_modeler.pl -G genome.fasta -g ab_initio.gff -w evm.weights.txt -e rna_seq.gff -p homolog.gff --exec_dir evm_out > evm.out |
| BLASTP (v.2.7.1+) | E-value < 1 × 10^−5^ |
| InterProScan (v.5.28) | interproscan.sh -appl TIGRFAM,ProDom,Hamap,SMART,ProSiteProfiles,ProSitePatterns,SUPERFAMILY,PRINTS,Gene3D,PIRSF,Pfam,Coils -f tsv -iprlookup -goterms -pa -t p -td ./temp |
| Hmmer (v3.1b2) | default |
| OrthoMCL (v.2.0.9-4) | default |
| iqtree (v.2.0.3) | -s concatenation.fa -st DNA -pre concatenation.fa -nt 5 -bb 1000 -m MFP -quiet -redo |
| PAML (v.4.8) | baseml baseml.ctl; mcmctree mcmctree.ctl > mcmctree.log |
| CAFÉ (v.2.2) | cafe2.2 cafetutorial_run1.sh |
| Sonicparanoid (v.1.0.13) | default |
| MCScanX | python -m jcvi.formats.gff bed --type=mRNA --key=ID genome.gff -o Sp.bed; python -m jcvi.compara.catalog ortholog Sp1 Sp2; python -m jcvi.compara.synteny screen --minspan=30 --simple lifted.anchors anchors.new; python -m jcvi.graphics.karyotype seqids layout |
| WGDI | default |
