# Supplementary material for: The Corylus mandshurica genome provides insights into the evolution of Betulaceae genomes and hazelnut breeding
Source: Hortic Res. 2021 Mar 1;8:54. doi: 10.1038/s41438-021-00495-1 (PMC7917096; doi:10.1038/s41438-021-00495-1)
Supplement: Supplementary file 2 — Supplement Table 17 [file 41438_2021_495_MOESM2_ESM.pdf]

Supplementary Table 17. Total number of oil-related gene families in the pilose filbert genome

Gene ID	
4-Coumarate-CoA Ligase	
CmaG0006563.1	4-coumarate--CoA ligase 1 OS=Nicotiana tabacum OX=4097 GN=4CL1 PE=2 SV=1
CmaG0025479.1	4-coumarate--CoA ligase 1 OS=Nicotiana tabacum OX=4097 GN=4CL1 PE=2 SV=1
CmaG0002491.1	4-coumarate--CoA ligase 2 OS=Glycine max OX=3847 PE=2 SV=2
CmaG0019849.1	4-coumarate--CoA ligase-like 1 OS=Arabidopsis thaliana OX=3702 GN=4CLL1 PE=1 SV=1
CmaG0027510.1	4-coumarate--CoA ligase-like 2 OS=Arabidopsis thaliana OX=3702 GN=4CLL2 PE=2 SV=2
CmaG0024814.1	4-coumarate--CoA ligase-like 5 OS=Arabidopsis thaliana OX=3702 GN=4CLL5 PE=1 SV=2
CmaG0025136.1	4-coumarate--CoA ligase-like 5 OS=Arabidopsis thaliana OX=3702 GN=4CLL5 PE=1 SV=2
CmaG0021035.1	4-coumarate--CoA ligase-like 6 OS=Arabidopsis thaliana OX=3702 GN=4CLL6 PE=2 SV=2
CmaG0009323.1	4-coumarate--CoA ligase-like 7 OS=Arabidopsis thaliana OX=3702 GN=4CLL7 PE=1 SV=1
CmaG0009325.1	4-coumarate--CoA ligase-like 7 OS=Arabidopsis thaliana OX=3702 GN=4CLL7 PE=1 SV=1
CmaG0009326.1	4-coumarate--CoA ligase-like 7 OS=Arabidopsis thaliana OX=3702 GN=4CLL7 PE=1 SV=1
CmaG0011857.1	4-coumarate--CoA ligase-like 7 OS=Arabidopsis thaliana OX=3702 GN=4CLL7 PE=1 SV=1
CmaG0011858.1	4-coumarate--CoA ligase-like 7 OS=Arabidopsis thaliana OX=3702 GN=4CLL7 PE=1 SV=1
CmaG0011859.1	4-coumarate--CoA ligase-like 7 OS=Arabidopsis thaliana OX=3702 GN=4CLL7 PE=1 SV=1
CmaG0015557.1	4-coumarate--CoA ligase-like 9 OS=Arabidopsis thaliana OX=3702 GN=4CLL9 PE=1 SV=2
CmaG0015558.1	4-coumarate--CoA ligase-like 9 OS=Arabidopsis thaliana OX=3702 GN=4CLL9 PE=1 SV=2
CmaG0020178.1	4-coumarate--CoA ligase-like 9 OS=Arabidopsis thaliana OX=3702 GN=4CLL9 PE=1 SV=2
ABC transporter	
CmaG0008043.1	ABC transporter A family member 1 OS=Arabidopsis thaliana OX=3702 GN=ABCA1 PE=2 SV=2
CmaG0020278.1	ABC transporter A family member 2 OS=Arabidopsis thaliana OX=3702 GN=ABCA2 PE=2 SV=1
CmaG0020280.1	ABC transporter A family member 2 OS=Arabidopsis thaliana OX=3702 GN=ABCA2 PE=2 SV=1
CmaG0020275.1	ABC transporter A family member 7 OS=Arabidopsis thaliana OX=3702 GN=ABCA7 PE=3 SV=2
CmaG0009322.1	ABC transporter B family member 1 OS=Arabidopsis thaliana OX=3702 GN=ABCB1 PE=1 SV=1
CmaG0002880.1	ABC transporter B family member 11 OS=Arabidopsis thaliana OX=3702 GN=ABCB11 PE=2 SV=1
CmaG0013819.1	ABC transporter B family member 11 OS=Arabidopsis thaliana OX=3702 GN=ABCB11 PE=2 SV=1
CmaG0013820.1	ABC transporter B family member 11 OS=Arabidopsis thaliana OX=3702 GN=ABCB11 PE=2 SV=1
CmaG0013821.1	ABC transporter B family member 11 OS=Arabidopsis thaliana OX=3702 GN=ABCB11 PE=2 SV=1
CmaG0013823.1	ABC transporter B family member 11 OS=Arabidopsis thaliana OX=3702 GN=ABCB11 PE=2 SV=1
CmaG0013825.1	ABC transporter B family member 11 OS=Arabidopsis thaliana OX=3702 GN=ABCB11 PE=2 SV=1
CmaG0024908.1	ABC transporter B family member 13 OS=Arabidopsis thaliana OX=3702 GN=ABCB13 PE=3 SV=1
CmaG0015308.1	ABC transporter B family member 15 OS=Arabidopsis thaliana OX=3702 GN=ABCB15 PE=3 SV=1
CmaG0019712.1	ABC transporter B family member 15 OS=Arabidopsis thaliana OX=3702 GN=ABCB15 PE=3 SV=1
CmaG0007488.1	ABC transporter B family member 19 OS=Arabidopsis thaliana OX=3702 GN=ABCB19 PE=1 SV=1
CmaG0015233.1	ABC transporter B family member 19 OS=Arabidopsis thaliana OX=3702 GN=ABCB19 PE=1 SV=1
CmaG0021098.1	ABC transporter B family member 2 OS=Arabidopsis thaliana OX=3702 GN=ABCB2 PE=1 SV=3
CmaG0027808.1	ABC transporter B family member 20 OS=Arabidopsis thaliana OX=3702 GN=ABCB20 PE=1 SV=1
CmaG0013827.1	ABC transporter B family member 21 OS=Arabidopsis thaliana OX=3702 GN=ABCB21 PE=1 SV=2
CmaG0013828.1	ABC transporter B family member 21 OS=Arabidopsis thaliana OX=3702 GN=ABCB21 PE=1 SV=2
CmaG0015080.1	ABC transporter B family member 25 OS=Oryza sativa subsp. japonica OX=39947 GN=ABCB25 PE=2 SV=1
CmaG0026695.1	ABC transporter B family member 25, mitochondrial OS=Arabidopsis thaliana OX=3702 GN=ABCB25 PE=1 SV=1
CmaG0015462.1	ABC transporter B family member 26, chloroplastic OS=Arabidopsis thaliana OX=3702 GN=ABCB26 PE=1 SV=1

CmaG0019667.1	ABC transporter B family member 26, chloroplastic OS=Arabidopsis thaliana OX=3702 GN=ABCB26 PE=1 SV=1
CmaG0015076.1	ABC transporter B family member 27 OS=Arabidopsis thaliana OX=3702 GN=ABCB27 PE=1 SV=1
CmaG0014297.1	ABC transporter B family member 28 OS=Arabidopsis thaliana OX=3702 GN=ABCB28 PE=2 SV=1
CmaG0007823.1	ABC transporter B family member 29, chloroplastic OS=Arabidopsis thaliana OX=3702 GN=ABCB29 PE=1 SV=1
CmaG0013822.1	ABC transporter B family member 4 OS=Arabidopsis thaliana OX=3702 GN=ABCB4 PE=1 SV=1
CmaG0013824.1	ABC transporter B family member 4 OS=Arabidopsis thaliana OX=3702 GN=ABCB4 PE=1 SV=1
CmaG0013826.1	ABC transporter B family member 4 OS=Arabidopsis thaliana OX=3702 GN=ABCB4 PE=1 SV=1
CmaG0013829.1	ABC transporter B family member 4 OS=Arabidopsis thaliana OX=3702 GN=ABCB4 PE=1 SV=1
CmaG0013830.1	ABC transporter B family member 4 OS=Arabidopsis thaliana OX=3702 GN=ABCB4 PE=1 SV=1
CmaG0024527.1	ABC transporter B family member 9 OS=Arabidopsis thaliana OX=3702 GN=ABCB9 PE=3 SV=2
CmaG0002123.1	ABC transporter C family member 10 OS=Arabidopsis thaliana OX=3702 GN=ABCC10 PE=2 SV=2
CmaG0002124.1	ABC transporter C family member 10 OS=Arabidopsis thaliana OX=3702 GN=ABCC10 PE=2 SV=2
CmaG0002125.1	ABC transporter C family member 10 OS=Arabidopsis thaliana OX=3702 GN=ABCC10 PE=2 SV=2
CmaG0002126.1	ABC transporter C family member 10 OS=Arabidopsis thaliana OX=3702 GN=ABCC10 PE=2 SV=2
CmaG0021831.1	ABC transporter C family member 10 OS=Arabidopsis thaliana OX=3702 GN=ABCC10 PE=2 SV=2
CmaG0021832.1	ABC transporter C family member 10 OS=Arabidopsis thaliana OX=3702 GN=ABCC10 PE=2 SV=2
CmaG0021833.1	ABC transporter C family member 10 OS=Arabidopsis thaliana OX=3702 GN=ABCC10 PE=2 SV=2
CmaG0027388.1	ABC transporter C family member 13 OS=Arabidopsis thaliana OX=3702 GN=ABCC13 PE=2 SV=3
CmaG0024241.1	ABC transporter C family member 2 OS=Arabidopsis thaliana OX=3702 GN=ABCC2 PE=1 SV=2
CmaG0024242.1	ABC transporter C family member 2 OS=Arabidopsis thaliana OX=3702 GN=ABCC2 PE=1 SV=2
CmaG0019761.1	ABC transporter C family member 3 OS=Arabidopsis thaliana OX=3702 GN=ABCC3 PE=1 SV=1
CmaG0022989.1	ABC transporter C family member 3 OS=Arabidopsis thaliana OX=3702 GN=ABCC3 PE=1 SV=1
CmaG0022990.1	ABC transporter C family member 3 OS=Arabidopsis thaliana OX=3702 GN=ABCC3 PE=1 SV=1
CmaG0022992.1	ABC transporter C family member 3 OS=Arabidopsis thaliana OX=3702 GN=ABCC3 PE=1 SV=1
CmaG0013593.1	ABC transporter C family member 4 OS=Arabidopsis thaliana OX=3702 GN=ABCC4 PE=1 SV=2
CmaG0016111.1	ABC transporter C family member 5 OS=Arabidopsis thaliana OX=3702 GN=ABCC5 PE=2 SV=2
CmaG0022991.1	ABC transporter C family member 6 OS=Arabidopsis thaliana OX=3702 GN=ABCC6 PE=2 SV=3
CmaG0007328.1	ABC transporter C family member 8 OS=Arabidopsis thaliana OX=3702 GN=ABCC8 PE=2 SV=3
CmaG0007330.1	ABC transporter C family member 8 OS=Arabidopsis thaliana OX=3702 GN=ABCC8 PE=2 SV=3
CmaG0000715.1	ABC transporter C family member 9 OS=Arabidopsis thaliana OX=3702 GN=ABCC9 PE=2 SV=2
CmaG0000717.1	ABC transporter C family member 9 OS=Arabidopsis thaliana OX=3702 GN=ABCC9 PE=2 SV=2
CmaG0016341.1	ABC transporter D family member 1 OS=Arabidopsis thaliana OX=3702 GN=ABCD1 PE=1 SV=1
CmaG0016875.1	ABC transporter D family member 2, chloroplastic OS=Arabidopsis thaliana OX=3702 GN=ABCC2 PE=1 SV=1
CmaG0021307.1	ABC transporter E family member 2 OS=Arabidopsis thaliana OX=3702 GN=ABCE2 PE=2 SV=1
CmaG0011842.1	ABC transporter F family member 1 OS=Arabidopsis thaliana OX=3702 GN=ABCF1 PE=2 SV=1
CmaG0022283.1	ABC transporter F family member 3 OS=Arabidopsis thaliana OX=3702 GN=ABCF3 PE=1 SV=1
CmaG0005202.1	ABC transporter F family member 4 OS=Arabidopsis thaliana OX=3702 GN=ABCF4 PE=2 SV=1
CmaG0005204.1	ABC transporter F family member 4 OS=Arabidopsis thaliana OX=3702 GN=ABCF4 PE=2 SV=1
CmaG0005213.1	ABC transporter F family member 4 OS=Arabidopsis thaliana OX=3702 GN=ABCF4 PE=2 SV=1
CmaG0005214.1	ABC transporter F family member 4 OS=Arabidopsis thaliana OX=3702 GN=ABCF4 PE=2 SV=1
CmaG0005215.1	ABC transporter F family member 4 OS=Arabidopsis thaliana OX=3702 GN=ABCF4 PE=2 SV=1
CmaG0005217.1	ABC transporter F family member 4 OS=Arabidopsis thaliana OX=3702 GN=ABCF4 PE=2 SV=1
CmaG0016469.1	ABC transporter F family member 5 OS=Arabidopsis thaliana OX=3702 GN=ABCF5 PE=2 SV=1
CmaG0027744.1	ABC transporter G family member 1 OS=Arabidopsis thaliana OX=3702 GN=ABCG1 PE=2 SV=1
CmaG0006018.1	ABC transporter G family member 10 OS=Arabidopsis thaliana OX=3702 GN=ABCG10 PE=3 SV=1

CmaG0022485.1	ABC transporter G family member 10 OS=Arabidopsis thaliana OX=3702 GN=ABCG10 PE=3 SV=1
CmaG0003884.1	ABC transporter G family member 11 OS=Arabidopsis thaliana OX=3702 GN=ABCG11 PE=1 SV=1
CmaG0003885.1	ABC transporter G family member 11 OS=Arabidopsis thaliana OX=3702 GN=ABCG11 PE=1 SV=1
CmaG0003886.1	ABC transporter G family member 11 OS=Arabidopsis thaliana OX=3702 GN=ABCG11 PE=1 SV=1
CmaG0003887.1	ABC transporter G family member 11 OS=Arabidopsis thaliana OX=3702 GN=ABCG11 PE=1 SV=1
CmaG0003888.1	ABC transporter G family member 11 OS=Arabidopsis thaliana OX=3702 GN=ABCG11 PE=1 SV=1
CmaG0003889.1	ABC transporter G family member 11 OS=Arabidopsis thaliana OX=3702 GN=ABCG11 PE=1 SV=1
CmaG0003890.1	ABC transporter G family member 11 OS=Arabidopsis thaliana OX=3702 GN=ABCG11 PE=1 SV=1
CmaG0016234.1	ABC transporter G family member 11 OS=Arabidopsis thaliana OX=3702 GN=ABCG11 PE=1 SV=1
CmaG0016268.1	ABC transporter G family member 11 OS=Arabidopsis thaliana OX=3702 GN=ABCG11 PE=1 SV=1
CmaG0016270.1	ABC transporter G family member 11 OS=Arabidopsis thaliana OX=3702 GN=ABCG11 PE=1 SV=1
CmaG0016271.1	ABC transporter G family member 11 OS=Arabidopsis thaliana OX=3702 GN=ABCG11 PE=1 SV=1
CmaG0016941.1	ABC transporter G family member 11 OS=Arabidopsis thaliana OX=3702 GN=ABCG11 PE=1 SV=1
CmaG0005308.1	ABC transporter G family member 14 OS=Arabidopsis thaliana OX=3702 GN=ABCG14 PE=2 SV=1
CmaG0021315.1	ABC transporter G family member 14 OS=Arabidopsis thaliana OX=3702 GN=ABCG14 PE=2 SV=1
CmaG0011527.1	ABC transporter G family member 15 OS=Arabidopsis thaliana OX=3702 GN=ABCG15 PE=2 SV=2
CmaG0016269.1	ABC transporter G family member 15 OS=Arabidopsis thaliana OX=3702 GN=ABCG15 PE=2 SV=2
CmaG0009760.1	ABC transporter G family member 18 OS=Arabidopsis thaliana OX=3702 GN=ABCG18 PE=2 SV=1
CmaG0012562.1	ABC transporter G family member 19 OS=Arabidopsis thaliana OX=3702 GN=ABCG19 PE=1 SV=1
CmaG0009164.1	ABC transporter G family member 20 OS=Arabidopsis thaliana OX=3702 GN=ABCG20 PE=2 SV=1
CmaG0019180.1	ABC transporter G family member 21 OS=Arabidopsis thaliana OX=3702 GN=ABCG21 PE=2 SV=2
CmaG0008315.1	ABC transporter G family member 22 OS=Arabidopsis thaliana OX=3702 GN=ABCG22 PE=1 SV=1
CmaG0017639.1	ABC transporter G family member 22 OS=Arabidopsis thaliana OX=3702 GN=ABCG22 PE=1 SV=1
CmaG0011461.1	ABC transporter G family member 23 OS=Arabidopsis thaliana OX=3702 GN=ABCG23 PE=2 SV=1
CmaG0027633.1	ABC transporter G family member 24 OS=Arabidopsis thaliana OX=3702 GN=ABCG24 PE=2 SV=2
CmaG0025066.1	ABC transporter G family member 25 OS=Arabidopsis thaliana OX=3702 GN=ABCG25 PE=2 SV=1
CmaG0023058.1	ABC transporter G family member 26 OS=Arabidopsis thaliana OX=3702 GN=ABCG26 PE=3 SV=2
CmaG0025842.1	ABC transporter G family member 28 OS=Arabidopsis thaliana OX=3702 GN=ABCG28 PE=3 SV=1
CmaG0011836.1	ABC transporter G family member 3 OS=Arabidopsis thaliana OX=3702 GN=ABCG3 PE=1 SV=2
CmaG0012657.1	ABC transporter G family member 31 OS=Arabidopsis thaliana OX=3702 GN=ABCG31 PE=1 SV=1
CmaG0026470.1	ABC transporter G family member 31 OS=Arabidopsis thaliana OX=3702 GN=ABCG31 PE=1 SV=1
CmaG0023660.1	ABC transporter G family member 32 OS=Arabidopsis thaliana OX=3702 GN=ABCG32 PE=1 SV=1
CmaG0007754.1	ABC transporter G family member 34 OS=Arabidopsis thaliana OX=3702 GN=ABCG34 PE=2 SV=1
CmaG0007238.1	ABC transporter G family member 36 OS=Arabidopsis thaliana OX=3702 GN=ABCG36 PE=1 SV=1
CmaG0023666.1	ABC transporter G family member 38 OS=Arabidopsis thaliana OX=3702 GN=ABCG38 PE=2 SV=1
CmaG0009939.1	ABC transporter G family member 39 OS=Arabidopsis thaliana OX=3702 GN=ABCG39 PE=3 SV=1
CmaG0009940.1	ABC transporter G family member 39 OS=Arabidopsis thaliana OX=3702 GN=ABCG39 PE=3 SV=1
CmaG0009943.1	ABC transporter G family member 39 OS=Arabidopsis thaliana OX=3702 GN=ABCG39 PE=3 SV=1
CmaG0002908.1	ABC transporter G family member 41 OS=Oryza sativa subsp. japonica OX=39947 GN=ABCG41 PE=3 SV=1
CmaG0007239.1	ABC transporter G family member 42 OS=Oryza sativa subsp. japonica OX=39947 GN=ABCG42 PE=2 SV=1
CmaG0007240.1	ABC transporter G family member 42 OS=Oryza sativa subsp. japonica OX=39947 GN=ABCG42 PE=2 SV=1
CmaG0016689.1	ABC transporter G family member 5 OS=Arabidopsis thaliana OX=3702 GN=ABCG5 PE=2 SV=1
CmaG0012561.1	ABC transporter G family member 6 OS=Arabidopsis thaliana OX=3702 GN=ABCG6 PE=2 SV=1
CmaG0018320.1	ABC transporter G family member 7 OS=Arabidopsis thaliana OX=3702 GN=ABCG7 PE=2 SV=1
CmaG0002092.1	ABC transporter G family member 8 OS=Arabidopsis thaliana OX=3702 GN=ABCG8 PE=2 SV=1
CmaG0022618.1	ABC transporter G family member 9 OS=Arabidopsis thaliana OX=3702 GN=ABCG9 PE=3 SV=2

CmaG0022619.1	ABC transporter G family member 9 OS=Arabidopsis thaliana OX=3702 GN=ABCG9 PE=3 SV=2
CmaG0010080.1	ABC transporter I family member 1 OS=Arabidopsis thaliana OX=3702 GN=ABCI1 PE=2 SV=1
CmaG0016648.1	ABC transporter I family member 10 OS=Arabidopsis thaliana OX=3702 GN=ABCI10 PE=2 SV=1
CmaG0015622.1	ABC transporter I family member 11, chloroplastic OS=Arabidopsis thaliana OX=3702 GN=ABCI11 PE=2 SV=1
CmaG0009878.1	ABC transporter I family member 17 OS=Arabidopsis thaliana OX=3702 GN=ABCI17 PE=2 SV=1
CmaG0009894.1	ABC transporter I family member 17 OS=Arabidopsis thaliana OX=3702 GN=ABCI17 PE=2 SV=1
CmaG0016037.1	ABC transporter I family member 19 OS=Arabidopsis thaliana OX=3702 GN=ABCI19 PE=2 SV=1
CmaG0012669.1	ABC transporter I family member 20 OS=Arabidopsis thaliana OX=3702 GN=ABCI20 PE=2 SV=1
CmaG0000175.1	ABC transporter I family member 6, chloroplastic OS=Arabidopsis thaliana OX=3702 GN=ABCI6 PE=1 SV=1
CmaG0013856.1	Protein ABC transporter 1, mitochondrial OS=Arabidopsis thaliana OX=3702 GN=ABC1 PE=2 SV=1
CmaG0023494.1	Putative ABC transporter B family member 8 OS=Arabidopsis thaliana OX=3702 GN=ABCB8 PE=5 SV=1
Membrane-bound O-acyltransferase	
CmaG0019850.1	Putative membrane-bound O-acyltransferase C24H6.01c OS=Schizosaccharomyces pombe (strain 972 / ATCC 24843) OX=284812 GN=SPAC24H6.01c PE=3 SV=4
Acyl CoA binding protein	
CmaG0002266.1	Acyl-CoA-binding domain-containing protein 2 OS=Arabidopsis thaliana OX=3702 GN=ACBP2 PE=1 SV=1
CmaG0006640.1	Acyl-CoA-binding domain-containing protein 3 OS=Arabidopsis thaliana OX=3702 GN=ACBP3 PE=1 SV=1
CmaG0025271.1	Acyl-CoA-binding domain-containing protein 3 OS=Arabidopsis thaliana OX=3702 GN=ACBP3 PE=1 SV=1
CmaG0009437.1	Acyl-CoA-binding domain-containing protein 4 OS=Arabidopsis thaliana OX=3702 GN=ACBP4 PE=1 SV=1
CmaG0012809.1	Acyl-CoA-binding domain-containing protein 4 OS=Arabidopsis thaliana OX=3702 GN=ACBP4 PE=1 SV=1
CmaG0027511.1	Acyl-CoA-binding domain-containing protein 4 OS=Arabidopsis thaliana OX=3702 GN=ACBP4 PE=1 SV=1
CmaG0003400.1	Acyl-CoA-binding domain-containing protein 6 OS=Oryza sativa subsp. japonica OX=39947 GN=ACBP6 PE=2 SV=1
CmaG0023302.1	Acyl-CoA-binding protein OS=Fritillaria agrestis OX=64177 GN=ACABP PE=3 SV=1
CmaG0020974.1	Acyl-CoA-binding protein OS=Ricinus communis OX=3988 PE=3 SV=1
Acyl carrier protein	
CmaG0003188.1	Acyl carrier protein 1, chloroplastic OS=Casuarina glauca OX=3522 GN=ACP1 PE=2 SV=1
CmaG0007493.1	Acyl carrier protein 1, chloroplastic OS=Casuarina glauca OX=3522 GN=ACP1 PE=2 SV=1
CmaG0019910.1	Acyl carrier protein 1, chloroplastic OS=Casuarina glauca OX=3522 GN=ACP1 PE=2 SV=1
CmaG0000761.1	Acyl carrier protein 1, mitochondrial OS=Arabidopsis thaliana OX=3702 GN=MTACP1 PE=1 SV=1
CmaG0007784.1	Acyl carrier protein 2, mitochondrial OS=Arabidopsis thaliana OX=3702 GN=MTACP2 PE=1 SV=1
CmaG0005845.1	Acyl carrier protein 3, mitochondrial OS=Arabidopsis thaliana OX=3702 GN=MTACP2 PE=1 SV=1
Acyl-CoA thioesterase	
CmaG0010174.1	Acyl-coenzyme A thioesterase 13 OS=Mus musculus OX=10090 GN=Acot13 PE=1 SV=1
CmaG0006514.1	Acyl-coenzyme A thioesterase 13 OS=Pongo abelii OX=9601 GN=ACOT13 PE=2 SV=1
CmaG0026394.1	Acyl-coenzyme A thioesterase 13 OS=Pongo abelii OX=9601 GN=ACOT13 PE=2 SV=1
CmaG0006852.1	Acyl-coenzyme A thioesterase 8 OS=Mus musculus OX=10090 GN=Acot8 PE=1 SV=1
CmaG0000056.1	Acyl-coenzyme A thioesterase 9, mitochondrial OS=Bos taurus OX=9913 GN=ACOT9 PE=2 SV=1
CmaG0018462.1	Acyl-coenzyme A thioesterase 9, mitochondrial OS=Homo sapiens OX=9606 GN=ACOT9 PE=1 SV=2
Acyl-CoA oxidase	
CmaG0016361.1	Acyl-coenzyme A oxidase 2, peroxisomal OS=Arabidopsis thaliana OX=3702 GN=ACX2 PE=1 SV=2
CmaG0006080.1	Acyl-coenzyme A oxidase 3, peroxisomal OS=Arabidopsis thaliana OX=3702 GN=ACX3 PE=1 SV=1
CmaG0008816.1	Acyl-coenzyme A oxidase 4, peroxisomal OS=Arabidopsis thaliana OX=3702 GN=ACX4 PE=1 SV=1
CmaG0027148.1	Acyl-coenzyme A oxidase 4, peroxisomal OS=Arabidopsis thaliana OX=3702 GN=ACX4 PE=1 SV=1
CmaG0020866.1	Peroxisomal acyl-coenzyme A oxidase 1 OS=Arabidopsis thaliana OX=3702 GN=ACX1 PE=1 SV=1

Acylhydrolase (DAD1-like)	
CmaG0005567.1	Probable dolichyl-diphosphooligosaccharide--protein glycosyltransferase subunit 3B OS=Arabidopsis thaliana OX=3702 GN=OST3B PE=2 SV=1
CmaG0005568.1	Probable dolichyl-diphosphooligosaccharide--protein glycosyltransferase subunit 3B OS=Arabidopsis thaliana OX=3702 GN=OST3B PE=2 SV=1
CmaG0001243.1	Dolichyl-diphosphooligosaccharide--protein glycosyltransferase 48 kDa subunit OS=Oryza sativa subsp. japonica OX=39947 GN=OST48 PE=2 SV=1
CmaG0024728.1	Dolichyl-diphosphooligosaccharide--protein glycosyltransferase subunit 1A OS=Arabidopsis thaliana OX=3702 GN=OST1A PE=2 SV=1
CmaG0027649.1	Dolichyl-diphosphooligosaccharide--protein glycosyltransferase subunit 1B OS=Arabidopsis thaliana OX=3702 GN=OST1B PE=1 SV=1
CmaG0010810.1	Dolichyl-diphosphooligosaccharide--protein glycosyltransferase subunit 2 OS=Arabidopsis thaliana OX=3702 GN=RPN2 PE=1 SV=1
CmaG0023519.1	Dolichyl-diphosphooligosaccharide--protein glycosyltransferase subunit STT3A OS=Arabidopsis thaliana OX=3702 GN=STT3A PE=2 SV=1
CmaG0016958.1	Dolichyl-diphosphooligosaccharide--protein glycosyltransferase subunit STT3B OS=Arabidopsis thaliana OX=3702 GN=STT3B PE=2 SV=1
Alcohol-forming fatty Acyl-CoA reductase	
CmaG0008248.1	Fatty acyl-CoA reductase 2 OS=Arabidopsis thaliana OX=3702 GN=FAR2 PE=1 SV=2
CmaG0011245.1	Fatty acyl-CoA reductase 3 OS=Arabidopsis thaliana OX=3702 GN=FAR3 PE=1 SV=1
CmaG0011246.1	Fatty acyl-CoA reductase 3 OS=Arabidopsis thaliana OX=3702 GN=FAR3 PE=1 SV=1
CmaG0011249.1	Fatty acyl-CoA reductase 3 OS=Arabidopsis thaliana OX=3702 GN=FAR3 PE=1 SV=1
CmaG0011250.1	Fatty acyl-CoA reductase 3 OS=Arabidopsis thaliana OX=3702 GN=FAR3 PE=1 SV=1
CmaG0011253.1	Fatty acyl-CoA reductase 3 OS=Arabidopsis thaliana OX=3702 GN=FAR3 PE=1 SV=1
CmaG0011254.1	Fatty acyl-CoA reductase 3 OS=Arabidopsis thaliana OX=3702 GN=FAR3 PE=1 SV=1
CmaG0011266.1	Fatty acyl-CoA reductase 3 OS=Arabidopsis thaliana OX=3702 GN=FAR3 PE=1 SV=1
CmaG0011267.1	Fatty acyl-CoA reductase 3 OS=Arabidopsis thaliana OX=3702 GN=FAR3 PE=1 SV=1
CmaG0011819.1	Fatty acyl-CoA reductase 3 OS=Arabidopsis thaliana OX=3702 GN=FAR3 PE=1 SV=1
CmaG0011820.1	Fatty acyl-CoA reductase 3 OS=Arabidopsis thaliana OX=3702 GN=FAR3 PE=1 SV=1
CmaG0011823.1	Fatty acyl-CoA reductase 3 OS=Arabidopsis thaliana OX=3702 GN=FAR3 PE=1 SV=1
CmaG0012200.1	Fatty acyl-CoA reductase 3 OS=Arabidopsis thaliana OX=3702 GN=FAR3 PE=1 SV=1
Allene oxide cylase	
CmaG0014699.1	Allene oxide cyclase, chloroplastic OS=Oryza sativa subsp. japonica OX=39947 GN=AOC PE=1 SV=1
CmaG0014700.1	Allene oxide cyclase, chloroplastic OS=Oryza sativa subsp. japonica OX=39947 GN=AOC PE=1 SV=1
CmaG0014701.1	Allene oxide cyclase, chloroplastic OS=Oryza sativa subsp. japonica OX=39947 GN=AOC PE=1 SV=1
CmaG0014702.1	Allene oxide cyclase, chloroplastic OS=Oryza sativa subsp. japonica OX=39947 GN=AOC PE=1 SV=1
Allene oxide synthase	
CmaG0001129.1	Allene oxide synthase 1, chloroplastic OS=Solanum lycopersicum OX=4081 GN=AOS1 PE=2 SV=1
CmaG0017781.1	Allene oxide synthase 1, chloroplastic OS=Solanum lycopersicum OX=4081 GN=AOS1 PE=2 SV=1
CmaG0017782.1	Allene oxide synthase 1, chloroplastic OS=Solanum lycopersicum OX=4081 GN=AOS1 PE=2 SV=1
CmaG0001209.1	Allene oxide synthase 3 OS=Solanum lycopersicum OX=4081 GN=AOS3 PE=1 SV=1
CmaG0001210.1	Allene oxide synthase 3 OS=Solanum lycopersicum OX=4081 GN=AOS3 PE=1 SV=1
CmaG0001211.1	Allene oxide synthase 3 OS=Solanum lycopersicum OX=4081 GN=AOS3 PE=1 SV=1
BAHD acyltransferase	
CmaG0009705.1	BAHD acyltransferase DCR OS=Arabidopsis thaliana OX=3702 GN=DCR PE=2 SV=1
CmaG0028040.1	BAHD acyltransferase DCR OS=Arabidopsis thaliana OX=3702 GN=DCR PE=2 SV=1
CmaG0028395.1	BAHD acyltransferase DCR OS=Arabidopsis thaliana OX=3702 GN=DCR PE=2 SV=1
CmaG0028409.1	BAHD acyltransferase DCR OS=Arabidopsis thaliana OX=3702 GN=DCR PE=2 SV=1

Biotin carboxylase	
CmaG0004574.1	Biotin carboxylase 1, chloroplastic OS=Populus trichocarpa OX=3694 GN=POPTRDRAFT_831870 PE=2 SV=1
CmaG0009912.1	Biotin carboxylase 1, chloroplastic OS=Populus trichocarpa OX=3694 GN=POPTRDRAFT_831870 PE=2 SV=1
Biotin carboxyl carrier protein	
CmaG0015113.1	Biotin carboxyl carrier protein of acetyl-CoA carboxylase 2, chloroplastic OS=Arabidopsis thaliana OX=3702 GN=BCCP2 PE=1 SV=1
CmaG0027215.1	Biotin carboxyl carrier protein of acetyl-CoA carboxylase OS=Nostoc sp. (strain PCC 7120 / SAG 25.82 / UTEX 2576) OX=103690 GN=accB PE=1 SV=2
CmaG0028138.1	Biotin carboxyl carrier protein of acetyl-CoA carboxylase OS=Nostoc sp. (strain PCC 7120 / SAG 25.82 / UTEX 2576) OX=103690 GN=accB PE=1 SV=2
CmaG0013233.1	Biotin carboxyl carrier protein of acetyl-CoA carboxylase, chloroplastic OS=Glycine max OX=3847 GN=ACCB-1 PE=1 SV=1
alpha-CT	
CmaG0007514.1	Acetyl-coenzyme A carboxylase carboxyl transferase subunit alpha, chloroplastic OS=Arabidopsis thaliana OX=3702 GN=CAC3 PE=1 SV=1
CmaG0008900.1	Acetyl-coenzyme A carboxylase carboxyl transferase subunit alpha, chloroplastic OS=Arabidopsis thaliana OX=3702 GN=CAC3 PE=1 SV=1
CmaG0027017.1	Acetyl-coenzyme A carboxylase carboxyl transferase subunit alpha, chloroplastic OS=Arabidopsis thaliana OX=3702 GN=CAC3 PE=1 SV=1
beta-CT	
CmaG0001051.1	Acetyl-coenzyme A carboxylase carboxyl transferase subunit beta, chloroplastic OS=Morus indica OX=248361 GN=accD PE=3 SV=1
Caffeoyl-CoA O-Methyltransferase	
CmaG0004453.1	Caffeoyl-CoA O-methyltransferase 1 OS=Populus trichocarpa OX=3694 GN=CCOAOMT1 PE=2 SV=1
CmaG0004454.1	Caffeoyl-CoA O-methyltransferase 1 OS=Populus trichocarpa OX=3694 GN=CCOAOMT1 PE=2 SV=1
CmaG0004455.1	Caffeoyl-CoA O-methyltransferase 1 OS=Populus trichocarpa OX=3694 GN=CCOAOMT1 PE=2 SV=1
CmaG0023415.1	Probable caffeoyl-CoA O-methyltransferase At4g26220 OS=Arabidopsis thaliana OX=3702 GN=At4g26220 PE=2 SV=1
CmaG0023419.1	Probable caffeoyl-CoA O-methyltransferase At4g26220 OS=Arabidopsis thaliana OX=3702 GN=At4g26220 PE=2 SV=1
CmaG0023420.1	Probable caffeoyl-CoA O-methyltransferase At4g26220 OS=Arabidopsis thaliana OX=3702 GN=At4g26220 PE=2 SV=1
CmaG0023421.1	Probable caffeoyl-CoA O-methyltransferase At4g26220 OS=Arabidopsis thaliana OX=3702 GN=At4g26220 PE=2 SV=1
Choline-Phosphate cytidylyltransferase	
CmaG0004908.1	Choline-phosphate cytidylyltransferase 1 OS=Arabidopsis thaliana OX=3702 GN=CCT1 PE=1 SV=1
CmaG0014156.1	Choline-phosphate cytidylyltransferase 1 OS=Arabidopsis thaliana OX=3702 GN=CCT1 PE=1 SV=1
Ceramidase	
CmaG0026382.1	Neutral ceramidase 1 OS=Arabidopsis thaliana OX=3702 GN=NCER1 PE=2 SV=1
CmaG0008908.1	Neutral ceramidase 2 OS=Arabidopsis thaliana OX=3702 GN=NCER2 PE=3 SV=1
CmaG0024819.1	Neutral ceramidase 2 OS=Arabidopsis thaliana OX=3702 GN=NCER2 PE=3 SV=1
CmaG0004540.1	Non-lysosomal glucosylceramidase OS=Mus musculus OX=10090 GN=Gba2 PE=1 SV=2
CmaG0004568.1	Non-lysosomal glucosylceramidase OS=Mus musculus OX=10090 GN=Gba2 PE=1 SV=2
CmaG0014522.1	Non-lysosomal glucosylceramidase OS=Mus musculus OX=10090 GN=Gba2 PE=1 SV=2
CmaG0018952.1	Non-lysosomal glucosylceramidase OS=Mus musculus OX=10090 GN=Gba2 PE=1 SV=2
CmaG0005755.1	Alkaline ceramidase OS=Arabidopsis thaliana OX=3702 GN=ACER PE=2 SV=1
CmaG0018996.1	Alkaline ceramidase OS=Arabidopsis thaliana OX=3702 GN=ACER PE=2 SV=1
Ceramide kinase	
CmaG0025402.1	Ceramide kinase OS=Arabidopsis thaliana OX=3702 GN=CERK PE=1 SV=1

Choline kinase	
CmaG0017543.1	Probable choline kinase 1 OS=Arabidopsis thaliana OX=3702 GN=CK1 PE=2 SV=1
CmaG0000666.1	Probable choline kinase 2 OS=Arabidopsis thaliana OX=3702 GN=At1g74320 PE=2 SV=1
Cardiolipin synthase	
CmaG0005587.1	Cardiolipin synthase (CMP-forming), mitochondrial OS=Arabidopsis thaliana OX=3702 GN=CLS PE=1 SV=1
Diacylglycerol cholinephosphotransferase	
CmaG0019210.1	Choline/ethanolaminephosphotransferase 1 OS=Arabidopsis thaliana OX=3702 GN=AAPT1 PE=1 SV=1
Diacylglycerol kinase	
CmaG0001690.1	Diacylglycerol kinase 1 OS=Arabidopsis thaliana OX=3702 GN=DGK1 PE=1 SV=2
CmaG0001691.1	Diacylglycerol kinase 1 OS=Arabidopsis thaliana OX=3702 GN=DGK1 PE=1 SV=2
CmaG0020637.1	Diacylglycerol kinase 1 OS=Arabidopsis thaliana OX=3702 GN=DGK1 PE=1 SV=2
CmaG0023944.1	Diacylglycerol kinase 2 OS=Arabidopsis thaliana OX=3702 GN=DGK2 PE=1 SV=1
CmaG0025446.1	Diacylglycerol kinase 3 OS=Arabidopsis thaliana OX=3702 GN=DGK3 PE=2 SV=1
CmaG0025447.1	Diacylglycerol kinase 4 OS=Arabidopsis thaliana OX=3702 GN=DGK4 PE=2 SV=1
CmaG0022681.1	Diacylglycerol kinase 5 OS=Arabidopsis thaliana OX=3702 GN=DGK5 PE=2 SV=1
CmaG0023183.1	Diacylglycerol kinase 5 OS=Arabidopsis thaliana OX=3702 GN=DGK5 PE=2 SV=1
CmaG0026763.1	Diacylglycerol kinase 5 OS=Arabidopsis thaliana OX=3702 GN=DGK5 PE=2 SV=1
CmaG0022685.1	Diacylglycerol kinase 6 OS=Arabidopsis thaliana OX=3702 GN=DGK6 PE=3 SV=1
Dienoyl CoA isomerase	
CmaG0019891.1	Delta(3,5)-Delta(2,4)-dienoyl-CoA isomerase, peroxisomal OS=Arabidopsis thaliana OX=3702 GN=DCI1 PE=2 SV=1
Acyl-CoA: diacylglycerol acyltransferase	
CmaG0023347.1	Diacylglycerol O-acyltransferase 1 OS=Corylus americana OX=78632 GN=DGAT1 PE=1 SV=1
CmaG0012213.1	Diacylglycerol O-acyltransferase 2D OS=Glycine max OX=3847 GN=DGAT2D PE=1 SV=1
CmaG0002878.1	Diacylglycerol O-acyltransferase 3 OS=Arabidopsis thaliana OX=3702 GN=DGAT3 PE=1 SV=2
Digalactosyldiacylglycerol synthase	
CmaG0008385.1	Digalactosyldiacylglycerol synthase 1, chloroplastic OS=Lotus japonicus OX=34305 GN=DGD1 PE=2 SV=1
CmaG0006511.1	Digalactosyldiacylglycerol synthase 2, chloroplastic OS=Lotus japonicus OX=34305 GN=DGD2 PE=2 SV=1
Disease resistance-responsive family protein / dirigent protein	
CmaG0010360.1	Dirigent protein 1 OS=Arabidopsis thaliana OX=3702 GN=DIR1 PE=2 SV=1
CmaG0010362.1	Dirigent protein 1 OS=Arabidopsis thaliana OX=3702 GN=DIR1 PE=2 SV=1
CmaG0010367.1	Dirigent protein 1 OS=Arabidopsis thaliana OX=3702 GN=DIR1 PE=2 SV=1
CmaG0016637.1	Dirigent protein 10 OS=Arabidopsis thaliana OX=3702 GN=DIR10 PE=2 SV=1
CmaG0003457.1	Dirigent protein 11 OS=Arabidopsis thaliana OX=3702 GN=DIR11 PE=2 SV=1
CmaG0017774.1	Dirigent protein 11 OS=Arabidopsis thaliana OX=3702 GN=DIR11 PE=2 SV=1
CmaG0018895.1	Dirigent protein 16 OS=Arabidopsis thaliana OX=3702 GN=DIR16 PE=2 SV=1
CmaG0018896.1	Dirigent protein 16 OS=Arabidopsis thaliana OX=3702 GN=DIR16 PE=2 SV=1
CmaG0009177.1	Dirigent protein 17 OS=Arabidopsis thaliana OX=3702 GN=DIR17 PE=2 SV=1
CmaG0008231.1	Dirigent protein 19 OS=Arabidopsis thaliana OX=3702 GN=DIR19 PE=2 SV=1
CmaG0008234.1	Dirigent protein 19 OS=Arabidopsis thaliana OX=3702 GN=DIR19 PE=2 SV=1

CmaG0008235.1	Dirigent protein 19 OS=Arabidopsis thaliana OX=3702 GN=DIR19 PE=2 SV=1
CmaG0011728.1	Dirigent protein 19 OS=Arabidopsis thaliana OX=3702 GN=DIR19 PE=2 SV=1
CmaG0011729.1	Dirigent protein 19 OS=Arabidopsis thaliana OX=3702 GN=DIR19 PE=2 SV=1
CmaG0010373.1	Dirigent protein 2 OS=Arabidopsis thaliana OX=3702 GN=DIR2 PE=2 SV=1
CmaG0001463.1	Dirigent protein 20 OS=Arabidopsis thaliana OX=3702 GN=DIR20 PE=2 SV=1
CmaG0011726.1	Dirigent protein 20 OS=Arabidopsis thaliana OX=3702 GN=DIR20 PE=2 SV=1
CmaG0011730.1	Dirigent protein 20 OS=Arabidopsis thaliana OX=3702 GN=DIR20 PE=2 SV=1
CmaG0000556.1	Dirigent protein 21 OS=Arabidopsis thaliana OX=3702 GN=DIR21 PE=3 SV=1
CmaG0008230.1	Dirigent protein 21 OS=Arabidopsis thaliana OX=3702 GN=DIR21 PE=3 SV=1
CmaG0011727.1	Dirigent protein 21 OS=Arabidopsis thaliana OX=3702 GN=DIR21 PE=3 SV=1
CmaG0000558.1	Dirigent protein 23 OS=Arabidopsis thaliana OX=3702 GN=DIR23 PE=2 SV=1
CmaG0001458.1	Dirigent protein 23 OS=Arabidopsis thaliana OX=3702 GN=DIR23 PE=2 SV=1
CmaG0001462.1	Dirigent protein 23 OS=Arabidopsis thaliana OX=3702 GN=DIR23 PE=2 SV=1
CmaG0001464.1	Dirigent protein 23 OS=Arabidopsis thaliana OX=3702 GN=DIR23 PE=2 SV=1
CmaG0010386.1	Dirigent protein 23 OS=Arabidopsis thaliana OX=3702 GN=DIR23 PE=2 SV=1
CmaG0012266.1	Dirigent protein 23 OS=Arabidopsis thaliana OX=3702 GN=DIR23 PE=2 SV=1
CmaG0026121.1	Dirigent protein 25 OS=Arabidopsis thaliana OX=3702 GN=DIR25 PE=3 SV=1
CmaG0027758.1	Dirigent protein 25 OS=Arabidopsis thaliana OX=3702 GN=DIR25 PE=3 SV=1
CmaG0027760.1	Dirigent protein 25 OS=Arabidopsis thaliana OX=3702 GN=DIR25 PE=3 SV=1
CmaG0008237.1	Dirigent protein 3 OS=Arabidopsis thaliana OX=3702 GN=DIR3 PE=3 SV=1
CmaG0008238.1	Dirigent protein 3 OS=Arabidopsis thaliana OX=3702 GN=DIR3 PE=3 SV=1
CmaG0010352.1	Dirigent protein 4 OS=Arabidopsis thaliana OX=3702 GN=DIR4 PE=2 SV=1
CmaG0024420.1	Dirigent protein 4 OS=Arabidopsis thaliana OX=3702 GN=DIR4 PE=2 SV=1
CmaG0021847.1	Dirigent protein 5 OS=Arabidopsis thaliana OX=3702 GN=DIR5 PE=2 SV=1
CmaG0011725.1	Dirigent protein 7 OS=Arabidopsis thaliana OX=3702 GN=DIR7 PE=2 SV=1
CmaG0027759.1	Dirigent protein 9 OS=Arabidopsis thaliana OX=3702 GN=DIR9 PE=2 SV=1
CmaG0000094.1	Putative lipid-transfer protein DIR1 OS=Arabidopsis thaliana OX=3702 GN=DIR1 PE=1 SV=1
CmaG0001242.1	Putative lipid-transfer protein DIR1 OS=Arabidopsis thaliana OX=3702 GN=DIR1 PE=1 SV=1
CmaG0018361.1	Putative lipid-transfer protein DIR1 OS=Arabidopsis thaliana OX=3702 GN=DIR1 PE=1 SV=1
CmaG0027987.1	Putative lipid-transfer protein DIR1 OS=Arabidopsis thaliana OX=3702 GN=DIR1 PE=1 SV=1
Peroxisomal enoyl-CoA hydratase	
CmaG0018637.1	Enoyl-CoA delta isomerase 2, peroxisomal OS=Arabidopsis thaliana OX=3702 GN=ECI2 PE=1 SV=1
CmaG0024826.1	Enoyl-CoA hydratase 2, peroxisomal OS=Arabidopsis thaliana OX=3702 GN=ECH2 PE=1 SV=1
CmaG0024827.1	Enoyl-CoA hydratase 2, peroxisomal OS=Arabidopsis thaliana OX=3702 GN=ECH2 PE=1 SV=1
CmaG0005499.1	Probable enoyl-CoA hydratase 1, peroxisomal OS=Arabidopsis thaliana OX=3702 GN=ECHIA PE=1 SV=1
CmaG0021471.1	Probable enoyl-CoA hydratase 1, peroxisomal OS=Arabidopsis thaliana OX=3702 GN=ECHIA PE=1 SV=1
CmaG0020887.1	Probable enoyl-CoA hydratase 2, mitochondrial OS=Arabidopsis thaliana OX=3702 GN=At4g16800 PE=2 SV=1
CmaG0026011.1	(R)-specific enoyl-CoA hydratase OS=Aeromonas caviae OX=648 GN=phaJ PE=1 SV=1
Enoyl-CoA reductase	
CmaG0004818.1	Peroxisomal 2,4-dienoyl-CoA reductase OS=Arabidopsis thaliana OX=3702 GN=At3g12800 PE=2 SV=1
CmaG0020742.1	Peroxisomal 2,4-dienoyl-CoA reductase OS=Arabidopsis thaliana OX=3702 GN=At3g12800 PE=2 SV=1
Ethanolamine kinase	
CmaG0023722.1	Probable ethanolamine kinase OS=Arabidopsis thaliana OX=3702 GN=EMB1187 PE=2 SV=1

Fatty acid amide hydrolase	
CmaG0004082.1	Fatty acid amide hydrolase OS=Arabidopsis thaliana OX=3702 GN=FAAH PE=1 SV=1
CmaG0024021.1	Fatty acid amide hydrolase OS=Arabidopsis thaliana OX=3702 GN=FAAH PE=1 SV=1
CmaG0008189.1	Omega-6 fatty acid desaturase, endoplasmic reticulum isozyme 2 OS=Glycine max OX=3847 GN=FAD2-2 PE=2 SV=1
FAD3	
CmaG0016572.1	Omega-3 fatty acid desaturase, endoplasmic reticulum OS=Glycine max OX=3847 GN=FAD3 PE=2 SV=1
CmaG0021988.1	Omega-3 fatty acid desaturase, endoplasmic reticulum OS=Glycine max OX=3847 GN=FAD3 PE=2 SV=1
CmaG0026493.1	Omega-3 fatty acid desaturase, endoplasmic reticulum OS=Nicotiana tabacum OX=4097 GN=FAD3 PE=2 SV=1
FAD7	
CmaG0028086.1	Omega-3 fatty acid desaturase, chloroplastic OS=Ricinus communis OX=3988 GN=FAD7A-1 PE=2 SV=1
CmaG0008819.1	Omega-3 fatty acid desaturase, chloroplastic OS=Sesamum indicum OX=4182 GN=FAD7 PE=2 SV=1
Fatty acyl 2-hydroxylase FAH	
CmaG0024104.1	Dihydroceramide fatty acyl 2-hydroxylase FAH1 OS=Arabidopsis thaliana OX=3702 GN=FAH1 PE=1 SV=1
Palmitoyl-acyl carrier protein thioesterase	
CmaG0002799.1	Palmitoyl-acyl carrier protein thioesterase, chloroplastic OS=Arabidopsis thaliana OX=3702 GN=FATB PE=1 SV=1
CmaG0020078.1	Palmitoyl-acyl carrier protein thioesterase, chloroplastic OS=Arabidopsis thaliana OX=3702 GN=FATB PE=1 SV=1
CmaG0027150.1	Palmitoyl-acyl carrier protein thioesterase, chloroplastic OS=Arabidopsis thaliana OX=3702 GN=FATB PE=1 SV=1
CmaG0023654.1	Palmitoyl-acyl carrier protein thioesterase, chloroplastic OS=Gossypium hirsutum OX=3635 GN=FATB1 PE=1 SV=1
CmaG0027149.1	Palmitoyl-acyl carrier protein thioesterase, chloroplastic OS=Gossypium hirsutum OX=3635 GN=FATB1 PE=1 SV=1
CmaG0027152.1	Palmitoyl-acyl carrier protein thioesterase, chloroplastic OS=Gossypium hirsutum OX=3635 GN=FATB1 PE=1 SV=1
CmaG0006304.1	Oleoyl-acyl carrier protein thioesterase 1, chloroplastic OS=Arabidopsis thaliana OX=3702 GN=FATA PE=1 SV=1
Feruloyl transferase	
CmaG0007284.1	Omega-hydroxypalmitate O-feruloyl transferase OS=Arabidopsis thaliana OX=3702 GN=HHT1 PE=1 SV=1
CmaG0013251.1	Omega-hydroxypalmitate O-feruloyl transferase OS=Arabidopsis thaliana OX=3702 GN=HHT1 PE=1 SV=1
CmaG0015631.1	Omega-hydroxypalmitate O-feruloyl transferase OS=Arabidopsis thaliana OX=3702 GN=HHT1 PE=1 SV=1
CmaG0015840.1	Omega-hydroxypalmitate O-feruloyl transferase OS=Arabidopsis thaliana OX=3702 GN=HHT1 PE=1 SV=1
CmaG0020067.1	Omega-hydroxypalmitate O-feruloyl transferase OS=Arabidopsis thaliana OX=3702 GN=HHT1 PE=1 SV=1
CmaG0023650.1	Omega-hydroxypalmitate O-feruloyl transferase OS=Arabidopsis thaliana OX=3702 GN=HHT1 PE=1 SV=1
CmaG0028101.1	Omega-hydroxypalmitate O-feruloyl transferase OS=Arabidopsis thaliana OX=3702 GN=HHT1 PE=1 SV=1
Glycerol kinase	
CmaG0026946.1	Glycerol kinase OS=Arabidopsis thaliana OX=3702 GN=GLPK PE=1 SV=1
CmaG0026950.1	Glycerol kinase OS=Arabidopsis thaliana OX=3702 GN=GLPK PE=1 SV=1
CmaG0026952.1	Glycerol kinase OS=Arabidopsis thaliana OX=3702 GN=GLPK PE=1 SV=1
CmaG0026955.1	Glycerol kinase OS=Arabidopsis thaliana OX=3702 GN=GLPK PE=1 SV=1
CmaG0026963.1	Glycerol kinase OS=Arabidopsis thaliana OX=3702 GN=GLPK PE=1 SV=1
CmaG0027291.1	Glycerol kinase OS=Arabidopsis thaliana OX=3702 GN=GLPK PE=1 SV=1
CmaG0027293.1	Glycerol kinase OS=Arabidopsis thaliana OX=3702 GN=GLPK PE=1 SV=1
CmaG0027296.1	Glycerol kinase OS=Arabidopsis thaliana OX=3702 GN=GLPK PE=1 SV=1
CmaG0027299.1	Glycerol kinase OS=Arabidopsis thaliana OX=3702 GN=GLPK PE=1 SV=1
CmaG0027304.1	Glycerol kinase OS=Arabidopsis thaliana OX=3702 GN=GLPK PE=1 SV=1

CmaG0027306.1	Glycerol kinase OS=Arabidopsis thaliana OX=3702 GN=GLPK PE=1 SV=1
CmaG0027311.1	Glycerol kinase OS=Arabidopsis thaliana OX=3702 GN=GLPK PE=1 SV=1
CmaG0027314.1	Glycerol kinase OS=Arabidopsis thaliana OX=3702 GN=GLPK PE=1 SV=1
Glycerol-3-phosphate acyltransferase	
CmaG0015847.1	Glycerol-3-phosphate acyltransferase 1 OS=Arabidopsis thaliana OX=3702 GN=GPAT1 PE=1 SV=1
CmaG0027901.1	Glycerol-3-phosphate acyltransferase 7 OS=Arabidopsis thaliana OX=3702 GN=GPAT7 PE=1 SV=1
CmaG0001614.1	Glycerol-3-phosphate acyltransferase 9 OS=Arabidopsis thaliana OX=3702 GN=GPAT9 PE=1 SV=1
CmaG0019494.1	Glycerol-3-phosphate acyltransferase RAM2 OS=Medicago truncatula OX=3880 GN=RAM2 PE=2 SV=1
CmaG0021077.1	Glycerol-3-phosphate acyltransferase, chloroplastic OS=Cucumis sativus OX=3659 PE=2 SV=1
CmaG0012678.1	Probable glycerol-3-phosphate acyltransferase 2 OS=Arabidopsis thaliana OX=3702 GN=GPAT2 PE=2 SV=1
CmaG0002649.1	Probable glycerol-3-phosphate acyltransferase 3 OS=Arabidopsis thaliana OX=3702 GN=GPAT3 PE=2 SV=1
CmaG0013758.1	Probable glycerol-3-phosphate acyltransferase 3 OS=Arabidopsis thaliana OX=3702 GN=GPAT3 PE=2 SV=1
CmaG0006886.1	Probable glycerol-3-phosphate acyltransferase 8 OS=Arabidopsis thaliana OX=3702 GN=GPAT8 PE=2 SV=1
1-acyl-sn-glycerol-3-phosphate acyltransferase	
CmaG0008100.1	1-acyl-sn-glycerol-3-phosphate acyltransferase 2 OS=Brassica napus OX=3708 GN=LPAT2 PE=2 SV=1
CmaG0011582.1	1-acyl-sn-glycerol-3-phosphate acyltransferase 2 OS=Brassica napus OX=3708 GN=LPAT2 PE=2 SV=1
CmaG0010050.1	1-acyl-sn-glycerol-3-phosphate acyltransferase BAT2, chloroplastic OS=Brassica napus OX=3708 GN=BAT2 PE=1 SV=2
CmaG0022832.1	1-acyl-sn-glycerol-3-phosphate acyltransferase OS=Cocos nucifera OX=13894 PE=1 SV=1
CmaG0017761.1	Probable 1-acyl-sn-glycerol-3-phosphate acyltransferase 4 OS=Arabidopsis thaliana OX=3702 GN=LPAT4 PE=2 SV=1
CmaG0001240.1	Probable 1-acyl-sn-glycerol-3-phosphate acyltransferase 5 OS=Arabidopsis thaliana OX=3702 GN=LPAT5 PE=2 SV=1
NAD（P+）-dependent Glycerol-3-Phosphate dehydrogenase	
CmaG0000529.1	Probable glycerol-3-phosphate dehydrogenase [NAD(+)] 1, cytosolic OS=Oryza sativa subsp. japonica OX=39947 GN=Os01g0939600 PE=2 SV=1
CmaG0008017.1	Probable glycerol-3-phosphate dehydrogenase [NAD(+)] 1, cytosolic OS=Oryza sativa subsp. japonica OX=39947 GN=Os01g0939600 PE=2 SV=1
CmaG0028202.1	Glycerol-3-phosphate dehydrogenase [NAD(+)] 2, chloroplastic OS=Arabidopsis thaliana OX=3702 GN=GLY1 PE=1 SV=1
CmaG0015509.1	Glycerol-3-phosphate dehydrogenase [NAD(+)] OS=Cuphea lanceolata OX=3930 GN=GPDH PE=2 SV=1
CmaG0010072.1	NADP-dependent glyceraldehyde-3-phosphate dehydrogenase OS=Nicotiana plumbaginifolia OX=4092 GN=GAPN PE=2 SV=1
CmaG0010828.1	NADP-dependent glyceraldehyde-3-phosphate dehydrogenase OS=Nicotiana plumbaginifolia OX=4092 GN=GAPN PE=2 SV=1
Hydroxyacyl-ACP dehydrase	
CmaG0016369.1	3-hydroxyacyl-[acyl-carrier-protein] dehydratase FabZ OS=Synechocystis sp. (strain PCC 6803 / Kazusa) OX=1111708 GN=fabZ PE=3 SV=2
CmaG0003849.1	3-hydroxyacyl-[acyl-carrier-protein] dehydratase FabZ OS=Thermosynechococcus elongatus (strain BP-1) OX=197221 GN=fabZ PE=3 SV=1
Hydroxyisobutyryl-CoA hydrolase	
CmaG0000699.1	3-hydroxyisobutyryl-CoA hydrolase 1 OS=Arabidopsis thaliana OX=3702 GN=CHY1 PE=1 SV=1
CmaG0000916.1	3-hydroxyisobutyryl-CoA hydrolase-like protein 1, mitochondrial OS=Arabidopsis thaliana OX=3702 GN=At3g60510 PE=1 SV=1
CmaG0010818.1	3-hydroxyisobutyryl-CoA hydrolase-like protein 2, mitochondrial OS=Arabidopsis thaliana OX=3702 GN=At4g31810 PE=1 SV=1

CmaG0023643.1	3-hydroxyisobutyryl-CoA hydrolase-like protein 3, mitochondrial OS=Arabidopsis thaliana OX=3702 GN=At4g13360 PE=1 SV=2
CmaG0015816.1	3-hydroxyisobutyryl-CoA hydrolase-like protein 5 OS=Arabidopsis thaliana OX=3702 GN=At1g06550 PE=1 SV=2
Ketoacyl-ACP reductase	
CmaG0008442.1	3-oxoacyl-[acyl-carrier-protein] reductase FabG OS=Bacillus subtilis (strain 168) OX=224308 GN=fabG PE=3 SV=3
CmaG0015621.1	3-oxoacyl-[acyl-carrier-protein] reductase FabG OS=Bacillus subtilis (strain 168) OX=224308 GN=fabG PE=3 SV=3
CmaG0008020.1	3-oxoacyl-[acyl-carrier-protein] reductase FabG OS=Thermotoga maritima (strain ATCC 43589 / MSB8 / DSM 3109 / JCM 10099) OX=243274 GN=fabG PE=3 SV=1
CmaG0019778.1	3-oxoacyl-[acyl-carrier-protein] reductase, chloroplastic OS=Cuphea lanceolata OX=3930 GN=CLKR27 PE=2 SV=1
CmaG0020184.1	3-oxoacyl-[acyl-carrier-protein] reductase, chloroplastic OS=Cuphea lanceolata OX=3930 GN=CLKR27 PE=2 SV=1
Ketoacyl-ACP synthase	
CmaG0020788.1	3-oxoacyl-[acyl-carrier-protein] synthase 3 A, chloroplastic OS=Cuphea wrightii OX=35942 GN=KAS3A PE=2 SV=2
CmaG0022259.1	3-oxoacyl-[acyl-carrier-protein] synthase I, chloroplastic OS=Arabidopsis thaliana OX=3702 GN=KAS1 PE=1 SV=2
CmaG0024421.1	3-oxoacyl-[acyl-carrier-protein] synthase I, chloroplastic OS=Arabidopsis thaliana OX=3702 GN=KAS1 PE=1 SV=2
CmaG0023221.1	3-oxoacyl-[acyl-carrier-protein] synthase II, chloroplastic OS=Arabidopsis thaliana OX=3702 GN=KAS2 PE=1 SV=1
CmaG0010469.1	3-oxoacyl-[acyl-carrier-protein] synthase, mitochondrial OS=Arabidopsis thaliana OX=3702 GN=KAS PE=1 SV=1
Ketoacyl-CoA thiolase	
CmaG0016176.1	3-ketoacyl-CoA thiolase 2, peroxisomal OS=Arabidopsis thaliana OX=3702 GN=PED1 PE=1 SV=2
CmaG0018930.1	3-ketoacyl-CoA thiolase 2, peroxisomal OS=Arabidopsis thaliana OX=3702 GN=PED1 PE=1 SV=2
Ketoacyl-CoA reductase	
CmaG0016368.1	Very-long-chain 3-oxoacyl-CoA reductase 1 OS=Arabidopsis thaliana OX=3702 GN=KCR1 PE=1 SV=1
CmaG0019730.1	Very-long-chain 3-oxoacyl-CoA reductase 1 OS=Arabidopsis thaliana OX=3702 GN=KCR1 PE=1 SV=1
CmaG0019731.1	Very-long-chain 3-oxoacyl-CoA reductase 1 OS=Arabidopsis thaliana OX=3702 GN=KCR1 PE=1 SV=1
CmaG0019732.1	Very-long-chain 3-oxoacyl-CoA reductase 1 OS=Arabidopsis thaliana OX=3702 GN=KCR1 PE=1 SV=1
CmaG0019734.1	Very-long-chain 3-oxoacyl-CoA reductase 1 OS=Arabidopsis thaliana OX=3702 GN=KCR1 PE=1 SV=1
CmaG0019735.1	Very-long-chain 3-oxoacyl-CoA reductase 1 OS=Arabidopsis thaliana OX=3702 GN=KCR1 PE=1 SV=1
CmaG0019736.1	Very-long-chain 3-oxoacyl-CoA reductase 1 OS=Arabidopsis thaliana OX=3702 GN=KCR1 PE=1 SV=1
CmaG0022784.1	Very-long-chain 3-oxoacyl-CoA reductase 1 OS=Arabidopsis thaliana OX=3702 GN=KCR1 PE=1 SV=1
Ketoacyl-CoA synthase	
CmaG0007095.1	3-ketoacyl-CoA synthase 1 OS=Arabidopsis thaliana OX=3702 GN=KCS1 PE=1 SV=1
CmaG0010647.1	3-ketoacyl-CoA synthase 10 OS=Arabidopsis thaliana OX=3702 GN=FDH PE=1 SV=2
CmaG0017011.1	3-ketoacyl-CoA synthase 10 OS=Arabidopsis thaliana OX=3702 GN=FDH PE=1 SV=2
CmaG0010987.1	3-ketoacyl-CoA synthase 11 OS=Arabidopsis thaliana OX=3702 GN=KCS11 PE=1 SV=1
CmaG0018537.1	3-ketoacyl-CoA synthase 11 OS=Arabidopsis thaliana OX=3702 GN=KCS11 PE=1 SV=1
CmaG0018538.1	3-ketoacyl-CoA synthase 11 OS=Arabidopsis thaliana OX=3702 GN=KCS11 PE=1 SV=1
CmaG0018540.1	3-ketoacyl-CoA synthase 11 OS=Arabidopsis thaliana OX=3702 GN=KCS11 PE=1 SV=1
CmaG0022856.1	3-ketoacyl-CoA synthase 11 OS=Arabidopsis thaliana OX=3702 GN=KCS11 PE=1 SV=1
CmaG0023685.1	3-ketoacyl-CoA synthase 11 OS=Arabidopsis thaliana OX=3702 GN=KCS11 PE=1 SV=1
CmaG0026116.1	3-ketoacyl-CoA synthase 12 OS=Arabidopsis thaliana OX=3702 GN=KCS12 PE=2 SV=1
CmaG0017021.1	3-ketoacyl-CoA synthase 13 OS=Arabidopsis thaliana OX=3702 GN=HIC PE=2 SV=1
CmaG0025904.1	3-ketoacyl-CoA synthase 18 OS=Arabidopsis thaliana OX=3702 GN=FAE1 PE=1 SV=1

CmaG0027752.1	3-ketoacyl-CoA synthase 19 OS=Arabidopsis thaliana OX=3702 GN=KCS19 PE=2 SV=1
CmaG0015890.1	3-ketoacyl-CoA synthase 2 OS=Arabidopsis thaliana OX=3702 GN=KCS2 PE=2 SV=2
CmaG0022855.1	3-ketoacyl-CoA synthase 2 OS=Arabidopsis thaliana OX=3702 GN=KCS2 PE=2 SV=2
CmaG0022857.1	3-ketoacyl-CoA synthase 2 OS=Arabidopsis thaliana OX=3702 GN=KCS2 PE=2 SV=2
CmaG0001265.1	3-ketoacyl-CoA synthase 4 OS=Arabidopsis thaliana OX=3702 GN=KCS4 PE=2 SV=1
CmaG0017405.1	3-ketoacyl-CoA synthase 4 OS=Arabidopsis thaliana OX=3702 GN=KCS4 PE=2 SV=1
CmaG0013549.1	3-ketoacyl-CoA synthase 5 OS=Arabidopsis thaliana OX=3702 GN=KCS5 PE=2 SV=1
CmaG0014335.1	3-ketoacyl-CoA synthase 5 OS=Arabidopsis thaliana OX=3702 GN=KCS5 PE=2 SV=1
CmaG0017938.1	3-ketoacyl-CoA synthase 6 OS=Arabidopsis thaliana OX=3702 GN=CUT1 PE=1 SV=1
CmaG0021695.1	3-ketoacyl-CoA synthase 7 OS=Arabidopsis thaliana OX=3702 GN=KCS7 PE=2 SV=1
CmaG0025898.1	3-ketoacyl-CoA synthase 7 OS=Arabidopsis thaliana OX=3702 GN=KCS7 PE=2 SV=1
CmaG0025900.1	3-ketoacyl-CoA synthase 7 OS=Arabidopsis thaliana OX=3702 GN=KCS7 PE=2 SV=1
CmaG0025902.1	3-ketoacyl-CoA synthase 7 OS=Arabidopsis thaliana OX=3702 GN=KCS7 PE=2 SV=1
CmaG0016176.1	3-ketoacyl-CoA thiolase 2, peroxisomal OS=Arabidopsis thaliana OX=3702 GN=PED1 PE=1 SV=2
CmaG0018930.1	3-ketoacyl-CoA thiolase 2, peroxisomal OS=Arabidopsis thaliana OX=3702 GN=PED1 PE=1 SV=2
3-deoxy-D-manno-octulosonic acid (Kdo) transferase	
CmaG0007711.1	Probable 3-deoxy-D-manno-octulosonic acid transferase, mitochondrial OS=Arabidopsis thaliana OX=3702 GN=KDTA PE=2 SV=1
Acyl-CoA synthetase	
CmaG0013762.1	Long chain acyl-CoA synthetase 1 OS=Arabidopsis thaliana OX=3702 GN=LACS1 PE=2 SV=1
CmaG0001214.1	Long chain acyl-CoA synthetase 2 OS=Arabidopsis thaliana OX=3702 GN=LACS2 PE=2 SV=1
CmaG0001216.1	Long chain acyl-CoA synthetase 2 OS=Arabidopsis thaliana OX=3702 GN=LACS2 PE=2 SV=1
CmaG0001219.1	Long chain acyl-CoA synthetase 2 OS=Arabidopsis thaliana OX=3702 GN=LACS2 PE=2 SV=1
CmaG0021733.1	Long chain acyl-CoA synthetase 4 OS=Arabidopsis thaliana OX=3702 GN=LACS4 PE=2 SV=1
CmaG0003039.1	Long chain acyl-CoA synthetase 7, peroxisomal OS=Arabidopsis thaliana OX=3702 GN=LACS7 PE=1 SV=2
CmaG0012831.1	Long chain acyl-CoA synthetase 7, peroxisomal OS=Arabidopsis thaliana OX=3702 GN=LACS7 PE=1 SV=2
CmaG0010594.1	Long chain acyl-CoA synthetase 8 OS=Arabidopsis thaliana OX=3702 GN=LACS8 PE=1 SV=1
CmaG0002535.1	Long chain acyl-CoA synthetase 9, chloroplastic OS=Arabidopsis thaliana OX=3702 GN=LACS9 PE=1 SV=1
Lipoxygenase	
CmaG0003031.1	Probable linoleate 9S-lipoxygenase 5 OS=Solanum tuberosum OX=4113 GN=LOX1.5 PE=2 SV=1
CmaG0012825.1	Probable linoleate 9S-lipoxygenase 5 OS=Solanum tuberosum OX=4113 GN=LOX1.5 PE=2 SV=1
CmaG0012826.1	Probable linoleate 9S-lipoxygenase 5 OS=Solanum tuberosum OX=4113 GN=LOX1.5 PE=2 SV=1
CmaG0012827.1	Probable linoleate 9S-lipoxygenase 5 OS=Solanum tuberosum OX=4113 GN=LOX1.5 PE=2 SV=1
CmaG0011654.1	Linoleate 13S-lipoxygenase 2-1, chloroplastic OS=Solanum tuberosum OX=4113 GN=LOX2.1 PE=1 SV=1
CmaG0011655.1	Linoleate 13S-lipoxygenase 2-1, chloroplastic OS=Solanum tuberosum OX=4113 GN=LOX2.1 PE=1 SV=1
CmaG0011656.1	Linoleate 13S-lipoxygenase 2-1, chloroplastic OS=Solanum tuberosum OX=4113 GN=LOX2.1 PE=1 SV=1
CmaG0011658.1	Linoleate 13S-lipoxygenase 2-1, chloroplastic OS=Solanum tuberosum OX=4113 GN=LOX2.1 PE=1 SV=1
CmaG0011659.1	Linoleate 13S-lipoxygenase 2-1, chloroplastic OS=Solanum tuberosum OX=4113 GN=LOX2.1 PE=1 SV=1
CmaG0026625.1	Linoleate 13S-lipoxygenase 2-1, chloroplastic OS=Solanum tuberosum OX=4113 GN=LOX2.1 PE=1 SV=1
CmaG0005200.1	Linoleate 13S-lipoxygenase 3-1, chloroplastic OS=Solanum tuberosum OX=4113 GN=LOX3.1 PE=1 SV=1
CmaG0007443.1	Linoleate 13S-lipoxygenase 3-1, chloroplastic OS=Solanum tuberosum OX=4113 GN=LOX3.1 PE=1 SV=1
CmaG0000028.1	Linoleate 9S-lipoxygenase A OS=Solanum lycopersicum OX=4081 GN=LOX1.1 PE=2 SV=1
CmaG0019788.1	Lipoxygenase 6, chloroplastic OS=Arabidopsis thaliana OX=3702 GN=LOX6 PE=2 SV=1
Dihydrolipoamide dehydrogenase	

Lysophospholipid acyltransferase	
CmaG0022087.1	Lysophospholipid acyltransferase 1 OS=Arabidopsis thaliana OX=3702 GN=LPLAT1 PE=1 SV=1
CmaG0017749.1	Lysophospholipid acyltransferase LPEAT1 OS=Arabidopsis thaliana OX=3702 GN=LPEAT1 PE=1 SV=1
CmaG0027437.1	Lysophospholipid acyltransferase LPEAT2 OS=Arabidopsis thaliana OX=3702 GN=LPEAT2 PE=1 SV=1
UDP-N-acetylglucosamine acyltransferase	
CmaG0023399.1	Probable acyl-[acyl-carrier-protein]--UDP-N-acetylglucosamine O-acyltransferase, mitochondrial OS=Arabidopsis thaliana OX=3702 GN=LPXA PE=1 SV=1
Lipid-A-disaccharide synthase	
CmaG0010464.1	Probable lipid-A-disaccharide synthase, mitochondrial OS=Arabidopsis thaliana OX=3702 GN=LPXB PE=2 SV=1
UDP-3-O-acyl N-acetylglucosamine deacetylase	
CmaG0018147.1	Probable UDP-3-O-acyl-N-acetylglucosamine deacetylase 5, mitochondrial OS=Arabidopsis thaliana OX=3702 GN=LPXC5 PE=1 SV=2
UDP-3-O-(3-hydroxymyristoyl)glucosamine N-acyltransferase	
CmaG0005540.1	Probable UDP-3-O-acylglucosamine N-acyltransferase 2, mitochondrial OS=Arabidopsis thaliana OX=3702 GN=LPXD2 PE=3 SV=1
Tetraacyldisaccharide 4'-kinase	
CmaG0009020.1	Probable tetraacyldisaccharide 4'-kinase, mitochondrial OS=Arabidopsis thaliana OX=3702 GN=LPXK PE=2 SV=1
CmaG0022863.1	Probable tetraacyldisaccharide 4'-kinase, mitochondrial OS=Arabidopsis thaliana OX=3702 GN=LPXK PE=2 SV=1
Lipid transfer protein type	
CmaG0000094.1	Putative lipid-transfer protein DIR1 OS=Arabidopsis thaliana OX=3702 GN=DIR1 PE=1 SV=1
CmaG0001242.1	Putative lipid-transfer protein DIR1 OS=Arabidopsis thaliana OX=3702 GN=DIR1 PE=1 SV=1
CmaG0018361.1	Putative lipid-transfer protein DIR1 OS=Arabidopsis thaliana OX=3702 GN=DIR1 PE=1 SV=1
CmaG0027987.1	Putative lipid-transfer protein DIR1 OS=Arabidopsis thaliana OX=3702 GN=DIR1 PE=1 SV=1
CmaG0014264.1	Putative non-specific lipid-transfer protein 14 OS=Arabidopsis thaliana OX=3702 GN=LTP14 PE=3 SV=2
CmaG0026657.1	Non-specific lipid-transfer protein 1 OS=Prunus dulcis OX=3755 PE=3 SV=1
CmaG0026658.1	Non-specific lipid-transfer protein 1 OS=Prunus dulcis OX=3755 PE=3 SV=1
CmaG0014787.1	Non-specific lipid-transfer protein 2 OS=Apium graveolens var. rapaceum OX=278110 PE=1 SV=1
CmaG0000516.1	Non-specific lipid-transfer protein 3 OS=Prunus dulcis OX=3755 PE=2 SV=1
CmaG0008731.1	Non-specific lipid-transfer protein 3 OS=Prunus dulcis OX=3755 PE=2 SV=1
CmaG0013566.1	Non-specific lipid-transfer protein 3 OS=Prunus dulcis OX=3755 PE=2 SV=1
CmaG0000313.1	Non-specific lipid-transfer protein 8 OS=Arabidopsis thaliana OX=3702 GN=LTP8 PE=3 SV=1
CmaG0005737.1	Non-specific lipid-transfer protein AP10 OS=Helianthus annuus OX=4232 PE=1 SV=2
CmaG0022343.1	Non-specific lipid-transfer protein AP10 OS=Helianthus annuus OX=4232 PE=1 SV=2
CmaG0008668.1	Non-specific lipid-transfer protein Cor a 8 OS=Corylus avellana OX=13451 PE=1 SV=1
CmaG0023999.1	Non-specific lipid-transfer protein Cw18 OS=Hordeum vulgare OX=4513 GN=CW18 PE=1 SV=1
CmaG0008665.1	Non-specific lipid-transfer protein OS=Gossypium hirsutum OX=3635 PE=2 SV=1
CmaG0008666.1	Non-specific lipid-transfer protein OS=Gossypium hirsutum OX=3635 PE=2 SV=1
CmaG0008667.1	Non-specific lipid-transfer protein OS=Gossypium hirsutum OX=3635 PE=2 SV=1
CmaG0026656.1	Non-specific lipid-transfer protein OS=Helianthus annuus OX=4232 PE=3 SV=1
CmaG0026659.1	Non-specific lipid-transfer protein OS=Helianthus annuus OX=4232 PE=3 SV=1

CmaG0005731.1	Non-specific lipid-transfer protein OS=Pinus taeda OX=3352 PE=2 SV=1
CmaG0005732.1	Non-specific lipid-transfer protein OS=Pinus taeda OX=3352 PE=2 SV=1
CmaG0005733.1	Non-specific lipid-transfer protein OS=Pinus taeda OX=3352 PE=2 SV=1
CmaG0005734.1	Non-specific lipid-transfer protein OS=Pinus taeda OX=3352 PE=2 SV=1
CmaG0005735.1	Non-specific lipid-transfer protein OS=Pinus taeda OX=3352 PE=2 SV=1
CmaG0005736.1	Non-specific lipid-transfer protein OS=Pinus taeda OX=3352 PE=2 SV=1
CmaG0002980.1	Non-specific lipid-transfer protein-like protein At2g13820 OS=Arabidopsis thaliana OX=3702 GN=At2g13820 PE=2 SV=1
CmaG0013326.1	Non-specific lipid-transfer protein-like protein At2g13820 OS=Arabidopsis thaliana OX=3702 GN=At2g13820 PE=2 SV=1
CmaG0002669.1	Non-specific lipid-transfer protein-like protein At5g64080 OS=Arabidopsis thaliana OX=3702 GN=At5g64080 PE=2 SV=1
CmaG0003854.1	Non-specific lipid-transfer protein-like protein At5g64080 OS=Arabidopsis thaliana OX=3702 GN=At5g64080 PE=2 SV=1
Monoacylglycerol lipase (MAGL)	
CmaG0016298.1	Monoacylglycerol lipase ABHD6 OS=Mus musculus OX=10090 GN=Abhd6 PE=1 SV=1
CmaG0025302.1	Monoacylglycerol lipase ABHD6 OS=Mus musculus OX=10090 GN=Abhd6 PE=1 SV=1
Malonyl-CoA decarboxylase	
CmaG0005683.1	Malonyl-CoA decarboxylase, mitochondrial OS=Homo sapiens OX=9606 GN=MLYCD PE=1 SV=3
Malonyl-CoA:ACP malonyltransferase	
CmaG0026592.1	Malonyl-CoA-acyl carrier protein transacylase, mitochondrial OS=Homo sapiens OX=9606 GN=MCAT PE=1 SV=2
Enoyl-CoA hydratase	
CmaG0012692.1	Golgi apparatus membrane protein-like protein ECHIDNA OS=Arabidopsis thaliana OX=3702 GN=ECH PE=1 SV=1
CmaG0013772.1	Golgi apparatus membrane protein-like protein ECHIDNA OS=Arabidopsis thaliana OX=3702 GN=ECH PE=1 SV=1
CmaG0005499.1	Probable enoyl-CoA hydratase 1, peroxisomal OS=Arabidopsis thaliana OX=3702 GN=ECHIA PE=1 SV=1
CmaG0021471.1	Probable enoyl-CoA hydratase 1, peroxisomal OS=Arabidopsis thaliana OX=3702 GN=ECHIA PE=1 SV=1
CmaG0020887.1	Probable enoyl-CoA hydratase 2, mitochondrial OS=Arabidopsis thaliana OX=3702 GN=At4g16800 PE=2 SV=1
CmaG0024826.1	Enoyl-CoA hydratase 2, peroxisomal OS=Arabidopsis thaliana OX=3702 GN=ECH2 PE=1 SV=1
CmaG0024827.1	Enoyl-CoA hydratase 2, peroxisomal OS=Arabidopsis thaliana OX=3702 GN=ECH2 PE=1 SV=1
CmaG0026011.1	(R)-specific enoyl-CoA hydratase OS=Aeromonas caviae OX=648 GN=phaJ PE=1 SV=1
hydroxyacyl-CoA dehydratase	
CmaG0026136.1	Very-long-chain (3R)-3-hydroxyacyl-CoA dehydratase 2 OS=Bos taurus OX=9913 GN=HACD2 PE=2 SV=2
CmaG0012481.1	Very-long-chain (3R)-3-hydroxyacyl-CoA dehydratase PASTICCINO 2A OS=Oryza sativa subsp. japonica OX=39947 GN=PAS2A PE=2 SV=2
Monogalactosyldiacylglycerol synthase	
CmaG0023851.1	Monogalactosyldiacylglycerol synthase 2, chloroplastic OS=Arabidopsis thaliana OX=3702 GN=MGD2 PE=1 SV=1
CmaG0010793.1	Probable monogalactosyldiacylglycerol synthase, chloroplastic OS=Nicotiana tabacum OX=4097 GN=MGDA PE=2 SV=1
myo-inositol-3-phosphate synthase	
CmaG0016371.1	Inositol-3-phosphate synthase OS=Nicotiana tabacum OX=4097 PE=2 SV=1
Phospholipase C (non specific)	

CmaG0026464.1	Non-specific phospholipase C1 OS=Arabidopsis thaliana OX=3702 GN=NPC1 PE=2 SV=1
CmaG0011459.1	Non-specific phospholipase C2 OS=Arabidopsis thaliana OX=3702 GN=NPC2 PE=2 SV=1
CmaG0007201.1	Non-specific phospholipase C4 OS=Arabidopsis thaliana OX=3702 GN=NPC4 PE=1 SV=1
CmaG0020096.1	Non-specific phospholipase C6 OS=Arabidopsis thaliana OX=3702 GN=NPC6 PE=2 SV=1
Oil-Body oleosin	
CmaG0025366.1	Oleosin 1 OS=Prunus dulcis OX=3755 GN=OLE1 PE=2 SV=1
CmaG0009613.1	Oleosin 16 kDa OS=Bromus secalinus OX=4502 GN=OLE16 PE=2 SV=1
CmaG0025644.1	Oleosin 16 kDa OS=Oryza sativa subsp. japonica OX=39947 GN=OLE16 PE=2 SV=3
CmaG0023104.1	Oleosin 18.5 kDa OS=Arabidopsis thaliana OX=3702 GN=At4g25140 PE=2 SV=1
CmaG0015454.1	Oleosin 5 OS=Arabidopsis thaliana OX=3702 GN=At3g01570 PE=2 SV=1
CmaG0020499.1	Oleosin 5 OS=Arabidopsis thaliana OX=3702 GN=At3g01570 PE=2 SV=1
CmaG0023126.1	Oleosin 5 OS=Arabidopsis thaliana OX=3702 GN=At3g01570 PE=2 SV=1
NAD+ oxidoreductase (involved in fatty acid alpha-oxidation)	
CmaG0023738.1	NADPH-dependent oxidoreductase 2-alkenal reductase OS=Arabidopsis thaliana OX=3702 GN=AER PE=1 SV=1
CmaG0007992.1	NAD(P)H-quinone oxidoreductase subunit H, chloroplastic OS=Ranunculus macranthus OX=334596 GN=ndhH PE=3 SV=1
CmaG0002973.1	NAD(P)H-quinone oxidoreductase subunit I, chloroplastic OS=Nandina domestica OX=41776 GN=ndhI PE=3 SV=1
CmaG0018223.1	NAD(P)H-quinone oxidoreductase subunit L, chloroplastic OS=Arabidopsis thaliana OX=3702 GN=ndhL PE=2 SV=1
CmaG0017737.1	NAD(P)H-quinone oxidoreductase subunit M, chloroplastic OS=Vitis vinifera OX=29760 GN=ndhM PE=2 SV=1
CmaG0026651.1	NAD(P)H-quinone oxidoreductase subunit N, chloroplastic OS=Arabidopsis thaliana OX=3702 GN=ndhN PE=2 SV=1
CmaG0026696.1	NAD(P)H-quinone oxidoreductase subunit N, chloroplastic OS=Arabidopsis thaliana OX=3702 GN=ndhN PE=2 SV=1
CmaG0016647.1	NAD(P)H-quinone oxidoreductase subunit O, chloroplastic OS=Arabidopsis thaliana OX=3702 GN=ndhO PE=2 SV=1
CmaG0022226.1	NAD(P)H-quinone oxidoreductase subunit S, chloroplastic OS=Arabidopsis thaliana OX=3702 GN=ndhS PE=1 SV=1
CmaG0014452.1	NAD(P)H-quinone oxidoreductase subunit T, chloroplastic OS=Arabidopsis thaliana OX=3702 GN=ndhT PE=1 SV=1
CmaG0017216.1	NAD(P)H-quinone oxidoreductase subunit U, chloroplastic OS=Arabidopsis thaliana OX=3702 GN=ndhU PE=1 SV=1
CmaG0000623.1	NADPH:quinone oxidoreductase OS=Arabidopsis thaliana OX=3702 GN=NQR PE=1 SV=1
CmaG0024018.1	Alternative NAD(P)H-ubiquinone oxidoreductase C1, chloroplastic/mitochondrial OS=Arabidopsis thaliana OX=3702 GN=NDC1 PE=1 SV=2
CmaG0006268.1	External alternative NAD(P)H-ubiquinone oxidoreductase B1, mitochondrial OS=Solanum tuberosum OX=4113 GN=NDB1 PE=3 SV=1
CmaG0026792.1	External alternative NAD(P)H-ubiquinone oxidoreductase B1, mitochondrial OS=Solanum tuberosum OX=4113 GN=NDB1 PE=3 SV=1
CmaG0005370.1	External alternative NAD(P)H-ubiquinone oxidoreductase B2, mitochondrial OS=Arabidopsis thaliana OX=3702 GN=NDB2 PE=1 SV=1
CmaG0005372.1	External alternative NAD(P)H-ubiquinone oxidoreductase B2, mitochondrial OS=Arabidopsis thaliana OX=3702 GN=NDB2 PE=1 SV=1
CmaG0006269.1	External alternative NAD(P)H-ubiquinone oxidoreductase B3, mitochondrial OS=Arabidopsis thaliana OX=3702 GN=NDB3 PE=2 SV=1
CmaG0009367.1	Internal alternative NAD(P)H-ubiquinone oxidoreductase A1, mitochondrial OS=Arabidopsis thaliana OX=3702 GN=NDA1 PE=2 SV=1
CmaG0009369.1	Internal alternative NAD(P)H-ubiquinone oxidoreductase A1, mitochondrial OS=Arabidopsis thaliana OX=3702 GN=NDA1 PE=2 SV=1
CmaG0026503.1	Internal alternative NAD(P)H-ubiquinone oxidoreductase A1, mitochondrial OS=Arabidopsis thaliana OX=3702 GN=NDA1 PE=2 SV=1

CmaG0026504.1	Internal alternative NAD(P)H-ubiquinone oxidoreductase A1, mitochondrial OS=Arabidopsis thaliana OX=3702 GN=NDA1 PE=2 SV=1
CmaG0026505.1	Internal alternative NAD(P)H-ubiquinone oxidoreductase A1, mitochondrial OS=Arabidopsis thaliana OX=3702 GN=NDA1 PE=2 SV=1
CmaG0026507.1	Internal alternative NAD(P)H-ubiquinone oxidoreductase A1, mitochondrial OS=Arabidopsis thaliana OX=3702 GN=NDA1 PE=2 SV=1
CmaG0026510.1	Internal alternative NAD(P)H-ubiquinone oxidoreductase A1, mitochondrial OS=Arabidopsis thaliana OX=3702 GN=NDA1 PE=2 SV=1
CmaG0026511.1	Internal alternative NAD(P)H-ubiquinone oxidoreductase A1, mitochondrial OS=Arabidopsis thaliana OX=3702 GN=NDA1 PE=2 SV=1
CmaG0026513.1	Internal alternative NAD(P)H-ubiquinone oxidoreductase A1, mitochondrial OS=Arabidopsis thaliana OX=3702 GN=NDA1 PE=2 SV=1
CmaG0026515.1	Internal alternative NAD(P)H-ubiquinone oxidoreductase A1, mitochondrial OS=Arabidopsis thaliana OX=3702 GN=NDA1 PE=2 SV=1
CmaG0025853.1	Internal alternative NAD(P)H-ubiquinone oxidoreductase A1, mitochondrial OS=Solanum tuberosum OX=4113 GN=NDA1 PE=3 SV=1
CmaG0005342.1	Internal alternative NAD(P)H-ubiquinone oxidoreductase A2, mitochondrial OS=Arabidopsis thaliana OX=3702 GN=NDA2 PE=1 SV=1
CmaG0005344.1	Internal alternative NAD(P)H-ubiquinone oxidoreductase A2, mitochondrial OS=Arabidopsis thaliana OX=3702 GN=NDA2 PE=1 SV=1
CmaG0026496.1	Internal alternative NAD(P)H-ubiquinone oxidoreductase A2, mitochondrial OS=Arabidopsis thaliana OX=3702 GN=NDA2 PE=1 SV=1
CmaG0026499.1	Internal alternative NAD(P)H-ubiquinone oxidoreductase A2, mitochondrial OS=Arabidopsis thaliana OX=3702 GN=NDA2 PE=1 SV=1
Phospholipid:diacylglycerol acyltransferase	
CmaG0007407.1	Phospholipid:diacylglycerol acyltransferase 1 OS=Arabidopsis thaliana OX=3702 GN=PDAT1 PE=2 SV=1
CmaG0023312.1	Phospholipid:diacylglycerol acyltransferase 1 OS=Arabidopsis thaliana OX=3702 GN=PDAT1 PE=2 SV=1
CmaG0023313.1	Phospholipid:diacylglycerol acyltransferase 1 OS=Arabidopsis thaliana OX=3702 GN=PDAT1 PE=2 SV=1
CmaG0012141.1	Putative phospholipid:diacylglycerol acyltransferase 2 OS=Arabidopsis thaliana OX=3702 GN=PDAT2 PE=3 SV=1
Phosphatidylcholine:diacylglycerol cholinephosphotransferase	
CmaG0027262.1	Phosphatidylcholine:diacylglycerol cholinephosphotransferase 1 OS=Arabidopsis thaliana OX=3702 GN=ROD1 PE=1 SV=1
Pyruvate dehydrogenase	
CmaG0019526.1	Pyruvate dehydrogenase E1 component subunit alpha-1, mitochondrial OS=Arabidopsis thaliana OX=3702 GN=E1 ALPHA PE=1 SV=2
CmaG0007111.1	Pyruvate dehydrogenase E1 component subunit alpha-3, chloroplastic OS=Arabidopsis thaliana OX=3702 GN=PDH-E1 ALPHA PE=2 SV=1
CmaG0019902.1	Pyruvate dehydrogenase E1 component subunit beta-1, mitochondrial OS=Oryza sativa subsp. japonica OX=39947 GN=Os08g0536000 PE=2 SV=1
CmaG0024376.1	Pyruvate dehydrogenase E1 component subunit beta-3, chloroplastic OS=Arabidopsis thaliana OX=3702 GN=E1-BETA-2 PE=2 SV=1
CmaG0001020.1	Dihydrolipoyllysine-residue acetyltransferase component 1 of pyruvate dehydrogenase complex, mitochondrial OS=Arabidopsis thaliana OX=3702 GN=LTA3 PE=1 SV=2
CmaG0007384.1	Dihydrolipoyllysine-residue acetyltransferase component 2 of pyruvate dehydrogenase complex, mitochondrial OS=Arabidopsis thaliana OX=3702 GN=At3g13930 PE=1 SV=2
CmaG0007385.1	Dihydrolipoyllysine-residue acetyltransferase component 3 of pyruvate dehydrogenase complex, mitochondrial OS=Arabidopsis thaliana OX=3702 GN=At1g54220 PE=1 SV=1
CmaG0017948.1	Dihydrolipoyllysine-residue acetyltransferase component 4 of pyruvate dehydrogenase complex, chloroplastic OS=Arabidopsis thaliana OX=3702 GN=LTA2 PE=2 SV=1
CmaG0017441.1	Dihydrolipoyllysine-residue acetyltransferase component 5 of pyruvate dehydrogenase complex, chloroplastic OS=Arabidopsis thaliana OX=3702 GN=EMB3003 PE=2 SV=1
CmaG0002897.1	[Pyruvate dehydrogenase (acetyl-transferring)] kinase, mitochondrial OS=Arabidopsis thaliana OX=3702 GN=PDK PE=1 SV=1
Phosphoethanolamine N-Methyltransferase	

CmaG0009728.1	Phosphoethanolamine N-methyltransferase 1 OS=Arabidopsis thaliana OX=3702 GN=NMT1 PE=1 SV=1
CmaG0009729.1	Phosphoethanolamine N-methyltransferase 1 OS=Arabidopsis thaliana OX=3702 GN=NMT1 PE=1 SV=1
Phosphatidylinositol-3-Kinase	
CmaG0001308.1	Phosphatidylinositol 4-kinase alpha 1 OS=Arabidopsis thaliana OX=3702 GN=PI4KA1 PE=1 SV=2
CmaG0016638.1	Phosphatidylinositol 4-kinase beta 1 OS=Arabidopsis thaliana OX=3702 GN=PI4KB1 PE=1 SV=1
CmaG0008491.1	Phosphatidylinositol 4-kinase gamma 1 OS=Arabidopsis thaliana OX=3702 GN=PI4KG1 PE=2 SV=1
CmaG0002245.1	Phosphatidylinositol 4-kinase gamma 3 OS=Arabidopsis thaliana OX=3702 GN=PI4KG3 PE=2 SV=1
CmaG0007036.1	Phosphatidylinositol 4-kinase gamma 4 OS=Arabidopsis thaliana OX=3702 GN=PI4KG4 PE=1 SV=1
CmaG0022243.1	Phosphatidylinositol 4-kinase gamma 4 OS=Arabidopsis thaliana OX=3702 GN=PI4KG4 PE=1 SV=1
CmaG0015016.1	Phosphatidylinositol 4-kinase gamma 7 OS=Arabidopsis thaliana OX=3702 GN=PI4KG7 PE=1 SV=2
CmaG0019240.1	Phosphatidylinositol 4-kinase gamma 7 OS=Arabidopsis thaliana OX=3702 GN=PI4KG7 PE=1 SV=2
Phospholipase A	
CmaG0005584.1	Phospholipase A I OS=Arabidopsis thaliana OX=3702 GN=PLA1 PE=3 SV=1
CmaG0000817.1	Phospholipase A(1) DAD1, chloroplastic OS=Arabidopsis thaliana OX=3702 GN=DAD1 PE=1 SV=1
CmaG0000818.1	Phospholipase A(1) DAD1, chloroplastic OS=Arabidopsis thaliana OX=3702 GN=DAD1 PE=1 SV=1
CmaG0007010.1	Phospholipase A1 PLIP1, chloroplastic OS=Arabidopsis thaliana OX=3702 GN=PLIP1 PE=1 SV=1
CmaG0013700.1	Phospholipase A1 PLIP2, chloroplastic OS=Arabidopsis thaliana OX=3702 GN=PLIP2 PE=1 SV=1
CmaG0020895.1	Phospholipase A1-Ibeta2, chloroplastic OS=Arabidopsis thaliana OX=3702 GN=At4g16820 PE=1 SV=2
CmaG0024287.1	Phospholipase A1-Igamma1, chloroplastic OS=Arabidopsis thaliana OX=3702 GN=At1g06800 PE=1 SV=2
CmaG0024293.1	Phospholipase A1-Igamma1, chloroplastic OS=Arabidopsis thaliana OX=3702 GN=At1g06800 PE=1 SV=2
CmaG0024295.1	Phospholipase A1-Igamma1, chloroplastic OS=Arabidopsis thaliana OX=3702 GN=At1g06800 PE=1 SV=2
CmaG0015904.1	Phospholipase A1-Igamma2, chloroplastic OS=Arabidopsis thaliana OX=3702 GN=At2g30550 PE=1 SV=2
CmaG0024296.1	Phospholipase A1-Igamma2, chloroplastic OS=Arabidopsis thaliana OX=3702 GN=At2g30550 PE=1 SV=2
CmaG0011540.1	Phospholipase A1-Igamma3, chloroplastic OS=Arabidopsis thaliana OX=3702 GN=At1g51440 PE=1 SV=1
CmaG0005000.1	Phospholipase A1-II 4 OS=Oryza sativa subsp. japonica OX=39947 GN=Os01g0652300 PE=2 SV=2
CmaG0005015.1	Phospholipase A1-II 4 OS=Oryza sativa subsp. japonica OX=39947 GN=Os01g0652300 PE=2 SV=2
CmaG0005006.1	Phospholipase A1-IIalpha OS=Arabidopsis thaliana OX=3702 GN=At1g06250 PE=2 SV=1
CmaG0005014.1	Phospholipase A1-IIalpha OS=Arabidopsis thaliana OX=3702 GN=At1g06250 PE=2 SV=1
CmaG0017312.1	Phospholipase A1-IIalpha OS=Arabidopsis thaliana OX=3702 GN=At1g06250 PE=2 SV=1
CmaG0005578.1	Phospholipase A1-IIbeta OS=Arabidopsis thaliana OX=3702 GN=At2g31100 PE=1 SV=2
CmaG0005967.1	Phospholipase A1-IIdelta OS=Arabidopsis thaliana OX=3702 GN=At2g42690 PE=1 SV=1
CmaG0005968.1	Phospholipase A1-IIdelta OS=Arabidopsis thaliana OX=3702 GN=At2g42690 PE=1 SV=1
CmaG0005969.1	Phospholipase A1-IIdelta OS=Arabidopsis thaliana OX=3702 GN=At2g42690 PE=1 SV=1
CmaG0005970.1	Phospholipase A1-IIdelta OS=Arabidopsis thaliana OX=3702 GN=At2g42690 PE=1 SV=1
CmaG0005971.1	Phospholipase A1-IIdelta OS=Arabidopsis thaliana OX=3702 GN=At2g42690 PE=1 SV=1
CmaG0012951.1	Phospholipase A1-IIdelta OS=Arabidopsis thaliana OX=3702 GN=At2g42690 PE=1 SV=1
CmaG0005001.1	Phospholipase A1-IIgamma OS=Arabidopsis thaliana OX=3702 GN=DSEL PE=1 SV=1
CmaG0005002.1	Phospholipase A1-IIgamma OS=Arabidopsis thaliana OX=3702 GN=DSEL PE=1 SV=1
CmaG0005003.1	Phospholipase A1-IIgamma OS=Arabidopsis thaliana OX=3702 GN=DSEL PE=1 SV=1
CmaG0005005.1	Phospholipase A1-IIgamma OS=Arabidopsis thaliana OX=3702 GN=DSEL PE=1 SV=1
CmaG0005007.1	Phospholipase A1-IIgamma OS=Arabidopsis thaliana OX=3702 GN=DSEL PE=1 SV=1
CmaG0005009.1	Phospholipase A1-IIgamma OS=Arabidopsis thaliana OX=3702 GN=DSEL PE=1 SV=1
CmaG0005016.1	Phospholipase A1-IIgamma OS=Arabidopsis thaliana OX=3702 GN=DSEL PE=1 SV=1
CmaG0005017.1	Phospholipase A1-IIgamma OS=Arabidopsis thaliana OX=3702 GN=DSEL PE=1 SV=1
CmaG0005018.1	Phospholipase A1-IIgamma OS=Arabidopsis thaliana OX=3702 GN=DSEL PE=1 SV=1
CmaG0005577.1	Phospholipase A1-IIgamma OS=Arabidopsis thaliana OX=3702 GN=DSEL PE=1 SV=1

CmaG0001239.1	Phospholipase A-2-activating protein OS=Homo sapiens OX=9606 GN=PLAA PE=1 SV=2
CmaG0002299.1	Phospholipase A2-alpha OS=Arabidopsis thaliana OX=3702 GN=PLA2-ALPHA PE=1 SV=1
CmaG0002300.1	Phospholipase A2-alpha OS=Arabidopsis thaliana OX=3702 GN=PLA2-ALPHA PE=1 SV=1
CmaG0023413.1	Probable phospholipase A2 homolog 1 OS=Oryza sativa subsp. japonica OX=39947 GN=PLA2-I PE=2 SV=1
PLAT domain-containing protein	
CmaG0016356.1	PLAT domain-containing protein 2 OS=Arabidopsis thaliana OX=3702 GN=PLAT2 PE=2 SV=1
Phospholipase D	
CmaG0010817.1	Probable phospholipase D F09G2.8 OS=Caenorhabditis elegans OX=6239 GN=F09G2.8 PE=1 SV=2
CmaG0010928.1	Phospholipase D alpha 1 OS=Carica papaya OX=3649 GN=PLD1 PE=1 SV=1
CmaG0023756.1	Phospholipase D alpha 1 OS=Ricinus communis OX=3988 GN=PLD1 PE=1 SV=1
CmaG0027241.1	Phospholipase D alpha 1 OS=Ricinus communis OX=3988 GN=PLD1 PE=1 SV=1
CmaG0007335.1	Phospholipase D alpha 4 OS=Arabidopsis thaliana OX=3702 GN=PLDALPHA4 PE=2 SV=1
CmaG0016661.1	Phospholipase D beta 1 OS=Arabidopsis thaliana OX=3702 GN=PLDBETA1 PE=1 SV=4
CmaG0022118.1	Phospholipase D beta 1 OS=Arabidopsis thaliana OX=3702 GN=PLDBETA1 PE=1 SV=4
CmaG0022120.1	Phospholipase D beta 1 OS=Arabidopsis thaliana OX=3702 GN=PLDBETA1 PE=1 SV=4
CmaG0022121.1	Phospholipase D beta 1 OS=Arabidopsis thaliana OX=3702 GN=PLDBETA1 PE=1 SV=4
CmaG0022123.1	Phospholipase D beta 1 OS=Arabidopsis thaliana OX=3702 GN=PLDBETA1 PE=1 SV=4
CmaG0022126.1	Phospholipase D beta 1 OS=Arabidopsis thaliana OX=3702 GN=PLDBETA1 PE=1 SV=4
CmaG0027432.1	Phospholipase D beta 1 OS=Arabidopsis thaliana OX=3702 GN=PLDBETA1 PE=1 SV=4
CmaG0012307.1	Phospholipase D delta OS=Arabidopsis thaliana OX=3702 GN=PLDDELTA PE=1 SV=2
CmaG0012308.1	Phospholipase D delta OS=Arabidopsis thaliana OX=3702 GN=PLDDELTA PE=1 SV=2
CmaG0024855.1	Phospholipase D delta OS=Arabidopsis thaliana OX=3702 GN=PLDDELTA PE=1 SV=2
CmaG0022124.1	Phospholipase D gamma 1 OS=Arabidopsis thaliana OX=3702 GN=PLDGAMMA1 PE=1 SV=1
CmaG0003292.1	Phospholipase D zeta 1 OS=Arabidopsis thaliana OX=3702 GN=PLDZETA1 PE=1 SV=1
CmaG0012752.1	Phospholipase D zeta 1 OS=Arabidopsis thaliana OX=3702 GN=PLDZETA1 PE=1 SV=1
Phosphatidate phosphatase	
CmaG0009159.1	Phosphatidate phosphatase PAH1 OS=Arabidopsis thaliana OX=3702 GN=PAH1 PE=1 SV=1
CmaG0017861.1	Phosphatidate phosphatase PAH2 OS=Arabidopsis thaliana OX=3702 GN=PAH2 PE=1 SV=1
CmaG0020795.1	Probable lipid phosphate phosphatase beta OS=Arabidopsis thaliana OX=3702 GN=LPPB PE=2 SV=1
CmaG0014797.1	Putative lipid phosphate phosphatase 3, chloroplastic OS=Arabidopsis thaliana OX=3702 GN=LPP3 PE=2 SV=1
CmaG0002776.1	Lipid phosphate phosphatase 1 OS=Arabidopsis thaliana OX=3702 GN=LPP1 PE=2 SV=2
CmaG0002779.1	Lipid phosphate phosphatase 2 OS=Arabidopsis thaliana OX=3702 GN=LPP2 PE=2 SV=1
CmaG0002780.1	Lipid phosphate phosphatase 2 OS=Arabidopsis thaliana OX=3702 GN=LPP2 PE=2 SV=1
CmaG0008247.1	Lipid phosphate phosphatase delta OS=Arabidopsis thaliana OX=3702 GN=LPPD PE=2 SV=1
CmaG0006345.1	Lipid phosphate phosphatase epsilon 2, chloroplastic OS=Arabidopsis thaliana OX=3702 GN=LPPE2 PE=1 SV=1
CmaG0006346.1	Lipid phosphate phosphatase epsilon 2, chloroplastic OS=Arabidopsis thaliana OX=3702 GN=LPPE2 PE=1 SV=1
CmaG0009231.1	Lipid phosphate phosphatase gamma OS=Arabidopsis thaliana OX=3702 GN=LPPG PE=1 SV=1
Phosphatidylserine decarboxylase	
CmaG0003200.1	Phosphatidylserine decarboxylase proenzyme 1, mitochondrial OS=Arabidopsis thaliana OX=3702 GN=PSD1 PE=2 SV=1
CmaG0003731.1	Phosphatidylserine decarboxylase proenzyme 2 OS=Arabidopsis thaliana OX=3702 GN=PSD2 PE=2 SV=1
Dienoyl-CoA reductase	
CmaG0004818.1	Peroxisomal 2,4-dienoyl-CoA reductase OS=Arabidopsis thaliana OX=3702 GN=At3g12800 PE=2 SV=1

CmaG0020742.1	Peroxisomal 2,4-dienoyl-CoA reductase OS=Arabidopsis thaliana OX=3702 GN=At3g12800 PE=2 SV=1
Mitogen-activated protein kinase	
CmaG0018288.1	Mitogen-activated protein kinase 12 OS=Oryza sativa subsp. japonica OX=39947 GN=MPK12 PE=1 SV=2
CmaG0003112.1	Mitogen-activated protein kinase 16 OS=Arabidopsis thaliana OX=3702 GN=MPK16 PE=2 SV=2
CmaG0003115.1	Mitogen-activated protein kinase 16 OS=Arabidopsis thaliana OX=3702 GN=MPK16 PE=2 SV=2
CmaG0021347.1	Mitogen-activated protein kinase 19 OS=Arabidopsis thaliana OX=3702 GN=MPK19 PE=2 SV=2
CmaG0015852.1	Mitogen-activated protein kinase 20 OS=Arabidopsis thaliana OX=3702 GN=MPK20 PE=1 SV=2
CmaG0026054.1	Mitogen-activated protein kinase 3 OS=Arabidopsis thaliana OX=3702 GN=MPK3 PE=1 SV=2
CmaG0021980.1	Mitogen-activated protein kinase 4 OS=Arabidopsis thaliana OX=3702 GN=MPK4 PE=1 SV=2
CmaG0009693.1	Mitogen-activated protein kinase 9 OS=Arabidopsis thaliana OX=3702 GN=MPK9 PE=2 SV=2
CmaG0018465.1	Mitogen-activated protein kinase homolog MMK1 OS=Medicago sativa OX=3879 GN=MMK1 PE=1 SV=1
CmaG0006912.1	Mitogen-activated protein kinase homolog MMK2 OS=Medicago sativa OX=3879 GN=MMK2 PE=2 SV=1
CmaG0012462.1	Mitogen-activated protein kinase homolog NTF3 OS=Nicotiana tabacum OX=4097 GN=NTF3 PE=1 SV=1
CmaG0025039.1	Mitogen-activated protein kinase homolog NTF3 OS=Nicotiana tabacum OX=4097 GN=NTF3 PE=1 SV=1
CmaG0026074.1	Mitogen-activated protein kinase homolog NTF6 OS=Nicotiana tabacum OX=4097 GN=NTF6 PE=2 SV=1
CmaG0021091.1	Mitogen-activated protein kinase kinase 10 OS=Arabidopsis thaliana OX=3702 GN=MKK10 PE=1 SV=1
CmaG0023332.1	Mitogen-activated protein kinase kinase 2 OS=Arabidopsis thaliana OX=3702 GN=MKK2 PE=1 SV=2
CmaG0023334.1	Mitogen-activated protein kinase kinase 2 OS=Arabidopsis thaliana OX=3702 GN=MKK2 PE=1 SV=2
CmaG0015456.1	Mitogen-activated protein kinase kinase 3 OS=Arabidopsis thaliana OX=3702 GN=MKK3 PE=1 SV=1
CmaG0007547.1	Mitogen-activated protein kinase kinase 4 OS=Arabidopsis thaliana OX=3702 GN=MKK4 PE=1 SV=1
CmaG0019812.1	Mitogen-activated protein kinase kinase 5 OS=Arabidopsis thaliana OX=3702 GN=MKK5 PE=1 SV=2
CmaG0023458.1	Mitogen-activated protein kinase kinase 6 OS=Arabidopsis thaliana OX=3702 GN=MKK6 PE=1 SV=1
CmaG0009783.1	Mitogen-activated protein kinase kinase 9 OS=Arabidopsis thaliana OX=3702 GN=MKK9 PE=1 SV=1
CmaG0002619.1	Mitogen-activated protein kinase kinase kinase 1 OS=Arabidopsis thaliana OX=3702 GN=MEKK1 PE=1 SV=2
CmaG0021542.1	Mitogen-activated protein kinase kinase kinase 1 OS=Arabidopsis thaliana OX=3702 GN=MEKK1 PE=1 SV=2
CmaG0025686.1	Mitogen-activated protein kinase kinase kinase 1 OS=Rattus norvegicus OX=10116 GN=Map3k1 PE=1 SV=1
CmaG0000064.1	Mitogen-activated protein kinase kinase kinase 17 OS=Arabidopsis thaliana OX=3702 GN=MAPKKK17 PE=1 SV=1
CmaG0000067.1	Mitogen-activated protein kinase kinase kinase 17 OS=Arabidopsis thaliana OX=3702 GN=MAPKKK17 PE=1 SV=1
CmaG0000076.1	Mitogen-activated protein kinase kinase kinase 17 OS=Arabidopsis thaliana OX=3702 GN=MAPKKK17 PE=1 SV=1
CmaG0003654.1	Mitogen-activated protein kinase kinase kinase 17 OS=Arabidopsis thaliana OX=3702 GN=MAPKKK17 PE=1 SV=1
CmaG0003699.1	Mitogen-activated protein kinase kinase kinase 17 OS=Arabidopsis thaliana OX=3702 GN=MAPKKK17 PE=1 SV=1
CmaG0003701.1	Mitogen-activated protein kinase kinase kinase 17 OS=Arabidopsis thaliana OX=3702 GN=MAPKKK17 PE=1 SV=1
CmaG0004273.1	Mitogen-activated protein kinase kinase kinase 17 OS=Arabidopsis thaliana OX=3702 GN=MAPKKK17 PE=1 SV=1
CmaG0004541.1	Mitogen-activated protein kinase kinase kinase 17 OS=Arabidopsis thaliana OX=3702 GN=MAPKKK17 PE=1 SV=1
CmaG0006529.1	Mitogen-activated protein kinase kinase kinase 17 OS=Arabidopsis thaliana OX=3702 GN=MAPKKK17 PE=1 SV=1
CmaG0006572.1	Mitogen-activated protein kinase kinase kinase 17 OS=Arabidopsis thaliana OX=3702 GN=MAPKKK17 PE=1 SV=1
CmaG0006731.1	Mitogen-activated protein kinase kinase kinase 17 OS=Arabidopsis thaliana OX=3702 GN=MAPKKK17 PE=1 SV=1
CmaG0010390.1	Mitogen-activated protein kinase kinase kinase 17 OS=Arabidopsis thaliana OX=3702 GN=MAPKKK17 PE=1 SV=1
CmaG0015548.1	Mitogen-activated protein kinase kinase kinase 17 OS=Arabidopsis thaliana OX=3702 GN=MAPKKK17 PE=1 SV=1

CmaG0015549.1	Mitogen-activated protein kinase kinase kinase 17 OS=Arabidopsis thaliana OX=3702 GN=MAPKKK17 PE=1 SV=1
CmaG0016687.1	Mitogen-activated protein kinase kinase kinase 17 OS=Arabidopsis thaliana OX=3702 GN=MAPKKK17 PE=1 SV=1
CmaG0020780.1	Mitogen-activated protein kinase kinase kinase 17 OS=Arabidopsis thaliana OX=3702 GN=MAPKKK17 PE=1 SV=1
CmaG0021630.1	Mitogen-activated protein kinase kinase kinase 17 OS=Arabidopsis thaliana OX=3702 GN=MAPKKK17 PE=1 SV=1
CmaG0021632.1	Mitogen-activated protein kinase kinase kinase 17 OS=Arabidopsis thaliana OX=3702 GN=MAPKKK17 PE=1 SV=1
CmaG0021636.1	Mitogen-activated protein kinase kinase kinase 17 OS=Arabidopsis thaliana OX=3702 GN=MAPKKK17 PE=1 SV=1
CmaG0021638.1	Mitogen-activated protein kinase kinase kinase 17 OS=Arabidopsis thaliana OX=3702 GN=MAPKKK17 PE=1 SV=1
CmaG0021640.1	Mitogen-activated protein kinase kinase kinase 17 OS=Arabidopsis thaliana OX=3702 GN=MAPKKK17 PE=1 SV=1
CmaG0021648.1	Mitogen-activated protein kinase kinase kinase 17 OS=Arabidopsis thaliana OX=3702 GN=MAPKKK17 PE=1 SV=1
CmaG0021649.1	Mitogen-activated protein kinase kinase kinase 17 OS=Arabidopsis thaliana OX=3702 GN=MAPKKK17 PE=1 SV=1
CmaG0021652.1	Mitogen-activated protein kinase kinase kinase 17 OS=Arabidopsis thaliana OX=3702 GN=MAPKKK17 PE=1 SV=1
CmaG0021654.1	Mitogen-activated protein kinase kinase kinase 17 OS=Arabidopsis thaliana OX=3702 GN=MAPKKK17 PE=1 SV=1
CmaG0022875.1	Mitogen-activated protein kinase kinase kinase 17 OS=Arabidopsis thaliana OX=3702 GN=MAPKKK17 PE=1 SV=1
CmaG0024036.1	Mitogen-activated protein kinase kinase kinase 17 OS=Arabidopsis thaliana OX=3702 GN=MAPKKK17 PE=1 SV=1
CmaG0026110.1	Mitogen-activated protein kinase kinase kinase 17 OS=Arabidopsis thaliana OX=3702 GN=MAPKKK17 PE=1 SV=1
CmaG0026475.1	Mitogen-activated protein kinase kinase kinase 17 OS=Arabidopsis thaliana OX=3702 GN=MAPKKK17 PE=1 SV=1
CmaG0026524.1	Mitogen-activated protein kinase kinase kinase 17 OS=Arabidopsis thaliana OX=3702 GN=MAPKKK17 PE=1 SV=1
CmaG0001461.1	Mitogen-activated protein kinase kinase kinase 18 OS=Arabidopsis thaliana OX=3702 GN=MAPKKK18 PE=1 SV=1
CmaG0004534.1	Mitogen-activated protein kinase kinase kinase 18 OS=Arabidopsis thaliana OX=3702 GN=MAPKKK18 PE=1 SV=1
CmaG0005664.1	Mitogen-activated protein kinase kinase kinase 18 OS=Arabidopsis thaliana OX=3702 GN=MAPKKK18 PE=1 SV=1
CmaG0011221.1	Mitogen-activated protein kinase kinase kinase 3 OS=Arabidopsis thaliana OX=3702 GN=MAPKKK3 PE=1 SV=1
CmaG0016644.1	Mitogen-activated protein kinase kinase kinase 3 OS=Arabidopsis thaliana OX=3702 GN=MAPKKK3 PE=1 SV=1
CmaG0000071.1	Mitogen-activated protein kinase kinase kinase 5 OS=Arabidopsis thaliana OX=3702 GN=MAPKKK5 PE=1 SV=1
CmaG0006783.1	Mitogen-activated protein kinase kinase kinase 5 OS=Arabidopsis thaliana OX=3702 GN=MAPKKK5 PE=1 SV=1
CmaG0017826.1	Mitogen-activated protein kinase kinase kinase 5 OS=Arabidopsis thaliana OX=3702 GN=MAPKKK5 PE=1 SV=1
CmaG0004546.1	Mitogen-activated protein kinase kinase kinase A OS=Dictyostelium discoideum OX=44689 GN=mkkA PE=1 SV=2
CmaG0004569.1	Mitogen-activated protein kinase kinase kinase A OS=Dictyostelium discoideum OX=44689 GN=mkkA PE=1 SV=2
CmaG0003054.1	Mitogen-activated protein kinase kinase kinase NPK1 OS=Nicotiana tabacum OX=4097 GN=NPK1 PE=1 SV=1
CmaG0012837.1	Mitogen-activated protein kinase kinase kinase NPK1 OS=Nicotiana tabacum OX=4097 GN=NPK1 PE=1 SV=1
CmaG0002024.1	Mitogen-activated protein kinase kinase kinase YODA OS=Arabidopsis thaliana OX=3702 GN=YDA PE=1 SV=1
CmaG0009479.1	Mitogen-activated protein kinase kinase kinase YODA OS=Arabidopsis thaliana OX=3702 GN=YDA PE=1 SV=1
CmaG0018523.1	Mitogen-activated protein kinase kinase kinase YODA OS=Arabidopsis thaliana OX=3702 GN=YDA PE=1 SV=1

CmaG0021941.1	Mitogen-activated protein kinase kinase kinase YODA OS=Arabidopsis thaliana OX=3702 GN=YDA PE=1 SV=1
CmaG0008894.1	Mitogen-activated protein kinase-binding protein 1 OS=Xenopus laevis OX=8355 GN=mapkbp1 PE=2 SV=1
E3 ubiquitin ligase	
CmaG0004449.1	Probable E3 ubiquitin ligase SUD1 OS=Arabidopsis thaliana OX=3702 GN=SUD1 PE=1 SV=1
CmaG0013710.1	Probable E3 ubiquitin ligase SUD1 OS=Arabidopsis thaliana OX=3702 GN=SUD1 PE=1 SV=1
CmaG0018330.1	Probable E3 ubiquitin ligase SUD1 OS=Arabidopsis thaliana OX=3702 GN=SUD1 PE=1 SV=1
CmaG0020248.1	Probable E3 ubiquitin ligase SUD1 OS=Arabidopsis thaliana OX=3702 GN=SUD1 PE=1 SV=1
CmaG0017903.1	E3 ubiquitin ligase BIG BROTHER-related OS=Arabidopsis thaliana OX=3702 GN=BBR PE=2 SV=1
CmaG0019155.1	E3 ubiquitin ligase BIG BROTHER-related OS=Arabidopsis thaliana OX=3702 GN=BBR PE=2 SV=1
CmaG0009232.1	E3 ubiquitin ligase PQT3-like OS=Arabidopsis thaliana OX=3702 GN=At5g47430 PE=1 SV=1
CmaG0017217.1	E3 ubiquitin ligase PQT3-like OS=Arabidopsis thaliana OX=3702 GN=At5g47430 PE=1 SV=1
CmaG0020874.1	E3 ubiquitin ligase PQT3-like OS=Arabidopsis thaliana OX=3702 GN=At5g47430 PE=1 SV=1
CmaG0006448.1	E3 ubiquitin ligase rnf-5 OS=Caenorhabditis elegans OX=6239 GN=rnf-5 PE=1 SV=1
Abscisic acid insensitive (ABI) transcription factors	
CmaG0000220.1	ABSCISIC ACID-INSENSITIVE 5-like protein 2 OS=Arabidopsis thaliana OX=3702 GN=DPBF3 PE=1 SV=1
CmaG0002642.1	ABSCISIC ACID-INSENSITIVE 5-like protein 2 OS=Arabidopsis thaliana OX=3702 GN=DPBF3 PE=1 SV=1
CmaG0011901.1	ABSCISIC ACID-INSENSITIVE 5-like protein 2 OS=Arabidopsis thaliana OX=3702 GN=DPBF3 PE=1 SV=1
CmaG0017329.1	ABSCISIC ACID-INSENSITIVE 5-like protein 5 OS=Arabidopsis thaliana OX=3702 GN=ABF2 PE=1 SV=1
CmaG0004468.1	ABSCISIC ACID-INSENSITIVE 5-like protein 6 OS=Arabidopsis thaliana OX=3702 GN=ABF3 PE=1 SV=1
CmaG0007795.1	Protein ABSCISIC ACID-INSENSITIVE 5 OS=Arabidopsis thaliana OX=3702 GN=ABI5 PE=1 SV=1
Sec14-like protein	
CmaG0018132.1	Sec14 cytosolic factor OS=Schizosaccharomyces pombe (strain 972 / ATCC 24843) OX=284812 GN=sec14 PE=4 SV=1
UDP-sulfoquinovose synthase	
CmaG0017157.1	UDP-sulfoquinovose synthase, chloroplastic OS=Arabidopsis thaliana OX=3702 GN=SQD1 PE=1 SV=1
CmaG0017159.1	UDP-sulfoquinovose synthase, chloroplastic OS=Spinacia oleracea OX=3562 GN=SQD1 PE=1 SV=1
Triacylglycerol lipase (TAGL)	
CmaG0004360.1	Triacylglycerol lipase 1 OS=Arabidopsis thaliana OX=3702 GN=LIP1 PE=1 SV=1
CmaG0010457.1	Triacylglycerol lipase 2 OS=Arabidopsis thaliana OX=3702 GN=LIP2 PE=2 SV=1
CmaG0015584.1	Triacylglycerol lipase 2 OS=Arabidopsis thaliana OX=3702 GN=LIP2 PE=2 SV=1
CmaG0007969.1	Triacylglycerol lipase SDP1 OS=Arabidopsis thaliana OX=3702 GN=SDP1 PE=1 SV=1
CmaG0010423.1	Triacylglycerol lipase SDP1 OS=Arabidopsis thaliana OX=3702 GN=SDP1 PE=1 SV=1
Palmitoyl-protein thioesterase	
CmaG0000850.1	Palmitoyl-protein thioesterase 1 OS=Caenorhabditis elegans OX=6239 GN=ppt-1 PE=2 SV=2
CmaG0020854.1	Palmitoyl-protein thioesterase 1 OS=Caenorhabditis elegans OX=6239 GN=ppt-1 PE=2 SV=2
CmaG0020856.1	Palmitoyl-protein thioesterase 1 OS=Rattus norvegicus OX=10116 GN=Ppt1 PE=1 SV=1
Hydrolase-like Protein	
CmaG0000916.1	3-hydroxyisobutyryl-CoA hydrolase-like protein 1, mitochondrial OS=Arabidopsis thaliana OX=3702 GN=At3g60510 PE=1 SV=1
CmaG0010818.1	3-hydroxyisobutyryl-CoA hydrolase-like protein 2, mitochondrial OS=Arabidopsis thaliana OX=3702 GN=At4g31810 PE=1 SV=1
CmaG0023643.1	3-hydroxyisobutyryl-CoA hydrolase-like protein 3, mitochondrial OS=Arabidopsis thaliana OX=3702 GN=At4g13360 PE=1 SV=2

CmaG0015816.1	3-hydroxyisobutyryl-CoA hydrolase-like protein 5 OS=Arabidopsis thaliana OX=3702 GN=At1g06550 PE=1 SV=2
Acyl-CoA-binding protein	
CmaG0002266.1	Acyl-CoA-binding domain-containing protein 2 OS=Arabidopsis thaliana OX=3702 GN=ACBP2 PE=1 SV=1
CmaG0006640.1	Acyl-CoA-binding domain-containing protein 3 OS=Arabidopsis thaliana OX=3702 GN=ACBP3 PE=1 SV=1
CmaG0025271.1	Acyl-CoA-binding domain-containing protein 3 OS=Arabidopsis thaliana OX=3702 GN=ACBP3 PE=1 SV=1
CmaG0009437.1	Acyl-CoA-binding domain-containing protein 4 OS=Arabidopsis thaliana OX=3702 GN=ACBP4 PE=1 SV=1
CmaG0012809.1	Acyl-CoA-binding domain-containing protein 4 OS=Arabidopsis thaliana OX=3702 GN=ACBP4 PE=1 SV=1
CmaG0027511.1	Acyl-CoA-binding domain-containing protein 4 OS=Arabidopsis thaliana OX=3702 GN=ACBP4 PE=1 SV=1
CmaG0003400.1	Acyl-CoA-binding domain-containing protein 6 OS=Oryza sativa subsp. japonica OX=39947 GN=ACBP6 PE=2 SV=1
CmaG0023302.1	Acyl-CoA-binding protein OS=Fritillaria agrestis OX=64177 GN=ACABP PE=3 SV=1
CmaG0020974.1	Acyl-CoA-binding protein OS=Ricinus communis OX=3988 PE=3 SV=1
Phosphoenolpyruvate carboxylase	
CmaG0005993.1	Phosphoenolpyruvate carboxylase 2 OS=Flaveria trinervia OX=4227 GN=PPCC PE=1 SV=1
CmaG0024006.1	Phosphoenolpyruvate carboxylase 3 OS=Arabidopsis thaliana OX=3702 GN=PPC3 PE=1 SV=2
CmaG0019179.1	Phosphoenolpyruvate carboxylase 4 OS=Arabidopsis thaliana OX=3702 GN=PPC4 PE=2 SV=1
CmaG0013107.1	Phosphoenolpyruvate carboxylase kinase 1 OS=Arabidopsis thaliana OX=3702 GN=PPCK1 PE=1 SV=1
CmaG0018691.1	Phosphoenolpyruvate carboxylase kinase 1 OS=Arabidopsis thaliana OX=3702 GN=PPCK1 PE=1 SV=1
CmaG0022472.1	Phosphoenolpyruvate carboxylase, housekeeping isozyme OS=Glycine max OX=3847 GN=PPC16 PE=2 SV=1
LEAFY COTYLEDON2	
CmaG0005144.1	B3 domain-containing transcription factor LEC2 OS=Arabidopsis thaliana OX=3702 GN=LEC2 PE=1 SV=1
Trihelix transcription factor ASIL1	
CmaG0027118.1	Trihelix transcription factor ASIL1 OS=Arabidopsis thaliana OX=3702 GN=ASIL1 PE=1 SV=1
WRINKLED1	
CmaG0000202.1	Ethylene-responsive transcription factor WRI1 OS=Arabidopsis thaliana OX=3702 GN=WRI1 PE=1 SV=1
CmaG0000206.1	Ethylene-responsive transcription factor WRI1 OS=Arabidopsis thaliana OX=3702 GN=WRI1 PE=1 SV=1
CmaG0015266.1	Ethylene-responsive transcription factor WRI1 OS=Arabidopsis thaliana OX=3702 GN=WRI1 PE=1 SV=1
CmaG0019458.1	Ethylene-responsive transcription factor WRI1 OS=Arabidopsis thaliana OX=3702 GN=WRI1 PE=1 SV=1
FUSCA3	
CmaG0015663.1	B3 domain-containing transcription factor FUS3 OS=Arabidopsis thaliana OX=3702 GN=FUS3 PE=1 SV=2
CmaG0019909.1	B3 domain-containing transcription factor FUS3 OS=Arabidopsis thaliana OX=3702 GN=FUS3 PE=1 SV=2
PICKLE	
CmaG0010835.1	CHD3-type chromatin-remodeling factor PICKLE OS=Arabidopsis thaliana OX=3702 GN=PKL PE=1 SV=1
GLABRA2	
CmaG0006325.1	GLABRA2 expression modulator OS=Arabidopsis thaliana OX=3702 GN=GEM PE=1 SV=1
